# Contextual gating of whisker-evoked responses by frontal cortex supports flexible decision making

**DOI:** 10.1038/s41467-026-73622-y

**Published:** 2026-05-26

**Authors:** Parviz Ghaderi, Sylvain Crochet, Carl C. H. Petersen

**Affiliations:** Laboratory of Sensory Processing, Brain Mind Institute, School of Life Sciences, Ecole Polytechnique Fédérale de Lausanne (EPFL), Lausanne, Switzerland

**Keywords:** Decision, Barrel cortex, Sensory processing, Cortex, Premotor cortex

## Abstract

Context-dependent sensory processing underlies important aspects of flexible behavior. Here, we investigate how mice can use a briefly-presented auditory contextual Go or Nogo cue after a delay period to gate the transformation of a whisker deflection into licking for reward. Spatiotemporally-specific optogenetic inactivation demonstrated an important contribution of various cortical regions during distinct trial epochs, with only whisker secondary motor cortex (wM2) contributing strongly to all task epochs including auditory cue, delay and whisker stimulus. Electrophysiological recordings revealed prominent context representation in both wM2 and anterolateral motor cortex (ALM) in the form of persistent activity with stable population dynamics. Notably, we found that context and whisker sensory processing appeared to be integrated first in wM2, whose activity predicted future lick initiation in both correct hit trials and false alarm error trials already within 30 ms after whisker deflection. We thus identify wM2 as a key node for the contextual gating of the transformation of whisker sensation into motor commands for goal-directed licking.

## Abstract (web summary)

Sensory stimuli have different implications depending upon context. Here, the authors report prominent context-dependent integration of whisker sensation with auditory working memory cues in frontal cortex directly downstream of somatosensory cortex.

## Introduction

The appropriate action to take in response to a sensory stimulus often depends upon the context. Animals can learn such flexible decision-making policies through experience, but the neuronal mechanisms that support context-dependent transformation of sensory input into action remain poorly understood. Early studies in macaques found that the receptive field properties of individual neurons in the primary visual cortex changed according to the task demands^1^, for example the same visual stimulus evoked different action potential firing rates in some neurons depending on whether the monkey was performing a bisection or a vernier task^2^. Other studies have pointed to robust sensory coding in primary somatosensory cortex of macaques independent of task contexts, with correlates of perceptual decision-making gradually becoming more prominent along cortical hierarchies from secondary somatosensory cortex to premotor cortex^3,4^. Context-dependent sensory processing has also begun to be explored in mice, offering important opportunities for mechanistic investigations. For example, neurons in auditory cortex have been shown to change responses to pure tones depending upon whether mice were passively listening or actively responding to the same auditory stimulus^5^. Similarly, neurons in the mouse primary visual cortex respond differently to the same stimulus whether the mouse is engaged in a visual detection task or not^6^. However, very few studies have investigated how explicit context varying on a trial-to-trial basis could gate the response to the same stimulus in head-restrained mice. Two recent studies have addressed this question using delay match (or non-match) to sample tasks showing changes in sensory processing in primary and secondary sensory areas as a function of trial category (match or non-match) in one study^7^, and the importance of anterolateral motor cortex for the maintenance of contextual (sample) information during the delay between the sample and test stimuli in another study^8^.

Here, we designed a contextual task for head-restrained mice by providing an auditory contextual cue which determines the appropriate response to a tactile whisker stimulus. Mice have a highly-developed array of tactile whiskers which provides important information about the presence, location, shape and texture of objects in their immediate surroundings^9^. The neuronal circuits responsible for the early stages of whisker sensory processing have begun to be delineated^10^ and a variety of tasks for head-restrained mice have been developed to explore the mechanisms of whisker-dependent sensory decision making^11–16^. In these tasks, the subjective sensory percept is typically reported by licking and experimental investigations to date have explored the neuronal circuit mechanisms transforming whisker sensory input into goal-directed licking finding contributions from many cortical as well as subcortical brain regions^12,13,17–30^. Recently, a block-design rule-switching task was developed in which mice learned to lick in response to either a visual or a whisker stimulus depending upon reward contingency^31^. Here, we build upon these prior studies and develop a task in which mice receive an auditory contextual cue in every trial which needs to be remembered until a whisker stimulus is delivered determining whether the mouse should lick for reward. Through optogenetic inactivation and electrophysiological measurements of neuronal activity in trained mice, we find prominent context encoding in frontal cortex, with a specific subregion, the whisker-related secondary motor cortex (wM2), appearing to integrate context and whisker sensory signals to determine whether or not to lick.

## Results

### A short-term memory context-dependent whisker detection task

**Fig. 1:**
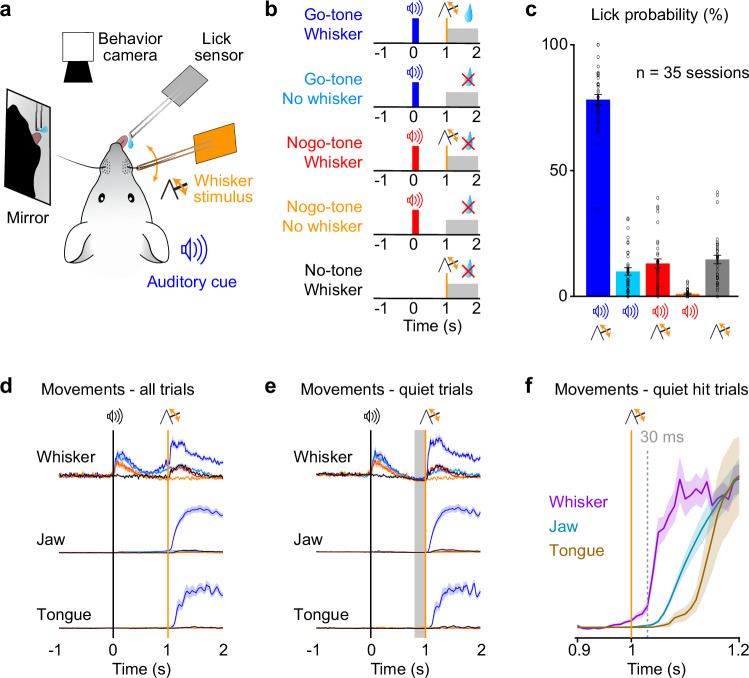
Behavioral characterization of a short-term memory context-dependent whisker detection task. **a** Schematic of experimental set-up with a thirsty head-restrained mouse receiving auditory and whisker stimuli accompanied by behavioral filming from two angles and monitoring of spout licking enabling appropriate water reward delivery. **b** One of five trial types was randomly presented with equal probability after ~10 s intertrial interval, with no licking allowed within the last 3.5–5 s. Trials were initiated by presentation of a pure tone for 100 ms (or No-tone), followed by a delay period during which licking was not allowed. 1 s after the tone onset, the C2 whisker was deflected for 5 ms in some trials but not others. Licking in the reporting window (gray shading) was rewarded only in Go-tone Whisker trials. **c** Behavioral performance assessed during electrophysiological recordings indicates that the probability of licking in the reporting window was high for rewarded Go-tone Whisker trials, but low in other unrewarded trial types. The bar shows the mean across the 35 sessions, with error bars indicating standard error of the mean (SEM) and circles showing the values for each individual session. **d** Whisking speed, jaw opening and tongue protrusion were quantified from high-speed videography across all correct trial types for 32 sessions (average across sessions, mean ± SEM; color-coded as in panels **b** and **c**; y-axis in arbitrary units). **e** All analyses of electrophysiological data (except where explicitly indicated) were from quiet trials, selected post hoc as trials in which neither the whisker nor jaw were moving in the last 200 ms of the delay period (gray shading) (trial type color-coded as in panels **b**–**d**; *y*-axis in arbitrary units). **f** Whisker movements began ~30 ms after the whisker deflection in Go-tone Hit trials, followed by jaw opening and subsequent tongue protrusion. Source data are provided as a Source Data file.

We designed a tactile detection task in which thirsty head-restrained mice learned to lick from a water reward spout when a whisker deflection was preceded by a Go-tone (Fig. 1a). Trials were initiated after a randomized intertrial interval of 10–11.5 s, only if mice had refrained from licking for at least 3.5–5 s. Licking was assessed online (and in all quantification of lick probabilities) as tongue-spout contacts measured through piezofilm attached to the reward spout. A pure tone (4 or 8 kHz, lasting 100 ms) embedded in continuous white noise served as the contextual cue. For each mouse, one tone was randomly selected as the Go-tone and the other as the Nogo-tone, and these remained fixed associations throughout training and task performance. After a delay period, the right C2 whisker was briefly (5 ms duration) deflected by a piezo actuator in some trials. During the delay period, the mouse was not allowed to lick the reward spout, which caused trial abortion. If the mouse licked the spout within a 1 s response window following a whisker stimulus that was preceded by a Go-tone, it received a drop of water ( ~ 5 µl) as reward. If the whisker deflection was preceded by a Nogo-tone or No-tone, then licking did not result in delivery of water reward. Equally, in trials without a whisker deflection, no reward was delivered (Fig. 1b). Mice learned to lick correctly in the reward-predicting trials including the Go-tone and whisker deflection (probability of licking correctly in the reporting window, P(lick) = 78 ± 2%, mean ± standard error of the mean, SEM, *n* = 35 sessions across *N* = 14 mice during electrophysiological recordings described later) and to withhold licking in the other unrewarded trial types: i) Go-tone without whisker deflection, P(lick) = 10 ± 1%; ii) Nogo-tone with whisker deflection, P(lick) = 13 ± 2%; iii) Nogo-tone without whisker deflection, P(lick) = 1 ± 0.3%; and iv) No-tone with whisker deflection, P(lick) = 15 ± 2% (Fig. 1c and Supplementary Fig. 1).

Orofacial movements were filmed from two angles with a high-speed camera and whisker, nose, jaw and tongue movements were quantified offline using DeepLabCut^32^. We found that the auditory contextual cue evoked whisking, which gradually decreased in amplitude over ~0.5 s during the delay period (Fig. 1d). In some trials with a Go-tone, but not other trial types, anticipatory whisking and jaw-opening developed prominently during the late delay period. Because movements correlate strongly with changes in neuronal activity^25,33–35^, throughout this study (except where specifically indicated) we chose to only analyze quiet trials, in which whisking and jaw movements were absent from the final 200 ms of the delay period (Fig. 1e). In quiet Hit trials, following the whisker deflection in Go-tone trials, mice rapidly initiated a sequence of movements starting with whisker protraction after ~30 ms (time to 20% max = 58 ± 8 ms), followed by jaw opening (115 ± 17 ms) and then protrusion of the tongue (231 ± 29 ms) (Fig. 1f) resulting in tongue-spout contact triggering water reward delivery with latency 330 ± 20 ms (*n* = 35 sessions during electrophysiological recordings) (Supplementary Fig. 1).

Mice can therefore learn a context-dependent short-term memory whisker detection task, with the 200 ms before whisker stimulation providing a period during which internal cognitive context signals associated with the auditory cues can be analyzed in the absence of orofacial movements. Equally, the 30 ms after whisker stimulation provides a period during which context-dependent processing of whisker sensory information takes place before any overt decision-related movements.

### Spatiotemporally-specific optogenetic inactivation

**Fig. 2:**
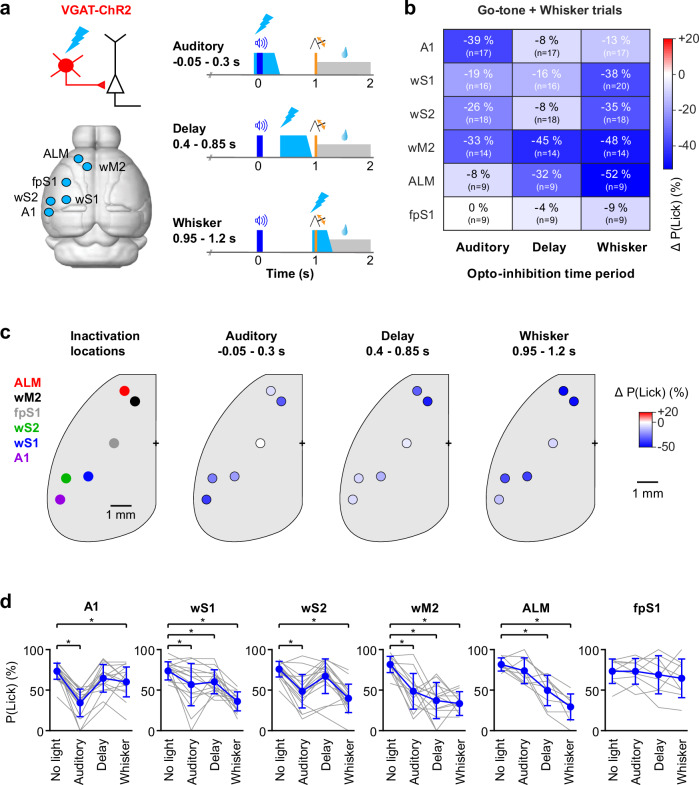
Spatiotemporally-specific optogenetic inactivation of cortex during the context-dependent whisker detection task. **a** Schematic indicating the three time windows for optogenetic inactivation via blue light application to cortical areas: primary auditory cortex (A1, N = 9 mice); whisker primary somatosensory cortex (wS1, N = 10 mice), whisker secondary somatosensory cortex (wS2, N = 10 mice), whisker secondary motor cortex (wM2, N = 9 mice), anterolateral motor area (ALM, N = 8 mice), and forepaw primary somatosensory cortex (fpS1, N = 8 mice) in VGAT-ChR2 mice expressing ChR2 in GABAergic neurons. **b** Average change in the probability of licking in the reporting window of Go-tone Whisker trials comparing no light trials to trials with blue light in one of the three inactivation time windows. The number of sessions for each time-window and area is indicated in parenthesis. **c** Color-coded spatial maps of the average change in lick probability for task epoch-specific inactivation in Go-tone Whisker trials. **d** Statistical analysis of the probability of licking in Go-tone Whisker trials for inactivation of different cortical areas in different trial epochs. Gray lines indicate individual sessions; Blue circles with error bars indicate mean ± SD. * *p* < 0.05, Light trials vs No light trials using paired bootstrap resampling for *n* = 9 sessions (1,000 iterations) followed by FDR correction within each stimulation window: A1-auditory *P* = 0.0004; A1-whisker *P* = 0.01; wS1-auditory *P* = 0.002; wS1-delay *P* = 0.0004; wS1-whisker *P* = 0.0003; wS2-auditory *P* = 0.0004; wS2-whisker *P* = 0.0003; wM2-auditory *P* = 0.0004; wM2-delay *P* = 0.0004; wM2-whisker *P* = 0.0003; ALM-delay *P* = 0.0004; ALM-whisker *P* = 0.0003. Source data are provided as a Source Data file.

To investigate the contributions of activity in different cortical regions to distinct task epochs, we applied blue light to transgenic mice expressing channelrhodopsin-2 (ChR2) in inhibitory neurons (VGAT-ChR2 mice^36^) allowing local suppression of activity in excitatory neocortical neurons with temporal specificity^25,37^. We focused on examining the effect of inactivating six different neocortical areas in the left hemisphere: i) the primary auditory cortex (A1); ii) the C2 whisker-related primary somatosensory barrel cortex (wS1); iii) the C2 whisker-related secondary somatosensory cortex (wS2); iv) the whisker-related secondary motor cortex (wM2); v) the anterolateral motor cortex (ALM); and vi) the forepaw-related primary somatosensory cortex (fpS1) (Fig. 2a). In each session, we chose one cortical region to inactivate in a randomly-selected one-third of each of the five different trial types during three different epochs: i) *Auditory* – from 50 ms before to 300 ms after the onset of the auditory cue; ii) *Delay* – from 400 ms after the start of the auditory cue to 150 ms before the end of the delay period; and iii) *Whisker* – from 50 ms before to 200 ms after the onset of the whisker deflection (with each inactivation period followed by a 100 ms ramp down period) (Fig. 2a). Inactivation reduced the probability of licking in the reporting window of Go-tone Whisker trials with a spatiotemporally-specific pattern (Fig. 2b-d and Supplementary Fig. 2), whereas inactivation had little impact upon other trial types (Supplementary Fig. 3). We did not carry out behavioral filming during the optogenetic inactivation experiments, so we were not able to distinguish different failure modes (e.g., decision vs motor), which would be of interest to investigate in future studies.

Because the number of inactivation sessions varied across cortical regions, we controlled for unequal sample sizes by subsampling each region to a common minimum number of 9 sessions across all comparisons. Statistical significance was assessed using paired bootstrap resampling (1,000 iterations), in which session-level lick probabilities from the no-light control and optogenetic inactivation conditions were resampled with replacement, and the statistic of interest was recomputed for each iteration. Bootstrap p-values were corrected for multiple comparisons using false discovery rate correction within each stimulation window.

For Go-tone Whisker trials, the largest impact of inactivation during the *Auditory* epoch was found for A1 (39 ± 4% reduction in lick probability, *p* = 0.0004, *n* = 17 sessions across *N* = 9 mice), but significant reductions were also found for inactivation of wM2 (33 ± 6% reduction in lick probability, *p* = 0.0004, *n* = 14 sessions across *N* = 9 mice), wS2 (26 ± 4% reduction in lick probability, *p* = 0.0004, *n* = 18 sessions across *N* = 10 mice) and wS1 (19 ± 6% reduction in lick probability, *p* = 0.002, *n* = 16 sessions across *N* = 10 mice). Importantly, inactivation of either ALM or fpS1 during the *Auditory* window did not cause any significant decrease in licking during the reporting period. The effect of inactivation during the auditory window was significantly stronger for wM2 than ALM (reduction in lick probabilities for wM2 = 33 ± 6 % vs ALM = 8 ± 5 %; bootstrap comparison, FDR-corrected, *p* = 0.009).

Inactivation during the *Delay* epoch of Go-tone Whisker trials caused a significant and large reduction in lick probability during the reporting window for wM2 (45 ± 7% reduction in lick probability, *p* = 0.0004, *n* = 14 sessions across *N* = 9 mice) and ALM (32 ± 5% reduction in lick probability, *p* = 0.0004, *n* = 9 sessions across N = 8 mice). Inactivation of wS1 also appeared to significantly, albeit more modestly, reduce licking (16 ± 3% reduction in lick probability, *p* = 0.0004, *n* = 16 sessions across *N* = 10 mice). On the other hand, inactivation of A1, wS2 or fpS1 during the *Delay* epoch did not significantly change lick probability in the reporting window.

Finally, all tested cortical regions, except fpS1, showed a significant reduction in licking probability when inactivation occurred during the *Whisker* stimulus epoch. The largest effects were found for inactivation of ALM (52 ± 4% reduction in lick probability, *p* = 0.0003, *n* = 9 sessions across *N* = 8 mice) and wM2 (48 ± 4% reduction in lick probability, *p* = 0.0003, *n* = 14 sessions across *N* = 9 mice), followed by wS1 (38 ± 3% reduction in lick probability, *p* = 0.0003, *n* = 20 sessions across *N* = 10 mice), wS2 (35 ± 4% reduction in lick probability, *p* = 0.0003, *n* = 18 sessions across *N* = 10 mice), and A1 (13 ± 3% reduction in lick probability, *p* = 0.01, *n* = 17 sessions across *N* = 9 mice).

Although optogenetic inactivation using blue light in VGAT-ChR2 mice has been reported to exert effects across a large spatial extent depending upon the precise stimulation parameters^38^, in our experimental paradigm we observed clear differences comparing inactivation of cortical regions separated by ~1 mm. For example, fpS1 is located ~1 mm anterior to wS1 and inactivation of fpS1 did not affect behavioral performance unlike inactivation of wS1, similar to previous experiments carried out under the same conditions^27^. Equally, during the presentation of the auditory contextual cue, here we found significant (bootstrap FDR-corrected, p = 0.009) differences in behavioral performance comparing inactivation of wM2 and ALM, which are also separated by ~1 mm. Thus, our optogenetic inactivation experiments appear to localize functional differences of neighboring cortical regions separated by relatively small distances, similar to previous experiments carried out under the same conditions in which inactivation of ALM and wM2 also had differential effects^25^. In future experiments, it will be important to further quantify the spatiotemporal impact of our inactivation paradigm.

Altogether, these inactivation experiments suggest the dynamic involvement of distinct cortical regions during different task epochs: i) A1 and wM2 contribute most importantly during the *Auditory* epoch; ii) wM2 and ALM contribute most importantly during the *Delay* epoch; and iii) ALM, wM2, wS1 and wS2 contribute importantly during the *Whisker* epoch. Apparently, neuronal activity in wM2 is key for correct execution of this context-dependent whisker detection task, being highly involved in all three task epochs.

### Spatiotemporal dynamics of neocortical activity

**Fig. 3:**
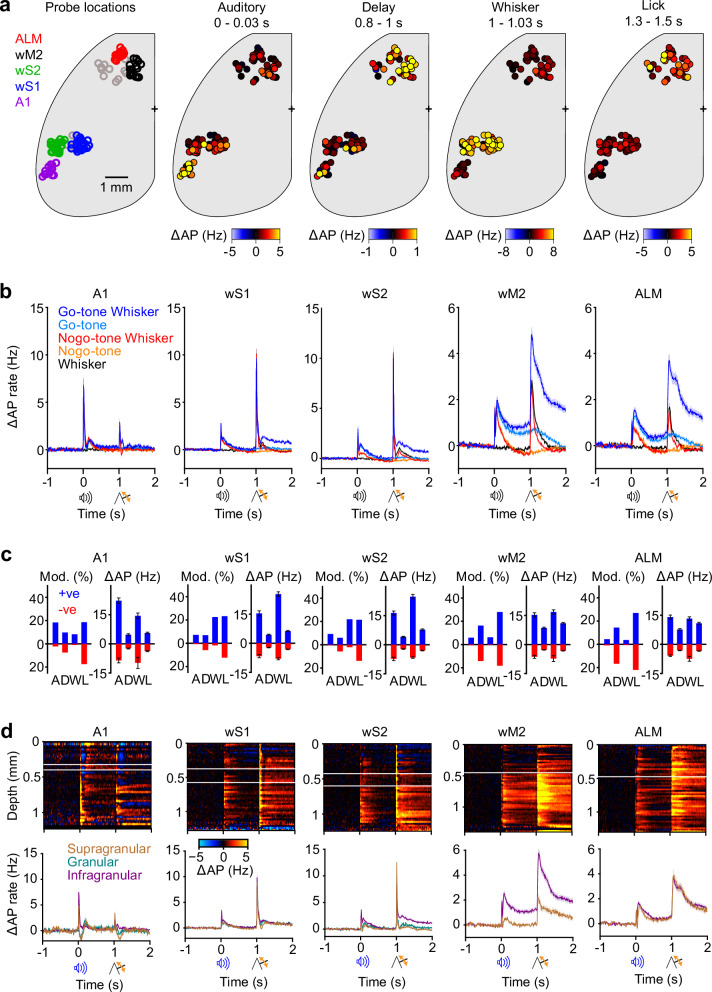
Spatiotemporal dynamics of neocortical activity during the context-dependent whisker detection task. **a** *Neuropixels* probes were targeted to A1, wS1, wS2, wM2 and ALM, followed ex vivo by anatomical reconstruction of probe tracks, with the cortical entry location indicated by a color-coded circle (*left*) (gray circles indicate probes that were not assigned to one of the selected cortical areas). Spatial activity maps averaged across all cortical neurons recorded on each probe and color-coded according to the change in firing rate (Δ AP) in Go-tone Whisker trials relative to pre-auditory stimulus baseline for Auditory and Delay period and relative to pre-whisker stimulus baseline for Whisker and Lick periods. **b** Peristimulus time histrograms (PSTHs) of neuronal activity in each cortical area across the five trial types including only correct trials (average across neurons, mean ± SEM; A1: 780 neurons; wS1: 1938 neurons; wS2: 2,064 neurons; wM2: 2749 neurons; ALM: 2763 neurons). **c** Receiver operate characteristic (ROC) analysis was used to quantify the fraction of significantly modulated neurons (increased firing in blue and decreased in red) and their change in firing rate for each area across different trial epochs (A, auditory; D, delay; W, whisker; and L, licking) in Go-tone Whisker trials compared to baseline (mean ± SEM). **d** *Top*, Neuronal activity in Go-tone Whisker trials across cortical areas computed with respect to their depth from the cortical surface reveals prominent delay period activity in deep, but not superficial, layers of wM2. *Bottom*, PSTHs computed separately for neurons located in the Supragranular, Granular and Infragranular layers for each area (average across neurons, mean ± SEM). Source data are provided as a Source Data file.

Given their apparent roles in task performance, we decided to target *Neuropixels*^39^ silicon probes to A1, wS1, wS2, wM2 and ALM for multisite high-density electrophysiological recordings of neocortical neuronal activity (Fig. 3a). After spike sorting using *Kilosort 2.0*^40^, quality control via *Bombcell*^41^, and anatomical localization of the fluorescently-labeled electrode tracks imaged ex vivo registered to a standard digital mouse brain atlas^42^, each identified unit was assigned a specific x,y,z-coordinate in the Allen CCF (Supplementary Figs. 4 and 5). Altogether, across 35 sessions in 14 mice, we recorded 10,294 well-isolated units located in: i) A1 - 780 units across 14 sessions in 6 mice; ii) wS1 - 1938 units across 24 sessions in 12 mice; iii) wS2 - 2064 units across 24 sessions in 11 mice; iv) wM2 - 2749 units across 18 sessions in 8 mice; and v) ALM - 2763 units across 16 sessions in 6 mice.

We first studied the overall spatiotemporal dynamics of neuronal activity averaged separately across the five different correctly executed trial types, selecting only quiet trials, as described in Fig. 1e, in which the mouse did not move its whisker or jaw in the 200 ms preceding whisker deflection. Visualized as the color-coded mean neocortical neuronal firing rate across each individual *Neuropixels* probe, different task epochs of correctly-executed Go-tone Whisker trials (i.e., Hit trials) were associated with different spatial patterns of neuronal activity (Fig. 3a). Averaged across the first 30 ms after the onset of the tone, the auditory cue evoked a fast excitation of neurons primarily in A1. Averaged across the last 200 ms of the delay period, elevated neuronal activity appeared most prominent in frontal areas wM2 and ALM. In the 30 ms following whisker deflection, sensory-evoked activity was primarily localized to wS1 and wS2. The period 300-500 ms after whisker stimulation was used to assess licking-related neuronal activity, which was most prominent in frontal regions. In further analyses, we characterized the firing dynamics averaged across units in each cortical region contrasting the five trial types (Fig. 3b and Supplementary Fig. 6). At the level of these grand averaged peristimulus time histograms (PSTHs), there were two obvious differences comparing Go-tone and Nogo-tone trials: i) there was a prominent elevation in firing rate in wM2 and ALM during the delay period of Go-tone trials compared to Nogo-tone trials; and ii) after the whisker stimulus there was a clear increase of activity in correct Go-tone Whisker trials compared to all other trial types in wS1, wS2, wM2 and ALM, presumably correlating with licking motor output, reward expectation and reward acquisition. We used ROC analysis to quantify the fraction of modulated units contrasting the firing of correct Go-tone Whisker trials in each cortical region during the same four trial epochs as described above: Auditory (0 – 0.03 s), Delay (0.8 – 1 s), Whisker (1 – 1.03 s) and Licking (1.3 – 1.5 s) relative to baseline activity (Fig. 3c). We carried out statistical tests to examine differences comparing wM2 and ALM for the Go-tone-related neuronal activity in the last 200 ms of the delay period of quiet trials, finding that the proportion of neurons that are positively modulated in our ROC analysis is statistically higher in wM2 compared to ALM. The fraction of up-modulated neurons was slightly but significantly higher in wM2 (16.0%) than in ALM (14.2%) (*p* = 0.0499, chi-squared test), whereas the fraction of down-modulated neurons was significantly lower in wM2 (14.3 %) compared to ALM (17.0 %) (*p* = 0.0062, chi-squared test). Both of these analyses support the overall stronger increased firing rate in wM2 compared to ALM in the late delay period of quiet trials following a Go-tone.

We next differentiated neuronal activity according to neuronal subtypes – fast-spiking (FS) units and regular-spiking (RS) units based on action potential duration–or cortical depth. On the whole, although FS neurons fired at much higher rates on average, the overall dynamics of FS and RS neurons were relatively similar compared within each cortical region for each trial type (Supplementary Fig. 7). More obvious differences appeared when comparing superficial and deep neocortical neurons (Fig. 3d and Supplementary Fig. 8). The prominent delay period activity on Go-tone trials in wM2 appeared to be absent from superficial layers of wM2 and almost exclusively represented in deeper neurons. However, delay period activity in ALM was equally prominent in superficial and deep neurons. Statistical tests indicated that neuronal activity during the late delay period of Go-tone trials differed significantly across cortical layers in wM2, with infragranular neurons having larger changes in firing rates than supragranular neurons (1.2 ± 0.1 Hz vs 0.03 ± 0.10 Hz; two-sample t-test, *p* = 1.7 × 10⁻⁷), whereas no significant differences were detected in A1, wS2, or ALM.

**Fig. 4:**
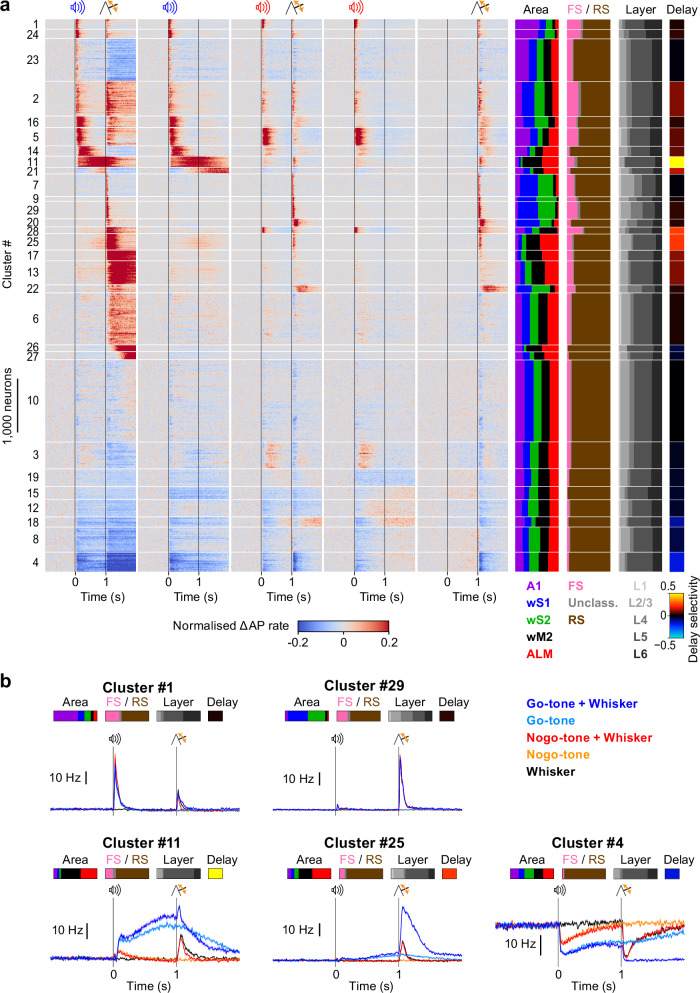
Diverse patterns of neuronal activity distributed across cortical regions, layers, and cell classes. **a** The averaged PSTHs for each recorded neuron in the five types of correctly executed trials were baseline subtracted, normalized, and clustered using a Gaussian mixture model after spectral embedding of the principal components, with the number of clusters determined by the minimum Bayesian Information Criterion. This identified 29 clusters each containing neurons with similar dynamics, but distributed across different cortical areas (A1, wS1, wS2, wM2, and ALM), different classes of neurons (RS, regular spiking; FS, fast spiking; Unclass, unclassified), different cortical layers, and with different context selectivity of firing in the last 200 ms of the delay period comparing Go-tone and Nogo-tone trials. **b** PSTHs of five selected clusters (mean ± SEM). Cluster #1 mainly represents the auditory cue, whereas Cluster #29 largely represents the whisker sensory response. Clusters #11 and #25 both show prominent Go-tone delay period excitation, whereas Cluster #4 contains neurons inhibited following the Go-tone.

These analyses reveal highly dynamic patterns of neuronal activity distributed across different cortical regions. However, it is also clear from visual inspection that different neurons within each region often fired with distinct temporal dynamics (Supplementary Fig. 4). We therefore clustered all the recorded neurons based on their average activity across the five correctly-executed trial types, finding altogether 29 clusters (Fig. 4a and Supplementary Fig. 9)^25^. For each cluster, we computed: i) the fraction of neurons in the different recorded cortical regions; ii) the distribution of RS, FS and unclassified units; iii) the layer distribution of the neurons; and iv) the delay period selectivity, computed from ROC analysis of the final 200 ms of the delay period contrasting correct Go-tone and Nogo-tone trials (Fig. 4a). Some clusters appeared to largely represent sensory responses (Fig. 4b), for example Cluster #1 had a large and fast response to the auditory cue and mostly consisted of neurons in A1, whereas Cluster #29 responded strongly and rapidly to whisker deflection and mostly consisted of neurons in wS1 and wS2. A few clusters showed strong context-dependent firing selectivity comparing Go-tone and Nogo-tone trials, most notably Clusters #11 and #25, which showed prominent delay period firing only in Go-tone trials and mainly consisted of neurons in wM2 and ALM (Fig. 4b). Interestingly, another cluster, Cluster #4, showed a prominent decrease in firing rate during the delay period selectively for Go-tone trials and neurons in this cluster were widely distributed across the five recorded cortical areas (Fig. 4b).

The neuronal activity associated with correct task execution therefore appears to be supported by highly-diverse firing dynamics across different trial types, different cortical regions, and different cortical layers. Altogether, neuronal activity in the quiet, late delay period comparing Go-tone vs Nogo-tone trials appears to be most obviously different for deep-layer neurons in wM2 (Fig. 3d), which also form a large fraction of the neurons of Cluster #11 (Fig. 4a, b).

### Decoding context from neuronal activity during the delay period

**Fig. 5:**
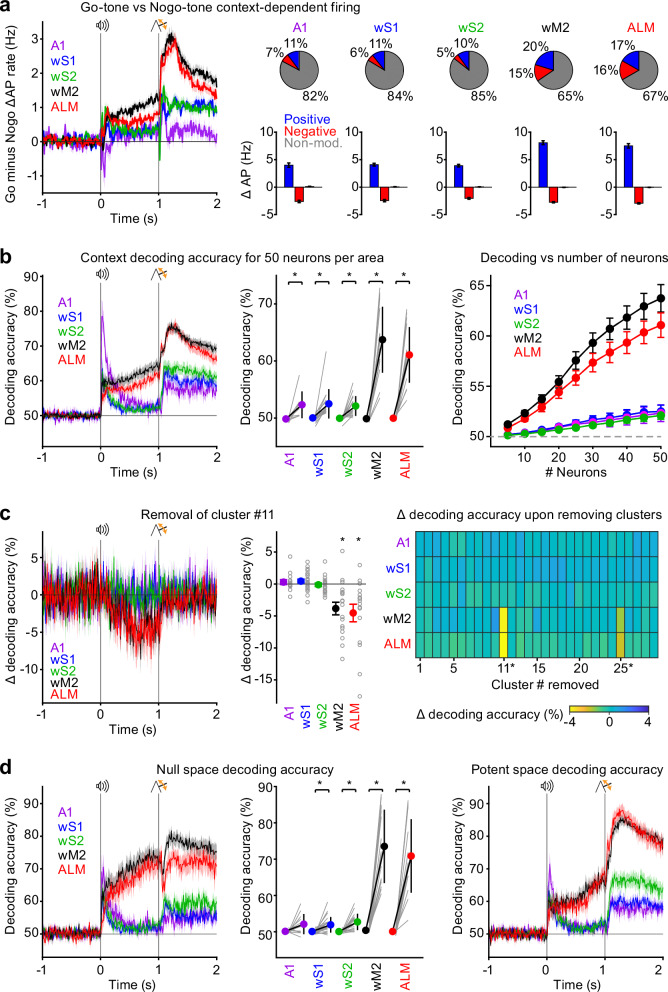
Context is prominently represented during the delay period in frontal cortex. **a** Difference in firing rate (Δ AP, average across neurons, mean ± SEM) comparing correct Go-tone Whisker and Nogo-tone Whisker trials across cortical areas (*left*). ROC analysis of differential firing in the last 200 ms of the delay period (*right*) showing the fraction of significantly modulated neurons (pie charts, *above*) and their change in firing (mean ± SEM, histograms, *below*). **b** Support Vector Machine (SVM) decoding of context comparing correct Go-tone Whisker and Nogo-tone Whisker trials for 50 simultaneously recorded neurons in each cortical area, with a new classifier trained for each 10 ms bin. *Left*, Mean decoding accuracy across time (mean ± SEM, average across sessions: A1, *n* = 8 sessions; wS1, *n* = 17 sessions; wS2, *n* = 19 sessions; wM2, *n* = 18 sessions; ALM, *n* = 16 sessions). *Center*, Decoding accuracy during the last 200 ms of the delay period indicates significant context information in each cortical area. Gray lines show individual sessions; plain circles with error bars show mean ± SD. *, *p* < 0.05, accuracy during delay vs baseline, two-sided Wilcoxon signed-rank test: A1 *P* = 0.008, wS1 *P* = 0.0003, wS2 *P* = 0.0003, wM2 *P* = 0.0002, ALM *P* = 0.0004. *Right*, Context can be much better decoded from the neuronal activity in frontal cortical areas wM2 and ALM, independent of the number of considered neurons (mean ± SEM). **c** Removing Cluster #11 strongly reduced context decoding in wM2 and ALM. *Left*, change in accuracy after removing Cluster #11 in each area (mean ± SEM, average across sessions: A1, *n* = 14 sessions; wS1, *n* = 24 sessions; wS2, *n* = 19 sessions; wM2, *n* = 18 sessions; ALM, *n* = 16 sessions). *Center*, quantification of the change in accuracy for each recording session upon removal of Cluster #11. Plain circles show mean ± SEM for each area. *, *p* < 0.05, two-sided Wilcoxon signed-rank test with FDR correction: wM2 *P* = 0.008; ALM *P* = 0.008. *Right*, Removal of Cluster #25 in wM2 or ALM also caused a significant, albeit small, decrease in decoding performance (wM2 *P* = 0.03; ALM *P* = 0.03), whereas removal of any other cluster did not significantly decrease the decoding accuracy (change in accuracy for each cluster and area; *, *p* < 0.05, two-sided Wilcoxon signed-rank test with FDR correction for each cluster). **d** Neuronal activity in a ‘movement null’ space for all trials (i.e., including both quiet trials and non-quiet trials with movements in the late delay period) also demonstrates delay period context coding in frontal cortex (mean ± SEM, average across sessions: A1, *n* = 12 sessions; wS1, *n* = 23 sessions; wS2, *n* = 19 sessions; wM2, *n* = 18 sessions; ALM, n = 16 sessions) (*left*), quantified for each recording session (*center*) (mean ± SD; *, *p* < 0.05, two-sided Wilcoxon signed-rank test: wS1 *P* = 0.0003, wS2 *P* = 0.0004, wM2 *P* = 0.0002, ALM *P* = 0.0004). The orthogonal ‘movement potent’ space also shows context coding (*right*). Source data are provided as a Source Data file.

We next quantified context-dependent neuronal firing in single neurons across different cortical regions. We contrasted correct Go-tone vs Nogo-tone trials with whisker stimuli forming the key Hit vs Correct Rejection trial types. Subtracting the firing rates showed prominent elevated firing in wM2 and ALM in Go-tone trials during the delay period (Fig. 5a). ROC analysis revealed neurons with significantly increased and decreased firing during the last 200 ms of the delay period comparing Go vs Nogo trials across cortical regions (Supplementary Fig. 10). Overall, we found the highest proportion of significantly modulated neurons in wM2 (20% of neurons increased firing on average by 8 Hz and 14% decreased firing by 3 Hz) and ALM (17% increased firing by 8 Hz and 16% decreased firing by 3 Hz). Thus, wM2 contained the largest proportion of Go-tone excited neurons during the delay period.

The activity patterns of simultaneously recorded neurons can contain additional information beyond what can be learned from analyzing single neurons. We therefore applied decoding analyses of neuronal network activity using support vector machines trained to classify correct Go vs Nogo trials with whisker stimuli (Fig. 5b). To enable a fair comparison across recordings, only sessions in which 50 or more neurons were simultaneously recorded from a given cortical region were included in the analysis and data were subsampled to 50 neurons per cortical area. A new decoder was trained on 80% of the trials for each 10 ms bin to enable optimal classification at each time point, with five-fold cross validation. Decoding in the baseline period was at chance level (50%) for all cortical regions, as expected. Immediately, upon presentation of the auditory contextual cue, the decoding of Go vs Nogo trials was highest in A1, but context could also be decoded from all other recorded cortical regions. The ability to decode context during the delay period rapidly decreased in A1, wS1 and wS2, but increased for wM2 and ALM. Focusing on the last 200 ms of the delay period, during which orofacial movements are absent, the auditory context could be decoded from the firing of 50 neurons in each area with accuracy varying across cortical regions: i) A1, 52 ± 1%, *p* = 0.008, *n* = 8 sessions across *N* = 6 mice; ii) wS1, 52 ± 1%, *p* = 0.0003, *n* = 17 sessions across *N* = 9 mice; iii) wS2, 52 ± 0.4%, *p* = 0.0003, *n* = 19 sessions across *N* = 9 mice; iv) wM2, 64 ± 1%, *p* = 0.0002, *n* = 18 sessions across *N* = 8 mice; and v) ALM, 61 ± 1%, *p* = 0.0004, *n* = 16 sessions across *N* = 6 mice. Decreasing the number of neurons per cortical region through further subsampling reduced decoding accuracy but maintained the overall conclusion that wM2 and ALM more strongly encode context compared to A1, wS1 and wS2.

To understand more about the underlying neuronal activity contributing to context decoding, we examined the impact of removing individual clusters (Fig. 5c). For most of the 29 clusters described above, there was no impact upon their removal for decoding accuracy evaluated during the late delay period. However, removal of Cluster #11 and Cluster #25 significantly decreased decoding accuracy for wM2 and ALM. Removal of Cluster #11 reduced decoding accuracy by 3.8 ± 1.0% (*p* = 0.008) for wM2 and by 4.5 ± 1.4% (*p* = 0.008) for ALM, with no significant effect upon A1, wS1 and wS2. Removal of Cluster #25 also reduced significantly the decoding accuracy by 1.6 ± 0.6% (*p* = 0.03) for wM2 and by 2.0 ± 0.6% (*p* = 0.03) for ALM.

In general, all analyses in this study were carried out on selected quiet trials in which orofacial movements were absent from the last 200 ms of the delay period. This precludes the involvement of orofacial motor or sensory reafference signals from contributing to our decoding analyses, but rejects around half of the recorded trials. Another useful approach, is to separate neuronal network activity into different subspaces associated either with ongoing movements (‘movement potent’ space) or the absence of movements (‘movement null’ space)^43,44^ and then specifically examine information available in each subspace. Following a recently developed analytical procedure^45^, we projected neuronal activity from each cortical region into a ‘null’ space not explored during movements (Supplementary Fig. 11), and carried out context decoding as before using a support vector machine (Fig. 5d). During the late delay period, in the 200 ms before whisker stimulus and using only ‘null’ space neuronal activity, we were again able to decode the context with varying success across cortical regions: i) A1, 52 ± 0.8%, *p* = 0.09; ii) wS1, 52 ± 0.4%, *p* = 0.0003; iii) wS2, 53 ± 0.5%, *p* = 0.0004; iv) wM2, 74 ± 2%, *p* = 0.0002; and v) ALM, 71 ± 3%, *p* = 0.0004.

In conclusion, context appears to be encoded strongly in frontal cortical regions wM2 and ALM even in the absence of ongoing movements as suggested by two independent analyses, either removing trials with movements (Fig. 5b) or by selectively examining neuronal activity in a ‘movement null’ space (Fig. 5d), with different contributions of RS and FS neurons (Supplementary Fig. 12).

### Context-dependent neuronal dynamics during the delay period

**Fig. 6:**
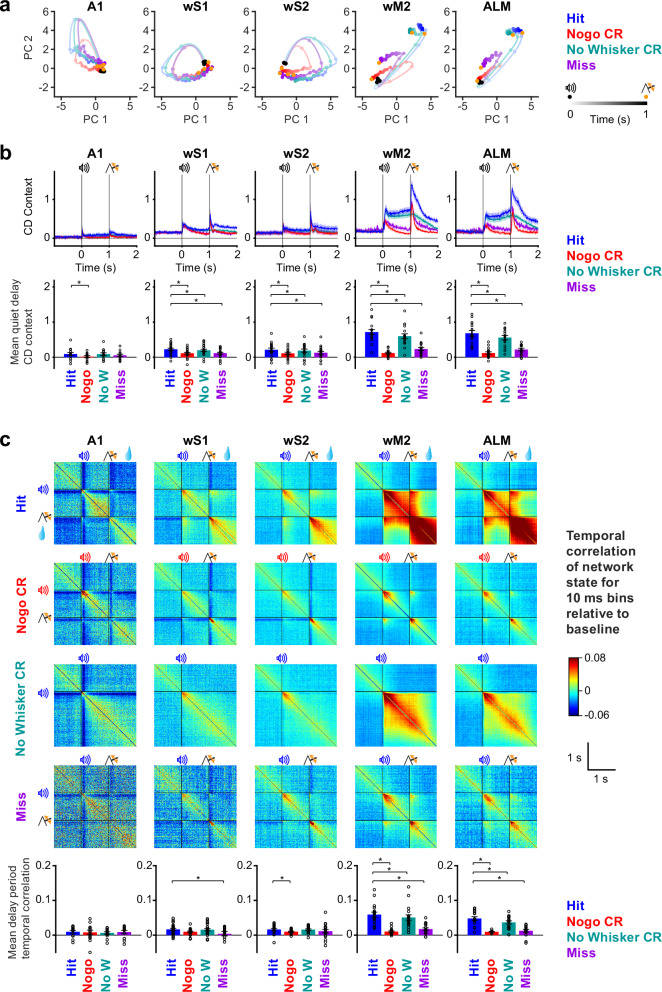
Attractor-like, persistent state dynamics encode context in frontal cortex. **a** The first two principal components accounting for neuronal activity of correct Go-tone Whisker and Nogo-tone Whisker trials were computed for different cortical areas in 50 ms time steps. Delay period activity for different trial types were projected into the PCA space showing the time between the onset of the auditory cue (black data point) and the time of the whisker stimulus (orange data point). Neuronal activity in PC1 and PC2 returned to baseline by the end of the delay period in A1, wS1 and wS2. However, activity in wM2 and ALM showed distinct trajectories for correct Go-tone trials (Hit and No-Whisker Correct Rejection) compared to correct Nogo-tone trials (Nogo Correct Rejection). Interestingly, error Go-tone Whisker trials where the mouse failed to lick (i.e., Miss trials) were associated with neuronal trajectories close to Nogo-tone trials. **b** A context coding direction (CD Context) was defined as the difference in neuronal state vectors between correct Go-tone Whisker and Nogo-tone Whisker trials averaged over the last 200 ms of the delay period. Projecting neuronal activity across different trial types indicated robust Go-context activity in the late delay period in wM2 and ALM for correct Go-tone trials, but reduced activity in Nogo-trials as well as Miss trials: *above*, time course (average across sessions, mean ± SEM); *below*, quantification for the last 200 ms of the delay period for each session. Bars with error bars show mean ± SEM; black circles show individual sessions. *, *p* < 0.05, Hit vs other trial types, two-sided Wilcoxon signed-rank test with Bonferroni correction for each area: A1 *n* = 14 sessions, Hit vs Nogo *P* = 0.002; wS1 *n* = 24 sessions, Hit vs Nogo *P* = 6 × 10^-5^, Hit vs NoW *P* = 0.02, Hit vs Miss *P* = 0.0002; wS2 *n* = 19 sessions, Hit vs Nogo *P* = 0.0006, Hit vs NoW *P* = 0.01, Hit vs Miss *P* = 0.0004; wM2 *n* = 18 sessions, Hit vs Nogo *P* = 0.0006, Hit vs NoW *P* = 0.001, Hit vs Miss *P* = 0.0006; ALM n = 16 sessions, Hit vs Nogo *P* = 0.001, Hit vs NoW *P* = 0.002, Hit vs Miss *P* = 0.001. **c** The network state vector at 10 ms resolution for each cortical area was correlated with all other times within the trial, averaged across trials of the same type for each session, and then averaged across sessions after subtraction of the mean correlation during the baseline (1 s before auditory tone). In correct Go-tone trials, a period of highly correlated activity emerges in wM2 and ALM in the delay period, which is absent from Nogo-tone trials and also Miss trials (temporal correlation color maps, *above*). Quantification of the mean correlation during the delay period revealed significantly increased correlation of population activity in correct Go-tone trials in wM2 and ALM (*below*). Bars with error bars show mean ± SEM; black circles show individual sessions. *, *p* < 0.05, Hit vs other trial types, two-sided Wilcoxon signed-rank test with Bonferroni correction for each area: A1 *n* = 14 sessions; wS1 *n* = 24 sessions, Hit vs Miss *P* = 0.006; wS2 *n* = 19 sessions, Hit vs Nogo *P* = 0.047; wM2 n = 18 sessions, Hit vs Nogo *P* = 0.0006, Hit vs NoW *P* = 0.02, Hit vs Miss *P* = 0.0009; ALM *n* = 16 sessions, Hit vs Nogo *P* = 0.002, Hit vs NoW *P* = 0.001, Hit vs Miss *P* = 0.0004. Source data are provided as a Source Data file.

We next investigated the temporal evolution of neuronal activity during the delay period. We first considered the neuronal dynamics through dimensionality reduction via principal component analysis (PCA) computed across correct Go and Nogo trial types and then projected the activity of other trials into the same PC space (Fig. 6a). Neuronal activity in each cortical area exhibited a rapid response after tone onset in PC1 (explained variance: A1, 11%; wS1, 11%; wS2, 11%; wM2, 17%; and ALM 14%) and PC2 (explained variance: A1, 6%; wS1, 6%; wS2, 6%; wM2, 8%; and ALM 8%). Activity in A1, wS1 and wS2 followed an apparently cyclic trajectory, returning near to the start point by the end of the delay period. However, in correct Go-tone trials, neuronal activity in wM2 and ALM settled rapidly (within ~200 ms) into an apparent fixed point far from the origin, as visualized in the first two PC dimensions, consistent with attractor state dynamics (Supplementary Fig. 13). As expected, the same apparent neuronal state was reached during the delay period in correct Go-tone trials with and without whisker stimulus. Correct Rejection trials in which a Nogo-tone was presented resulted in a rapid return to baseline activity in PC1 and PC2 dimensions. Interestingly, in error trials where the mouse did not lick in response to the whisker stimulus preceded by a Go-tone (i.e., Miss trials), the neural activity in wM2 and ALM failed to reach the apparent attractor state of correct Go-tone trials.

In a separate analysis, we computed a context coding direction by subtracting the time-averaged neuronal state vector of the last 200 ms of the delay period in correct Go-tone trials from correct Nogo-tone trials (Fig. 6b). For each session and each cortical area, we projected the neuronal activity in the context coding direction for different trial types and quantified across the last 200 ms of the delay period. Activity in the context coding dimension was high in wM2 and ALM throughout the delay period in correct Go-tone Hit trials, but strongly reduced in the incorrect Go-tone Miss trials.

Finally, we directly examined the temporal dynamics by correlating 10 ms time-slices of the neuronal network state vector of simultaneously recorded neurons within each cortical area (Fig. 6c and Supplementary Fig. 14). To compute these temporal correlations, we first computed the temporal correlation for each single trial, then averaged across trials of the same type for each session, and then averaged across different recording sessions after subtracting the baseline correlation. Presentation of the auditory cue evoked a brief increase in correlated neuronal activity in A1, wS1 and wS2. However, in wM2 and ALM, the Go-tone induced a long-lasting increase in correlated neuronal activity during the delay period in both Hit and Go-tone No-Whisker Correct Rejection trials. In contrast, presentation of the Nogo-tone failed to induce network states with high temporal correlation. Importantly, the highly-correlated network state during the delay period was also absent in Miss trials, where the mice failed to lick in response to the whisker stimulus preceded by a Go-tone.

In summary, the Go context appears to be encoded by a stable neuronal network state with persistent activity in frontal cortical areas wM2 and ALM. No equivalent dynamics appear to be associated with the Nogo context. That the Go-tone induced attractor state in wM2 and ALM is absent in Miss trials suggests that this network state might be necessary for correctly responding to the whisker stimulus after the Go-tone.

### Context-dependent processing of the whisker stimulus

**Fig. 7:**
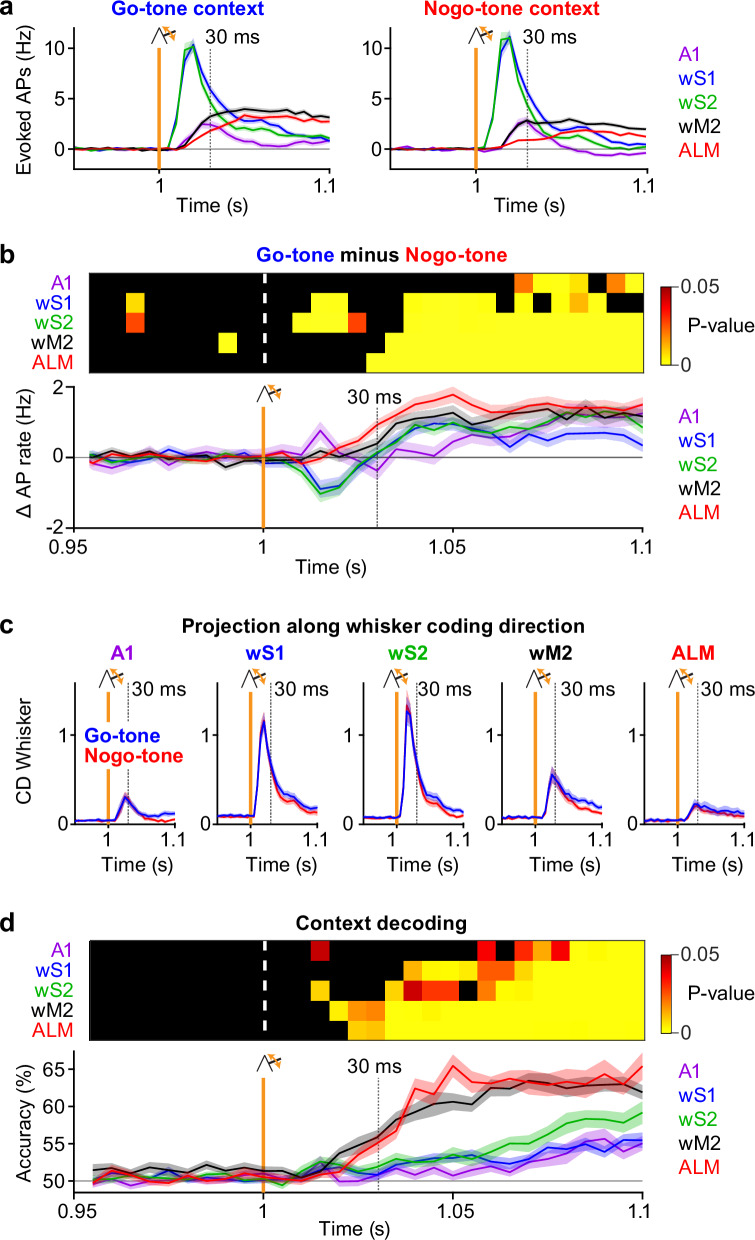
Context-dependent whisker-deflection evoked sensory responses. **a** PSTHs showing the baseline-subtracted (average over the 50 ms before whisker stimulus) whisker-deflection evoked activity at 5 ms resolution across the cortical areas for Go-tone Whisker Hit trials (*left*) and Nogo-tone Whisker Correct Rejection trials (*right*) (mean ± SEM; average across neurons). **b** Subtracting the PSTHs for the two conditions reveals transient significantly reduced evoked activity in wS1 and wS2 in Go-tone context around 15 ms, and higher activity later (starting ~30 ms) in most areas. Color map shows *P* values for each area and time bin (two-sided Wilcoxon signed-rank test with FDR correction for each area: A1 *n* = 780 neurons; wS1 *n* = 1938 neurons; wS2 *n* = 2064 neurons; wM2 *n* = 2749 neurons; ALM *n* = 2763 neurons). The PSTHs show mean delta firing rate averaged across neurons for each area (solid line) ± SEM (shading). **c** A whisker coding direction (CD Whisker) was defined as the difference in neuronal state vectors between the first 30 ms after the whisker stimulus and the baseline from correct No-tone Whisker trials. Projecting neuronal activity into this CD Whisker for correct Go-tone and Nogo-tone Whisker trials indicated no significant contextual changes (mean ± SEM, average across sessions). **d** Context-decoding of the baseline-subtracted neuronal activity reveals the first persistently significant context-decoding in the sensory-evoked response in wM2 at 20 ms, with ALM following shortly afterwards. Color map shows *P* values for each area and time bin (two-sided Wilcoxon signed-rank test with FDR correction for each area: A1, *n* = 14 sessions; wS1, *n* = 24 sessions; wS2, *n* = 19 sessions; wM2, *n *= 18 sessions; ALM, *n* = 16 sessions). The PSTHs show mean accuracy averaged across sessions for each area (solid line) ± SEM (shading). Source data are provided as a Source Data file.

Apparently, the Go-tone sets the neuronal network in a stable attractor state from which neuronal dynamics can evolve to initiate licking in response to whisker deflection. We therefore next began to evaluate the interaction of context and whisker stimulus evoked activity. We first considered the change in single neuron firing rates in 5 ms time bins evoked by the whisker stimulus relative to a baseline computed across the 50 ms immediately before the whisker deflection (Fig. 7a&b and Supplementary Figs. 15 and 16). Comparing correct trials in which the whisker stimulus was preceded by a Go-tone or a Nogo-tone, we found significant changes in evoked activity, with an early transient reduction in the whisker response in wS1 and wS2 for Go-tone trials, followed by an overall increased activity in most cortical regions at later times, presumably accompanied by various orofacial movements which begin after 30 ms (see Fig. 1f). However, no significant context differences were found in analyses of a whisker sensory response coding direction based on the 30 ms of activity evoked by whisker stimuli without a preceding tone cue and without subsequent licking (Fig. 7c). Applying a support vector machine decoding strategy (as before, but now for 5 ms time bins with baseline subtraction) for classifying correct Go vs Nogo trials, we found that significant context-dependent sensory processing transiently appeared in A1 and wS2 at 15 ms after whisker stimulus for a single 5 ms-time bin; and appeared in a more lasting manner in wM2 at 20 ms after whisker stimulus (and all later time bins) and in ALM at 25 ms after whisker stimulus (and all later times) (Fig. 7d).

**Fig. 8:**
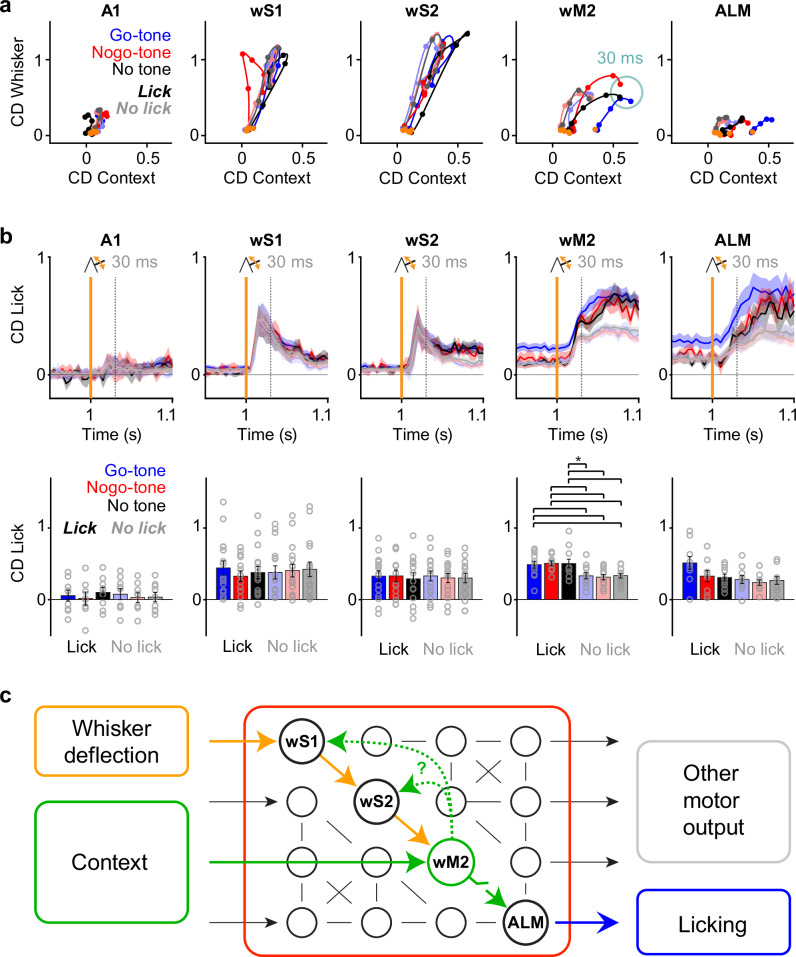
Context-dependent gating of whisker-to-lick sensorimotor transformation by frontal cortical region wM2. **a** Neuronal activity at 5 ms resolution from the onset of the whisker stimulus (orange dot) to 30 ms later was projected into the context (CD Context) and orthogonal whisker (CD Whisker) coding directions for each cortical area across different trial types. Dark colors represent trials in which the mouse licked in response to the whisker stimulus and light colors in which no licking was evoked. Only in wM2 do the trajectories of lick trials converge to a lick initiation zone (highlighted by a turquoise circle) clearly distinct from the no lick trials. **b** Quantification of neuronal activity in the lick coding direction (CD Lick) for lick and no lick trials in different contexts (*Top*, projected activity averaged across sessions, mean ± SEM). At 30 ms after the whisker deflection, trials with licking have significantly more activity in the wM2 lick coding direction compared to no lick trials. *Bottom*, bars with error bars show mean ± SEM and gray circles show individual sessions. *, *p* < 0.05, Kruskal-Wallis test, followed by LSD-corrected post hoc paired comparisons: A1 *n* = 8–9 sessions; wS1 *n* = 14–16 sessions; wS2 *n* = 14–16 sessions; wM2 *n* = 10–12 sessions, Kruskal-Wallis test *P* = 0.001, paired comparisons Go-tone Lick vs No lick trials *P* = 0.01, *P* = 0.006 and *P* = 0.02, Nogo-tone Lick vs No lick trials *P* = 0.004, *P* = 0.002 and *P* = 0.005, No-tone Lick vs No lick trials *P* = 0.03, *P* = 0.02 and *P* = 0.04; ALM n = 9-10 sessions. **c** A schematic model for context-dependent sensorimotor transformation. The auditory Go-tone is signaled to wM2, which maintains persistent activity throughout the delay period. Neurons in wM2 project back to wS1 and wS2 and could therefore provide signals for contextual modulation of whisker processing in sensory cortex. Conversely, feed-forward whisker sensory responses in wM2, perhaps signaled by direct monosynaptic inputs from sensory cortex, are integrated with contextual signals and, at 30 ms post-whisker stimulus, activity in wM2 segregates into lick and no lick states, which are subsequently passed onto licking motor regions such as ALM. Source data are provided as a Source Data file.

We next asked whether upcoming licking behavior occurring several hundred milliseconds after whisker stimulation, could be predicted from early neuronal activity. To isolate this decision-related signal from movement related activity, we analyzed the first 30 ms after whisker deflection. Neural activity during this window was projected onto two orthogonal axes: the context coding direction and the whisker coding direction (Fig. 8a). We compared trials that resulted in licking (correct Hits and False Alarms) with trials that did not result in licking (Correct Rejections and Misses). In A1, wS1, and wS2, lick and no-lick trajectories overlapped throughout the first 30 ms. In ALM, Hit trial type separation was evident, but lick-specific activity was not yet distinct. Only wM2 showed a clear divergence of trajectories into two distinct states corresponding to lick vs no-lick outcomes. This separation was primarily along the context coding direction. Single-session analyses confirmed that wM2 activity at 30 ms significantly distinguished lick from no-lick trials both in correct Go-tone and error Nogo-tone conditions (Supplementary Fig. 17). ALM showed a weaker effect, and no significant differences were observed in A1, wS1, or wS2. These analyses therefore point to wM2 integrating whisker sensory information in a context-dependent manner and making the first steps in the decision to lick already at 30 ms after whisker stimulus delivery.

Finally, we directly tested neuronal activity in a lick coding direction, which we defined during bouts of spontaneous licking not cued by any sensory stimuli. Projecting neuronal activity in the lick coding direction revealed separation between lick and no-lick trials for both correct and false alarm cases in wM2 at 30 ms after delivery of the whisker stimulus (Fig. 8b). The lick coding direction did not separate between lick and no-lick trials in A1, wS1 or wS2 at this early time point. Neuronal activity in ALM was higher in the lick coding direction only for correct trials, but false alarm lick trials were not different from no-lick trials at 30 ms post whisker stimulus time. These analyses therefore further support wM2 as a key cortical region that first predicts future lick initiation after the whisker stimulus independently of whether it is a correct or false alarm lick. Consistent with ALM also playing a prominent role in licking^46,47^, at later times, neuronal activity in the lick coding direction showed significant differentiation between lick and no-lick trials in ALM, wM2, wS2 and wS1 ranked according to the effect size quantified at 0.2-0.4 s after the whisker stimulus with ALM being largest (Supplementary Fig. 18).

## Discussion

We developed a novel context-dependent tactile detection task for head-restrained mice in which identical deflections of the C2 whisker were differentially processed to initiate goal-directed licking in the reward-predicting Go-tone context but to withhold licking in a Nogo-tone or a No-tone context. We established distinct contributions of neuronal activity in different cortical regions through spatiotemporally-specific optogenetic inactivation, and we measured neuronal activity through high-density extracellular electrophysiological recordings using *Neuropixels* probes finding diverse patterns of neuronal activity distributed across cortical areas.

The auditory context was a brief pure tone presented 1 s before the whisker deflection. In the late delay period just preceding whisker deflection and in the absence of orofacial movements, context was prominently represented in wM2 and ALM through persistent neuronal firing, especially in the deep cortical layers of wM2 (Figs. 3–6). This finding is generally in good agreement with several studies showing persistent activity in frontal cortical areas involved in different forms of short-term memory, when mice need either to postpone their response^25,48^ or to maintain information about a preceding sensory stimulus in a delayed match to sample task^8^. Interestingly, contextual information seems also to be represented in frontal areas in a block-designed rule-switching task in which the context is not explicitly indicated to the mice^31^. The contextual activity we observed during the delay period in wM2 and ALM was involved in correct task performance because optogenetic inactivation of these areas caused a profound reduction in the licking probability during the reporting period in Go-tone Whisker trials (Fig. 2). Whereas optogenetic inactivation of A1 and wM2 during the presentation of the Go-tone effectively disrupted task execution, inactivation of ALM did not (Fig. 2). Therefore, activity in wM2 appears to be the primary initiator of delay period context encoding and only subsequently recruits ALM, similar to the sequence of events underlying motor planning in a whisker-dependent object localization task^48^.

Auditory information could reach wM2 by many diverse neuronal pathways. Interestingly, previous work^49^ points to a direct pathway from auditory cortex to wM2, which is also supported by analysis of the Allen Mouse Brain Connectivity Atlas^50^ (Supplementary Fig. 19). However, there are many alternative routes including a potentially important pathway from retrosplenial cortex to wM2, which has recently been suggested to play a role in a closely-related context-dependent whisker detection task^51^. Input to wM2 via subcortical pathways is also likely to contribute importantly to relaying auditory information to frontal cortex, for example through a recently described pathway involving neurons in midbrain reticular and pedunculopontine nuclei signaling to a thalamic region projecting to frontal cortex^52^. Future studies are therefore necessary to specifically investigate the synaptic circuit mechanisms underlying the origin of the auditory contextual signals observed in wM2 in our context-dependent whisker-detection task.

In our task, the Go-tone therefore sets the neuronal dynamics in a state from which whisker deflection is more likely to initiate a licking motor command. Optogenetic inactivation during the whisker stimulation revealed the contributions of multiple cortical regions including wS1, wS2, wM2 and ALM, which presumably interact to generate a licking motor command (Fig. 2). The earliest difference in whisker sensory processing was a reduction in evoked activity in wS1 and wS2 at ~15 ms after whisker deflection (Fig. 7). Top-down input from wM2, which is known to innervate wS1 and wS2, could influence the whisker response in these early sensory cortical regions, and in future experiments it will be of interest to directly examine this hypothesis (Fig. 8c). The key context-dependent integration of whisker sensation however appears first in wM2 and subsequently spreads to ALM (Fig. 7d). Neurons in whisker sensory cortex project to whisker motor cortex^53–56^, and we hypothesize that direct monosynaptic input from sensory cortex is integrated in postsynaptic neurons in wM2 in a context-dependent manner depending upon the delay period activity. Indeed, within 30 ms of whisker deflection, activity in wM2 predicts licking in response to whisker deflection for both correct Hit and error False Alarm trials (Fig. 8a, b). Neurons in wM2 therefore serve to gate the processing of the whisker stimulus in a context-dependent manner (Fig. 8c). Distinct classes of excitatory neurons in wM2 project to many diverse cortical and subcortical targets, including ALM, which might contribute importantly. In future studies, it will be important to further define the essential subtypes of neurons in wM2 and which synaptic interactions drive the context-dependent gating of sensory processing in wM2. It is important to note, that many other brain regions including thalamus, basal ganglia, hypothalamus, hippocampus, midbrain and brainstem are also likely to contribute.

## Methods

### Animals

All procedures were approved by the Swiss Federal Veterinary Office (License number VD-3769) and were conducted in accordance with the Swiss guidelines for the use of research animals. Optogenetic inactivation experiments were conducted in VGAT-ChR2 mice [B6.Cg-Tg(Slc32a1-COP4*H134R/EYFP)8Gfng/J, JAX: 014548]^36^. Some of these mice were later used for high-density extracellular recordings. In addition, C57BL/6 wild type mice were used only for high-density extracellular recordings. Mice (male and female) were at least 6 weeks old at the time of head-post implantation (see below) and were kept in a reverse light/dark cycle (light from 7 pm to 7 am), in ventilated cages at a temperature of 22 ± 2 °C with food available ad libitum. During behavioral training, water was restricted to 1 mL a day. All mice were weighed and inspected daily during behavioral training.

### Surgical procedures

Mice were anesthetized with a mixture of ketamine (125 mg/kg) and xylazine (10 mg/kg), administered intraperitoneally. To ensure perioperative analgesia, carprofen (100 µL at 0.5 mg/mL, i.p.) was administered before any incision, and postoperative pain was managed by supplementing ibuprofen (200 mg/L, Algifor Dolo Junior, Verfora SA, Switzerland) in the drinking water for three consecutive days. Throughout the surgery, body temperature was maintained at 37 °C using a closed-loop heating system (ThermoStar, RWD Life Science). Eyes were protected from drying using an ophthalmic ointment (Vita-Pos, Pharma Medica AG, Switzerland). The scalp was disinfected with povidone-iodine (Betadine, Mundipharma), and local anesthesia was provided by subcutaneous injection of a mixture of lidocaine (10 mg/kg) and bupivacaine (2.5 mg/kg) in a total volume of 20 µL. A portion of the scalp was removed to expose the skull, which was then cleaned and scraped gently to remove periosteal tissue. A thin layer of super glue (Loctite 401, Henkel) was applied across the cleaned skull surface, and a custom-fabricated lightweight head-post was affixed to the right hemisphere. The implant was further secured using self-curing dental acrylic (Paladur, Kulzer, Germany or Ortho-Jet, Lang, USA). A chamber was constructed over the left hemisphere providing optical access to the dorsal cortex through the transparent skull. Mice were carefully monitored throughout the recovery period, and their health status – including posture, body condition, mobility, and weight – was evaluated daily to ensure compliance with ethical guidelines.

After full recovery, intrinsic optical signal imaging was performed through the transparent skull under light isoflurane anesthesia (1.0–1.5% in O₂) to functionally localized the primary and secondary whisker somatosensory areas (wS1 and wS2) and the primary auditory cortex (A1). Frontal motor regions were localized using stereotaxic coordinates relative to bregma: the anteriolateral motor cortex (ALM): AP + 2.5 mm, ML + 1.5 mm) and secondary whisker motor cortex (wM2): AP + 2.0 mm, ML + 1.0 mm.

The day before the first recording session, 2–5 small craniotomies ( < 0.5 mm in diameter) were performed over the targeted cortical regions under isoflurane anesthesia (2–3% in O₂) to allow for the insertion of the *Neuropixels* probes. The craniotomies were placed unilaterally over the left hemisphere, and protected postoperatively with a silicone elastomer (Kwik-Cast, World Precision Instruments, USA) to maintain tissue integrity overnight.

### Mouse behavior

Head-fixed, water-restricted mice were trained to perform a context-dependent whisker detection task requiring short-term memory. Each trial began after a variable intertrial interval (10–11.5 s), which included a 3.5–5 s “No-lick” window to prevent premature responses. Trial start was delayed if licks occurred during this window. At trial onset, a 100 ms auditory cue (4 or 8 kHz pure tone) was presented, randomly assigned as either a “Go-tone” or a “Nogo-tone” across animals. After a fixed 1 s delay period, a brief whisker stimulus was delivered to the right C2 whisker, which was inserted into a glass capillary mounted on a piezoelectric actuator driven by a 10 ms cosine pulse (5 ms full width at half max). Mice were rewarded (5 µL water) only if they licked within a 1 s response window following the whisker stimulus that was preceded by the Go-tone. Licks during the delay period triggered trial abortion (“early licks”), and licks during the response window in other trial types (Nogo-tone, No-tone or No-Whisker) were classified as “False Alarms” and were not rewarded. To ensure mice used contextual cues and not just the whisker stimulus, five randomly interleaved trial types were used: i) Go-tone + Whisker; ii) Go-tone + No-Whisker; iii) Nogo-tone + Whisker; iv) Nogo-tone + No-Whisker; and v) No-tone + Whisker. The behavioral training protocol was structured in four progressive stages: Stage 1–Pre-training (Habituation): Mice were head-fixed and learned to associate licking the water spout with reward availability. Stage 2–Whisker Detection: Mice were trained to lick only in response to the whisker stimulus. Stage 3–Contextual Differentiation: Mice learned to discriminate between Go-tone and Nogo-tone trials and to delay their lick until after the whisker stimulus. This stage lasted 2–4 weeks and represented the most demanding phase. Stage 4–Full Task: The fifth trial type (No-tone + Whisker) was introduced to assess reliance on contextual cues for correct behavioral response.

### Optogenetic manipulations

To assess the role of specific cortical regions during distinct task epochs, optogenetic silencing was performed in VGAT-ChR2 mice, which express channelrhodopsin-2 (ChR2) in GABAergic interneurons. Blue light activation of these neurons effectively suppresses local cortical activity by increasing inhibitory drive^37^. Light was delivered using a 200 µm diameter optical fiber (0.22 NA, Thorlabs) positioned directly over the thinned skull region corresponding to the target area. A high-power 470 nm laser (MBL-F-473/200 mW, GMP SA, Switzerland) was used to provide pulsed stimulation at 100 Hz with a 50% duty cycle and a mean power of 12–15 mW. In each session, a single cortical region was targeted, with trial types randomly interleaved such that 30% of trials included light stimulation and 70% served as controls. Optogenetic inactivation was performed during one of three defined temporal epochs relative to the task: i) Auditory window: from 50 ms before auditory cue onset to 300 ms after auditory cue onset; ii) Delay window: from 400 ms after the cue to 150 ms before whisker stimulus onset; or iii) Whisker window: from 50 ms before to 200 ms after whisker stimulus. To avoid rebound excitation, each light pulse train ended with a 100 ms linear ramp-down (added to the time windows of inactivation described above). An ambient blue masking light was present throughout the behavioral sessions to prevent visual detection of inactivation trials. Targeted cortical areas included functionally mapped sensory regions – A1, wS1 and wS2 – as well as motor and frontal areas, wM2 and ALM, identified by stereotaxic coordinates. In addition, we also targeted the primary forepaw somatosensory cortex (fpS1), located ~1 mm anterior to wS1, as a control region. Inactivation trials were only conducted after mice achieved expert-level task performance, defined as a Hit rate above 80% and a False Alarm rate lower than 20%. Each session focused on a single cortical region, and the timing of light delivery (Auditory, Delay, or Whisker window) was randomized across trials.

### Electrophysiological recordings

To examine cortical dynamics with high temporal and spatial resolution, high-density extracellular recordings were performed using *Neuropixels* Phase 3 A probes^39^. Before insertion, the probes were coated with the lipophilic dye DiI (1,1’-dioctadecyl-3,3,3’,3’-tetramethylindocarbocyanine perchlorate, Invitrogen USA) for post hoc histological localization. Each recording session involved the insertion of 2–5 *Neuropixels* probes using a custom recording setup with motorized micromanipulators (Luigs & Neumann GmbH, Germany). Probes were inserted slowly to minimize tissue damage, targeting a depth of ~1500 µm relative to the pia. Insertion angles were adjusted according to each cortical area: vertical for frontal areas such as wM2 and ALM, ~30° from vertical for wS1, ~45° for wS2, and ~50° for A1.

Reference grounding was achieved using a silver wire connected to a Ag/AgCl electrode placed in the recording chamber filled with Ringer’s solution, serving as a stable and low-noise ground. Recordings were conducted in external reference mode using SpikeGLX software, with a local field potential (LFP) channel gain of 250 and action potential (AP) channel gain of 500. Following probe insertion, mice remained head-fixed for ~30 min to allow brain tissue and probes to stabilize before initiating the behavioral task and neural recordings. The recording environment was light-isolated and sound-shielded, with continuous ambient white noise played to minimize external disturbances. Across 34 sessions in 14 mice, recordings were performed from up to five brain regions simultaneously per session. This included 12 sessions with recordings from 5 regions, 3 sessions from 4 regions, 1 session from 3 regions, 16 sessions from 2 regions, and 2 sessions from a single region.

### Electrophysiology data analysis

Extracellular recordings were acquired from 130 *Neuropixels* channels sampled at 30 kHz for the AP band and 2 kHz for the LFP band. Signals were synchronized using TTL pulses via an NI PXIe-1071 chassis, ensuring precise alignment between neural activity, behavioral events and video filming.

Spike detection and clustering were performed using *Kilosort2.0*^40^, following common median referencing across channels. Putative spikes were detected based on amplitude thresholds and automatically sorted into units by template matching and clustering. Output from *Kilosort2* was subsequently curated using *Bombcell* (https://github.com/Julie-Fabre/bombcell), an automated spike sorting quality control tool that categorizes units based on waveform, firing rate, ISI violations, and noise contamination. Only units classified as single somatic neurons were included in further analysis. Final inclusion was limited to well-isolated units based on a combination of automated and manual quality metrics.

Neurons were categorized into regular spiking units (RSUs), fast spiking units (FSUs), or undefined units based on the spike waveform duration (peak-to-trough time). RSUs were defined as units with waveform duration > 0.46 ms, FSUs <0.36 ms, and neurons with intermediate values were classified as undefined. In most analyses, all neuron types (RSUs, FSUs, and undefined) were included, unless specifically mentioned.

To facilitate multimodal data integration and long-term reuse, all raw voltage signals, spike data, behavioral events, and DeepLabCut output were converted into the Neurodata Without Borders (NWB)^57^ format using custom MATLAB and Python scripts. A full NWB file was generated for each recording session, including spike times, waveform features, channel locations, behavioral timestamps, and video annotations. All files were validated with NWB Inspector.

### Histology and localization of electrode tracks

Following the completion of recording sessions, mice were deeply anesthetized and transcardially perfused with phosphate-buffered saline (PBS), followed by 4% paraformaldehyde (PFA; Electron Microscopy Sciences, USA) in PBS. Brains were extracted and post-fixed overnight at room temperature.

To visualize the trajectories of the *Neuropixels* probes, brains were sectioned coronally at 100 µm thickness using a vibratome (VT 1000S; Leica, Wetzlar, Germany). Each probe had been coated with the fluorescent dye DiI before insertion to allow subsequent identification of the probe tracks. Fluorescent images of the DiI-labeled tracks were acquired using an epifluorescence microscope (Leica DM5500). For anatomical alignment, all slices were processed using MATLAB-based Allen CCF tools (https://github.com/cortex-lab/allenCCF). Histological sections were first manually matched to their corresponding locations within the Allen Mouse Brain Atlas (CCF v3), correcting for deformation and individual brain size variation. Pre-processing included color balance and orientation correction. The software’s auto-alignment function was then used to match slice contours with the reference atlas. Manual control points were placed when necessary to improve accuracy. Once aligned, probe tracks and tips were manually annotated based on the DiI signal. To refine the depth and area assignment of each recording site beyond anatomical estimates, we used the *Universal Probe Finder* tool (https://github.com/JorritMontijn/UniversalProbeFinder). This tool allowed us to align probe locations by integrating histological data with electrophysiological markers such as spiking activity, responsiveness (ZETA scores), and multi-unit correlations, improving the precision of cluster-to-depth mapping. Each recording electrode was assigned a 3D coordinate and anatomical label corresponding to its position in the atlas. Each neuron was then assigned a 3D coordinate and anatomical label based on the main electrode defined by the amplitude of the spike waveform.

This rigorous post hoc localization ensured precise anatomical mapping of all recorded neurons, allowing downstream analyses to be linked to specific cortical areas and laminar depths. Both 2D coronal and 3D volumetric reconstructions were generated to provide detailed spatial context for interpreting neural activity across regions. Only neurons located within the neocortex were analysed in this study, with cortical area and layer assignments provided by the Allen Mouse Brain Atlas (CCF v3) parcellations.

### Behavioral data analysis

Lick events were detected online using a piezoelectric lick sensor, sampled at 2 kHz, and aligned to trial events using TTL pulses. Behavioral performance was analyzed based on licking responses during a defined 1 second response window, starting 1 second after the auditory cue (or at the expected cue time for No-tone trials). The first lick was used to determine trial classification (Hit, Miss, False Alarm or Correct Rejection), and its timing served as a behavioral readout of the tongue-spout contact time.

Trials were classified according to the presence or absence of a tone and/or whisker stimulus, resulting in five trial types: i) Go-tone Whisker; ii) Go-tone No-Whisker; iii) Nogo-tone Whisker; iv) Nogo-tone No-Whisker; and v) No-tone Whisker. Only licking within the response window in Go-tone Whisker trials was rewarded (Hit), whereas licking in other trial types was not (False Alarm). Early licks occurring during the delay between the trial start (auditory tone) and the onset of the response window (whisker stimulus) triggered immediate trial abortion and were excluded from analysis. Only completed trials–those without premature licking–were included in subsequent behavioral quantifications.

### Quantification of orofacial movements

To monitor orofacial movements during electrophysiological recordings, we used a high-speed camera (CL 600 X 2/M, Optronics, Germany) operating at 200 frames per second, with an exposure time of 0.5 ms and a resolution of 256 × 512 pixels. Infrared illumination enabled video acquisition without visual interference. The camera was mounted above the mouse, aligned to capture the motion of the left C2 whisker and the angle of the snout. A side mirror was placed adjacent to the setup to simultaneously image the tongue and jaw movements within the same video frame.

For pose estimation, we used DeepLabCut 2.2 b7, a deep learning-based toolbox for markerless motion tracking^32^. The network was trained on a combined dataset composed of randomly selected frames from Go-tone Whisker trials across all recording sessions. ~200 frames per session were manually labeled and the network was trained using the k-means extraction method for up to 300,000 iterations. After training, we extracted the x and y coordinates of the C2 whisker base and middle point, tongue tip, jaw tip, and snout angle across all video frames. Post-processing involved filtering out position estimates with likelihood scores below 60%. Whisker angular position was calculated as the angle between the whisker (from base to midpoint) and the midline of the mouse’s head. Jaw and tongue displacements were quantified as the distance between the tip of each structure and its respective resting position, defined as the mode of position during baseline.

In order to account for the impact of movement on neural activity, trials were classified as either “Quiet” or “Non-quiet” based on movement during the final 200 ms of the delay period. Quiet trials were defined as those in which jaw movement and whisker speed remained below the median plus one median absolute deviation (MAD) of the corresponding baseline values. This classification was used to control for movement-related neural modulation during sensory processing and decision-making periods.

### Data analyses

#### Clustering of neuronal responses

Neuronal activity during task performance is high-dimensional and exhibits considerable diversity, even among neurons within the same brain region. To reduce this complexity and identify meaningful patterns, neurons can be grouped based on their activity profiles across different task events using unsupervised clustering methods. Here, we clustered the activity of all recorded neurons using an established unsupervised method, as previously described^25,27^. For each neuron and each correct trial type (i.e., Hit and Correct Rejection trials), peristimulus time histograms (PSTHs) were computed (10 ms time resolution) over a 3 s window, which included 1 s of baseline, 1 s of delay, and 1 s of response period. To eliminate pre-stimulus firing rate biases, the PSTHs were baseline-subtracted. For each neuron, the mean PSTHs corresponding to each trial type were then concatenated along the time axis, enabling the analysis to capture response dynamics across multiple task conditions. To facilitate comparison across neurons with differing firing rate ranges, the concatenated PSTHs were amplitude-normalized by dividing its response vector by the difference between its maximum and minimum firing rates. This normalization ensured that clustering and dimensionality reduction analyses focused on temporal response patterns rather than absolute firing rate differences. The concatenated activity for all the neurons resulted in an activity matrix $X\in {{\mathbb{R}}}^{10294x1500}$ whose rows correspond to the concatenated normalized firing rate of each of the 10,294 recorded neurons across the different trial types (300 time bins x 5 trial types = 1500 time bins). To reduce the dimensionality of the neuronal activity matrix and facilitate effective clustering, we applied Principal Component Analysis (PCA). PCA transforms the original high-dimensional firing rate vectors into a set of orthogonal components, known as principal components, which are ranked by the amount of variance they explain in the data. This transformation reduces redundancy and highlights dominant patterns of activity across neurons. Seventeen principal components were retained for further analysis, as they captured the majority of variance in the population response. Projecting the original activity matrix into this reduced space preserved the essential structure of the data while substantially decreasing computational complexity. The resulting matrix *X* ’ $\in {{\mathbb{R}}}^{10294x17}$ provided a compact representation of neuronal dynamics suitable for downstream clustering. Spectral embedding was then employed to capture the complex, non-convex structure of the neuronal response data^58,59^. This technique involved constructing a similarity matrix based on the Euclidean distances between the neurons’ response patterns. The similarity matrix ${{{\rm{S}}}}\in {{\mathbb{R}}}^{10294x10294}$ whose element at row *i* and column *j* measures the similarity between ${x}_{i}^{{\prime} }$ and ${x}_{j}^{{\prime} }$ as:

$${s}_{{ij}}=\exp \frac{-{{{\rm{||}}}}{x}_{i}^{{\prime} }-{x}_{j}^{{\prime} }\,{{{{\rm{||}}}}}_{2}^{2}}{2{\sigma }^{2}}\in \left[0,1\right],$$

where $\sigma$ is a free parameter determining how local similarity is measured in the feature space and is tuned in order to have mean similarity values equal to 0.5 (the tuned value for $\sigma$ is 0.07975). The similarity matrix was then used to compute a normalized Laplacian matrix using the following equation:

$$L=I-{D}^{-0.5\,}W{D}^{-0.5\,},$$

where $I$ is the identity matrix, and $D$ is defined as ${{{\rm{diag}}}}\left({\left\{{\sum }_{k=1}^{10294}{s}_{{ik}}\right\}}_{i=1}^{10294}\,\right),$ which encodes the structure of the data in a way that highlights the natural clusters. The eigenvectors of this matrix *L* provided a new feature space that separated clusters of neurons more naturally. This transformation was crucial for accurately identifying clusters in the high-dimensional data, which often have complex, non-linear relationships that traditional clustering methods might miss. After excluding the very 1^st^ eigenvector, we extracted the next 16 eigenvectors of matrix $L$ as new feature matrix $\widetilde{X}\in {{\mathbb{R}}}^{10294\,\times 16}$ for next step of clustering. Clustering was performed in the reduced feature space using a Gaussian Mixture Model (GMM), a probabilistic framework that models the data as a mixture of multiple Gaussian distributions with unknown means and covariances. This approach allows for flexible modeling of complex data structures and accounts for uncertainty in cluster membership. To determine the optimal number of clusters, we used the Bayesian Information Criterion (BIC), which penalizes model complexity while rewarding goodness of fit. The BIC analysis indicated that 29 clusters provided the best trade-off between accuracy and parsimony. This clustering solution captured the diversity of neuronal response patterns while avoiding overfitting to noise or outliers in the data.

#### Receiver operating characteristic analysis and activity maps

To determine whether individual neurons significantly encoded task-relevant information, we applied Receiver Operating Characteristic (ROC) analysis to their spiking activity by comparing firing rates in different time windows or between different trial types. For each neuron, spike counts were computed in specific time windows and ROC curves were constructed by comparing the distribution of firing rates between the two conditions. The area under the ROC curve (AUC) was calculated, providing a measure of the neuron’s ability to distinguish between conditions. The AUC was then transformed into a Selectivity Index (SI) using the formula:

$${{{\rm{Selectivity}}}}\; {{{\rm{Index}}}}=2\times ({AUC}-0.5)$$

This transformation maps the AUC values into a scale ranging from −1 to 1. To assess statistical significance, the trial labels were shuffled 200 times to create a null distribution of AUC values, and a non-parametric permutation test was used to compare the real AUC against this shuffled distribution. Neurons were thus classified as Positively or Negatively modulated if they showed significantly higher or lower firing rate in one condition compared to the other and as Non-modulated if no significant difference was detected. To examine population-level encoding, we quantified the proportion of significantly modulated neurons in each brain area and computed grand-average PSTHs separately for positively and negatively modulated groups.

To visualize the average spatiotemporal dynamics of neuronal activity across all recording sessions, we constructed activity maps showing the mean change in population activity for each probe across four key time windows. For each window, we computed the trial- and cell-averaged activity of neurons recorded on each probe. The auditory window was defined as the 30 ms period immediately following auditory stimulus onset. The delay period was defined as the 200 ms preceding the whisker stimulus. The whisker window captured the first 30 ms following whisker stimulus onset. Finally, the lick period covered the time window from 300 ms to 500 ms after whisker stimulus onset.

#### Population decoding

To assess how well neuronal populations encode contextual information at the single-trial level, we performed time-resolved population decoding using linear support vector machines (SVMs). Neuronal activity was first binned into non-overlapping 10 ms windows, and for each time bin, a separate decoder was trained to classify trials as either Go-tone Whisker or Nogo-tone Whisker. Each population vector consisted of spike counts from simultaneously recorded neurons during that 10 ms window. To avoid class imbalance artifacts, we used random under-sampling of the majority class to ensure equal numbers of trials per condition before training the decoder. SVMs were implemented using the LIBSVM package (https://www.csie.ntu.edu.tw/~cjlin/libsvm/) with a linear kernel. Model parameters were optimized through grid search with nested cross-validation. Decoder performance was evaluated using 5-fold cross-validation. For each fold, the data was split into training (80%) and testing (20%) sets. The decoding accuracy was defined as the percentage of correctly classified trials in the test sets. To assess the statistical significance of decoding accuracy, we compared results to the baseline period (-1 s to 0 s before the auditory cue), using a Wilcoxon signed-rank test.

To account for differences in the number of recorded neurons across sessions and brain areas, we ran the decoder using a fixed number of neurons. Specifically, for each session, we randomly subsampled the specified number of neurons 100 times. For each subsample, we trained and tested the decoder using five-fold cross-validation. This resulted in 100 decoding accuracy values per session for the fixed neuron count. We then averaged these values to obtain a single performance measure, which we reported as the decoding accuracy for that specific number of neurons.

To assess the stability of the population dynamics during the delay period, we trained a fixed decoder using population activity averaged over the last 200 ms of the delay period and tested it across other trial epochs.

To account for the potential influence of preparatory movements during the delay period, we also performed context decoding using the movement-null subspace of neural activity. To achieve this, we first defined potent and null movement subspaces by following an approach inspired by Hasnain et al.^45^ and implemented using their publicly available codebase. For each session, we first computed a movement baseline by averaging the movement signal during the 1 s period prior to the auditory stimulus onset. This baseline distribution was used to set a movement detection threshold, defined as the mean plus one standard deviation of the baseline. We focused on the whisker speed signal. At each time point within a trial, a moment was labeled as ‘movement’ only if movement signals exceeded their respective thresholds; otherwise, it was labeled as ‘non-movement’. Using this binary movement mask, we projected the trial-by-trial neuronal activity into movement-null and movement-potent subspaces. The dimensionality of both subspaces was reduced to 10 dimensions, ensuring comparability across sessions. We then ran a context decoder on the 10-dimensional projection of the movement-null subspace using a linear support vector machine (SVM) classifier with five-fold cross-validation. This decoding procedure mirrored the same cross-validation and evaluation metrics used in our standard analyses, allowing us to directly compare the contributions of movement-related versus movement-independent neural activity to context representation.

#### Principal component analysis and coding directions

To analyze population dynamics, we first pre-processed each neuron’s activity across four trial types: Hit, Miss, Correct Rejection (Nogo-tone Whisker), and Correct Rejection (Go-tone No-Whisker). For each cell, we computed the trial-averaged activity with a 50 ms bin resolution and performed baseline subtraction using the 1-s baseline. The activity traces were then concatenated across conditions, and each neuron’s response was normalized by subtracting the mean and dividing by the range (max–min) of its activity. This resulted in a population matrix of size 10,294 neurons × 240 time bins, which we used for Principal Component Analysis (PCA) in the neuronal space.

To investigate the geometry and temporal dynamics of population coding during the task, we projected neural activity onto low-dimensional axes termed coding directions (CDs). These axes were defined to optimally separate trial types within the high-dimensional neuronal activity space.

For context-related analysis, we computed a Context Coding Direction (CD_Context_) to capture differences between Go-tone and Nogo-tone trials at the end of the delay period. The CD_Context_ was computed as the difference between the mean population vectors in the last 200 ms of the delay period for quiet Go-tone trials and Nogo-tone trials. The CD_Context_ was computed from 20% of trials and normalized to unit length. The population vector activity for each 10 ms time bin and each trial was then projected onto the CD_Context_ to obtain the projected population activity by computing the dot product between the CD vector and the population vector. This yielded a single scalar trajectory per trial, reflecting how strongly each trial aligned with the context coding axis over time.

To analyze the encoding of the whisker stimulus, we also defined a Whisker Coding Direction (CD_Whisker_) by comparing population activity vectors in the 30 ms window following the whisker stimulus to a 1-s baseline period in pure whisker trials (No-tone Whisker, Correct Rejection trials). The CD_Whisker_ was computed from 30% of the trials, orthogonalized to CD_Context_ using the Gram-Schmidt procedure, and then normalized to unit length. Activity from all trial types was then projected onto this axis to assess context-dependent modulation of early whisker-stimulus evoked responses. In addition, we examined how population dynamics evolved across time in different trials types by projecting the neuronal population activity onto both CD_Context_ and CD_Whisker_ simultaneously. These 2D projections revealed the trial-by-trial temporal trajectory of population responses and enabled us to visualize how contextual and sensory information were integrated over time.

We also defined a lick coding direction (CD_Lick_) using bouts of spontaneous licking that occurred outside of task trials and were not cued by any sensory stimulus. Spontaneous lick events were identified during inter-trial intervals and further restricted to lick bouts lasting longer than 0.5 s. For each neuron, firing activity in a post-lick window (0.2–0.5 s after lick onset), chosen to capture lick execution, was compared to activity in a pre-lick baseline window ( − 1 to −0.5 s before lick onset), selected to minimize contamination by lick-related activity. CD_Lick_ was defined as the difference between the averaged post-lick and pre-lick activity vectors, normalized to unit length, and used to project trial activity dynamics.

#### Temporal correlation analysis

To examine the evolution and stability of neuronal population dynamics during task performance, we analyzed temporal correlations in single-trial activity. Spike counts were computed in non-overlapping 10 ms bins across the 3-second trial duration, resulting in 300 time bins per trial. For each time bin, a population activity vector was constructed by concatenating the spike counts of all simultaneously recorded neurons in a given cortical area. Each vector thus represented the instantaneous population activity at that time point.

To quantify temporal correlations, we computed the Pearson correlation coefficients between all pairs of population vectors across the trial duration. This produced a 300 × 300 correlation matrix for each trial, where each element reflects the similarity of the neuronal population state between two time points. Diagonal elements of the matrix represent autocorrelations and are therefore equal to one. A separate correlation matrix was generated for each individual trial. To obtain a robust estimate of population dynamics for each condition, we averaged these matrices across all trials of the same type (e.g., Hit, Miss, and Correct Rejection trials), resulting in condition-specific temporal correlation maps. To correct for baseline correlations, we subtracted the mean of the off-diagonal elements from the 1-second pre-stimulus baseline period of each matrix. This baseline correction accounted for trial-invariant correlations present before trial onset. Finally, to obtain session-level estimates, we averaged the baseline-corrected matrices across all sessions for each brain region and trial type. The resulting correlation maps reflect how population activity evolves over time in response to different task events and how this evolution varies across cortical regions. This analysis provides insight into the temporal structure and dynamics of cortical processing, revealing how different brain areas maintain or transform neural representations over the course of a trial.

### Attractor-like state dynamics

To evaluate whether delay-period activity was maintained via an apparent attractor state, we quantified local population dynamics in a low-dimensional state space. For each cortical area and recording session, we selected quiet, completed Go-tone Whisker-Lick and No-go-tone Whisker-NoLick trials, baseline-subtracted spike counts using the baseline period ( − 1 to 0 s), and performed session-wise PCA on the trial-averaged population activity. We then projected single-trial activity onto the first two principal components (PC1–PC2) to obtain neural trajectories, and focused subsequent analyses on the delay period (0.8–1.0 s after the auditory cue). Within this analysis window, we estimated a vector field of population “velocity” in PC space by pooling observed positions and instantaneous velocities (ΔPC1, ΔPC2 between consecutive time bins) across trials. For each condition, the mean local velocity was computed on a 2D grid using Gaussian distance-weighted averaging of nearby samples, yielding a smooth estimate of the flow field. To compare dynamics between conditions, we computed the angle between the two condition-specific velocity vectors at each grid point (0° = aligned, 180° = opposite) and visualized this as an angle-difference map. To summarize this effect across sessions, we extracted a one-dimensional profile of angle difference along the line connecting the two condition-specific population states, normalized distance so that the two whisker points (Go and Nogo condition) corresponded to 0 and 1, averaged values within a narrow strip around this line, and finally reported the mean ± SEM across sessions for each area.

### Statistics

Data are presented as mean ± SEM unless otherwise indicated. Statistical analyses were conducted using MATLAB (MathWorks).

For comparisons between two paired conditions (e.g., decoding accuracy vs. baseline within the same session), we used the Wilcoxon signed-rank test. For unpaired comparisons between different groups (e.g., licking rates across trial types or neuron types), the Wilcoxon rank-sum test was applied. When comparing more than two groups (e.g., modulation indices across cortical regions), the Kruskal–Wallis test was used first, followed by pairwise comparison using a multiple comparison test (Fisher’s least significant difference procedure), where appropriate. We used the chi-squared statistic to compare proportions of neurons across areas.

In decoding and ROC analyses that involved time-resolved multiple bin comparisons, p-values were corrected for multiple comparisons using False Discovery Rate (FDR) correction.

Statistical significance thresholds were set at α = 0.05. For all results, the number of observations (e.g., neurons or sessions) is reported directly in the figures or figure legends to ensure transparency and reproducibility.

### Reporting summary

Further information on research design is available in the Nature Portfolio Reporting Summary linked to this article.

## Supplementary information

Supplementary Information

Reporting Summary

Transparent Peer Review file

## Source data

Source data

## Data Availability

The data described in this study are available via *Zenodo* at (10.5281/zenodo.17973874). Source data are provided with this paper.

## Code availability

MATLAB and Python code used for analyses are available via *Zenodo* at (10.5281/zenodo.17973874).
