# Author Correction: Contextual gating of whisker-evoked responses by frontal cortex supports flexible decision making

**DOI:** 10.1038/s41467-026-75986-7

**Published:** 2026-07-22

**Authors:** Parviz Ghaderi, Sylvain Crochet, Carl C. H. Petersen

**Affiliations:** Laboratory of Sensory Processing, Brain Mind Institute, School of Life Sciences, Ecole Polytechnique Fédérale de Lausanne (EPFL), Lausanne, Switzerland

**Keywords:** Decision, Barrel cortex, Sensory processing, Cortex, Premotor cortex

Correction to: *Nature Communications* 10.1038/s41467-026-73622-y, published online 26 May 2026

In this article, the author’s name Carl C. H. Petersen was incorrectly written as Carl. C. H. Petersen. The original article has been corrected.

