# Supplementary material for: Contextual gating of whisker-evoked responses by frontal cortex supports flexible decision making
Source: Nat Commun. 2026 May 26;17:5982. doi: 10.1038/s41467-026-73622-y (PMC13346651; doi:10.1038/s41467-026-73622-y)
Supplement: Supplementary file 1 — Supplementary Information [file 41467_2026_73622_MOESM1_ESM.pdf]

Contextual gating of whisker-evoked responses by frontal cortex supports flexible decision making

Parviz Ghaderi, Sylvain Crochet and Carl C.H. Petersen

Supplementary information consists of Supplementary Figures 1-19

Supplementary Figure 1

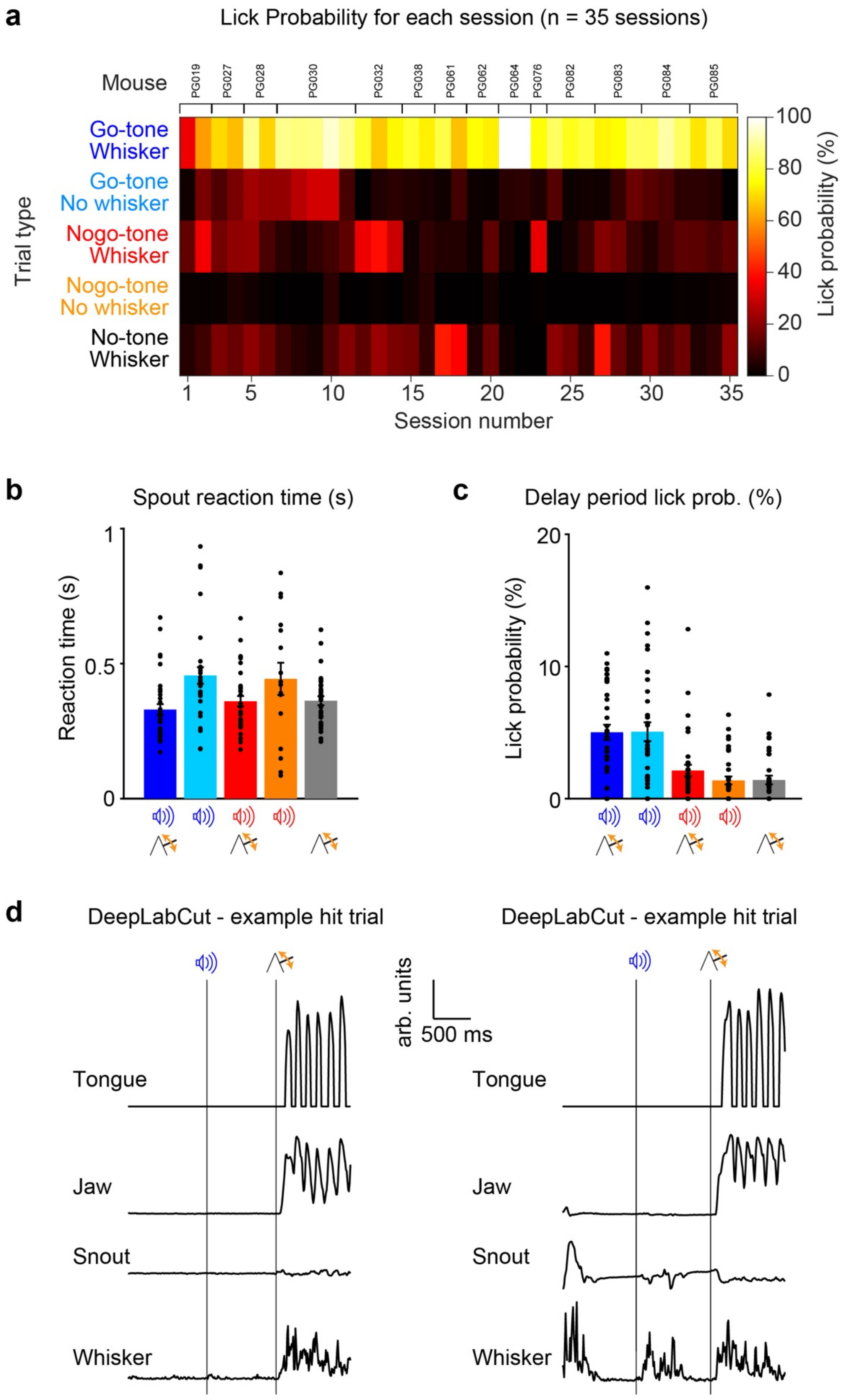

Supplementary Fig. 1 | Behavioral characterization of context-dependent task performance. a, The probability of licking in the reporting window for completed trials

(i.e. not including early lick trials) for each of the 35 behavioral sessions during which electrophysiological measurements of neuronal activity were made. **b**, Mean time from whisker stimulation to tongue-spout contact for the five different trial types for each of the 35 sessions. **c**, The probability of licking as monitored by tongue-spout contacts during the delay period for the five different trial types for each of the 35 sessions. Licking during the delay period caused the abortion of the trial. For **b** and **c**: bars display mean ± SEM; black dots indicate individual sessions. **d**, High-speed video filming was analyzed offline using DeepLabCut to extract tongue, jaw, snout and whisker movements (with y-axis in arbitrary units, arb. units). Both of these example hit trials (Go tone, Whisker stimulus and licking) were included as Quiet trials in our analyses, because both jaw and whisker movements were below threshold during the last 200 ms of the delay period. Source data are provided as a Source Data file.

Supplementary Figure 2

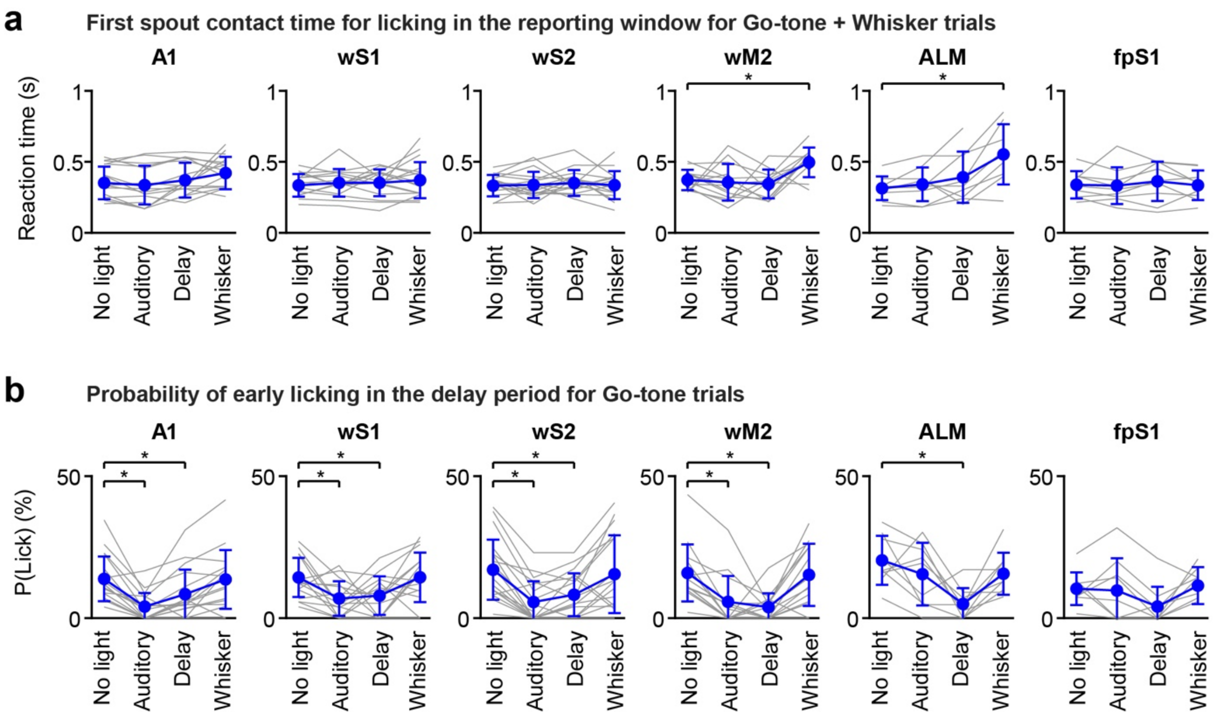

Supplementary Fig. 2 | Behavioral impact of optogenetic inactivation. a, In Go-tone Whisker trials, inactivation of wM2 and ALM in the whisker time period (from 50 ms before the onset of the whisker stimulus to 200 ms after the onset of the whisker stimulus followed by 100 ms ramp down period) significantly delayed reaction time, here quantified as the tongue-spout contact time after the whisker stimulus. Gray lines show individual sessions and plain circles with error bars show mean ± SD. *, p<0.05 No-light vs light trials, two-sided Wilcoxon signed-rank test with FDR correction for each area: A1 n=15-19 sessions; wS1 n=15-20 sessions; wS2 n=17-20 sessions; wM2 n=12-14 sessions, Whisker *P* = 0.03; ALM n= 8-9 sessions, Whisker *P* = 0.02; fpS1 n=9 sessions. **b**, In Go-tone Whisker trials, inactivation of A1, wS1, wS2 and wM2 during the presentation of the auditory Go cue (from 50 ms before the onset of the auditory stimulus to 300 ms after the onset of the auditory stimulus followed by 100 ms ramp down period) decreased the probability of licking in the delay period. Furthermore, inactivation of A1, wS1, wS2, wM2 and ALM during the delay period (from 400 ms after the onset of the auditory stimulus to 850 ms after the onset of the auditory stimulus followed by 100 ms ramp down period) decreased the probability of licking in the delay period. Gray lines show individual sessions and plain circles with error bars show mean ± SD. *, p<0.05 No-light vs light trials, two-sided Wilcoxon signed-rank test with FDR correction for each area: A1 n=17-19 sessions, Auditory *P* = 0.001, Delay P = 0.004; wS1 n=16-20 sessions, Auditory *P* = 0.03, Delay *P* = 0.03; wS2 n=18-20 sessions, Auditory *P* = 0.0009, Delay *P* = 0.0009; wM2 n=14 sessions, Auditory *P* = 0.0005, Delay *P* = 0.0004; ALM n= 9 sessions, Delay *P* = 0.01; fpS1 n=9 sessions. Source data are provided as a Source Data file.

Supplementary Figure 3

a

Go-tone Whisker

	Audio	Delay	Whisker
A1	-39 %	-8 %	-13 %
wS1	-19 %	-16 %	-38 %
wS2	-26 %	-8 %	-35 %
wM2	-33 %	-45 %	-48 %
ALM	-8 %	-32 %	-52 %
fpS1	0 %	-4 %	-9 %

Go-tone

	Audio	Delay	Whisker
A1	-19 %	+1 %	+1 %
wS1	-2 %	+6 %	+1 %
wS2	-16 %	0 %	-8 %
wM2	-10 %	-8 %	-6 %
ALM	-11 %	-6 %	0 %
fpS1	-8 %	-2 %	+4 %

Nogo-tone Whisker

	Audio	Delay	Whisker
A1	+4 %	0 %	-5 %
wS1	+2 %	+2 %	-4 %
wS2	+3 %	+1 %	-7 %
wM2	+4 %	-3 %	-4 %
ALM	+7 %	-5 %	-10 %
fpS1	+8 %	+9 %	-5 %

Nogo-tone

	Audio	Delay	Whisker
A1	-1 %	0 %	0 %
wS1	0 %	-2 %	-1 %
wS2	0 %	-1 %	-1 %
wM2	0 %	0 %	-1 %
ALM	0 %	-1 %	-1 %
fpS1	+2 %	0 %	-1 %

No-tone Whisker

	Audio	Delay	Whisker
A1	-1 %	-5 %	-9 %
wS1	+3 %	-2 %	-6 %
wS2	-1 %	-3 %	-11 %
wM2	+2 %	+1 %	-9 %
ALM	+8 %	0 %	-13 %
fpS1	+5 %	0 %	-6 %

b

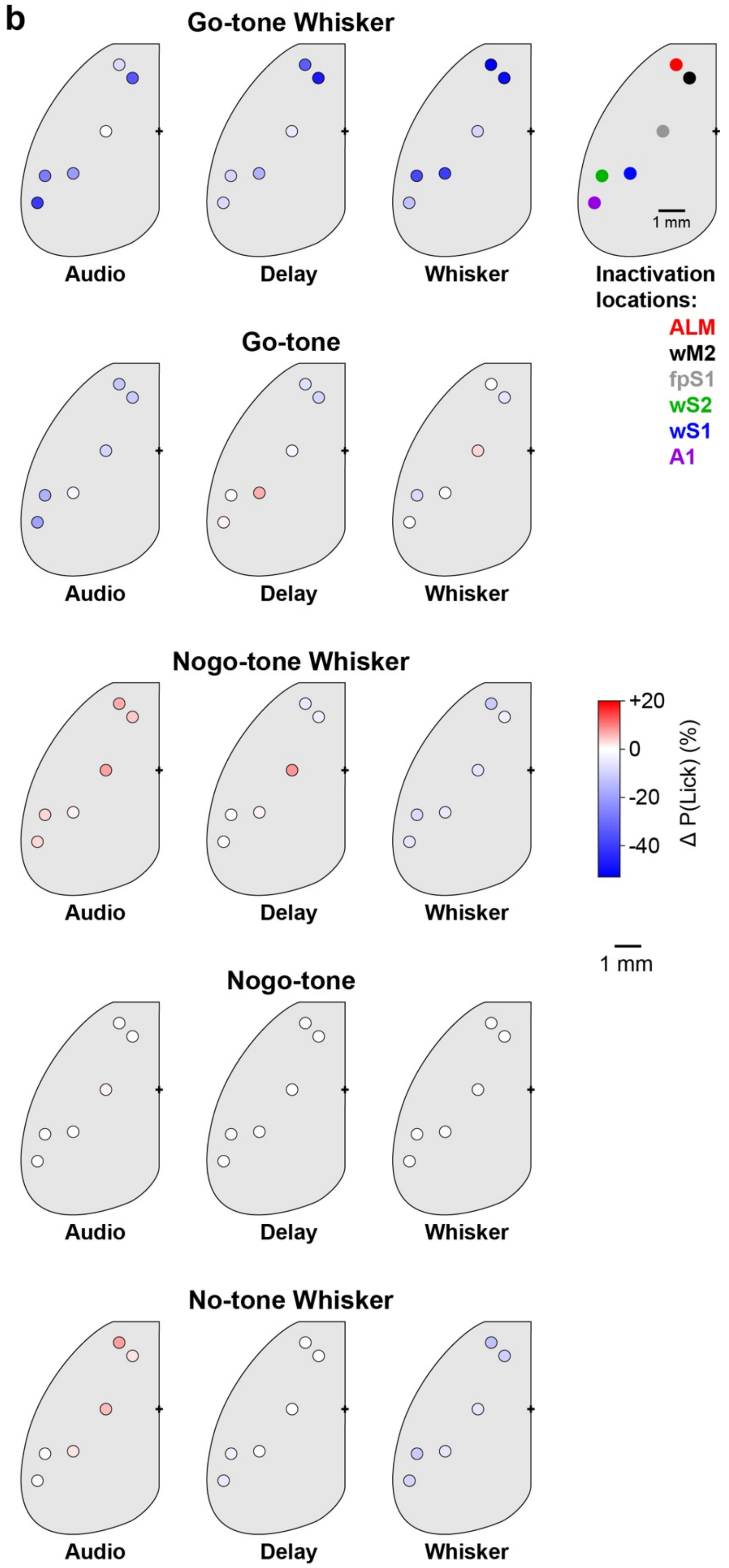

Supplementary Fig. 3 | Impact of optogenetic inhibition across different trial types. a, Each color-coded matrix indicates the change in the probability of licking in

the reporting window relative to no-light trials upon optogenetic inactivation of A1, wS1, wS2, wM2, ALM or fpS1 during the auditory contextual cue (from 50 ms before the onset of the auditory stimulus to 300 ms after the onset of the auditory stimulus followed by 100 ms ramp down period), the delay period (from 400 ms after the onset of the auditory stimulus to 850 ms after the onset of the auditory stimulus followed by 100 ms ramp down period) or the whisker stimulus (from 50 ms before the whisker stimulus to 200 ms after the whisker stimulus followed by 100 ms ramp down period). Important decreases in the probability of licking were found in Go-tone Whisker trials (top, same data as shown in Fig. 2b), but for the other four trial types (Go-tone; Nogo-tone Whisker; Nogo-tone; No-tone Whisker) the effects upon licking probability were less prominent. **b**, Same data as panel a, but shown as a color-coded map of the inactivated locations across the dorsal cortex. Number of sessions: A1, n = 17 sessions; wS1, n = 15-20 sessions; wS2, n = 18 sessions; wM2, n = 14 sessions; ALM, n = 9 sessions; fpS1, n = 9 sessions. Source data are provided as a Source Data file.

Supplementary Figure 4

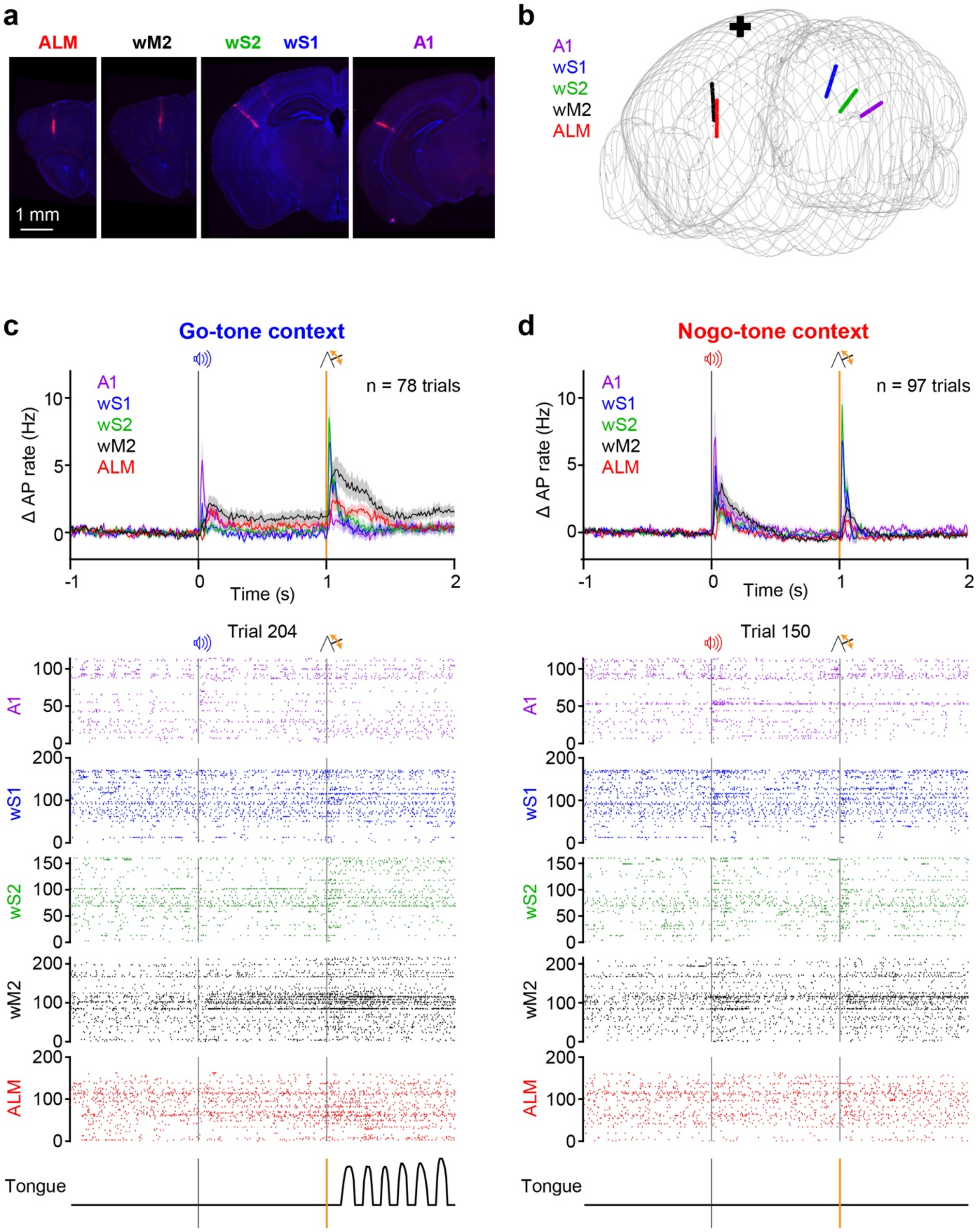

Supplementary Fig. 4 | An example *Neuropixels* recording experiment. a, Five *Neuropixels* probes coated with DiI were acutely lowered into the neocortex of an expert mouse trained in the context-dependent whisker detection task. The probes were targeted to A1, wS1, wS2, wM2, and ALM. After the electrophysiological recordings, the brain was extracted, sliced into 100 μm-thick coronal sections, stained

with DAPI, mounted on slides and imaged with an epifluorescent microscope. Sections with electrode tracks (red) are shown together with the DAPI stain (blue). **b**, The electrode tracks were registered to the Allen Mouse Brain Atlas Common Coordinate Framework (Allen CCF) to give an xyz-coordinate to each unit. **c**, The upper PSTH shows the averaged change in firing rate (Δ AP rate) of all units per cortical area recorded across all Go-tone Whisker Hit trials in this experiment (mean ± SEM). An example Go-tone Whisker Hit trial showing raster plots of spike times (below) for all simultaneously recorded units in the five cortical regions (note some neurons do not fire in this trial). **d**, Same as panel c, but for a Nogo-tone Whisker Correct Rejection trial.

Supplementary Figure 5

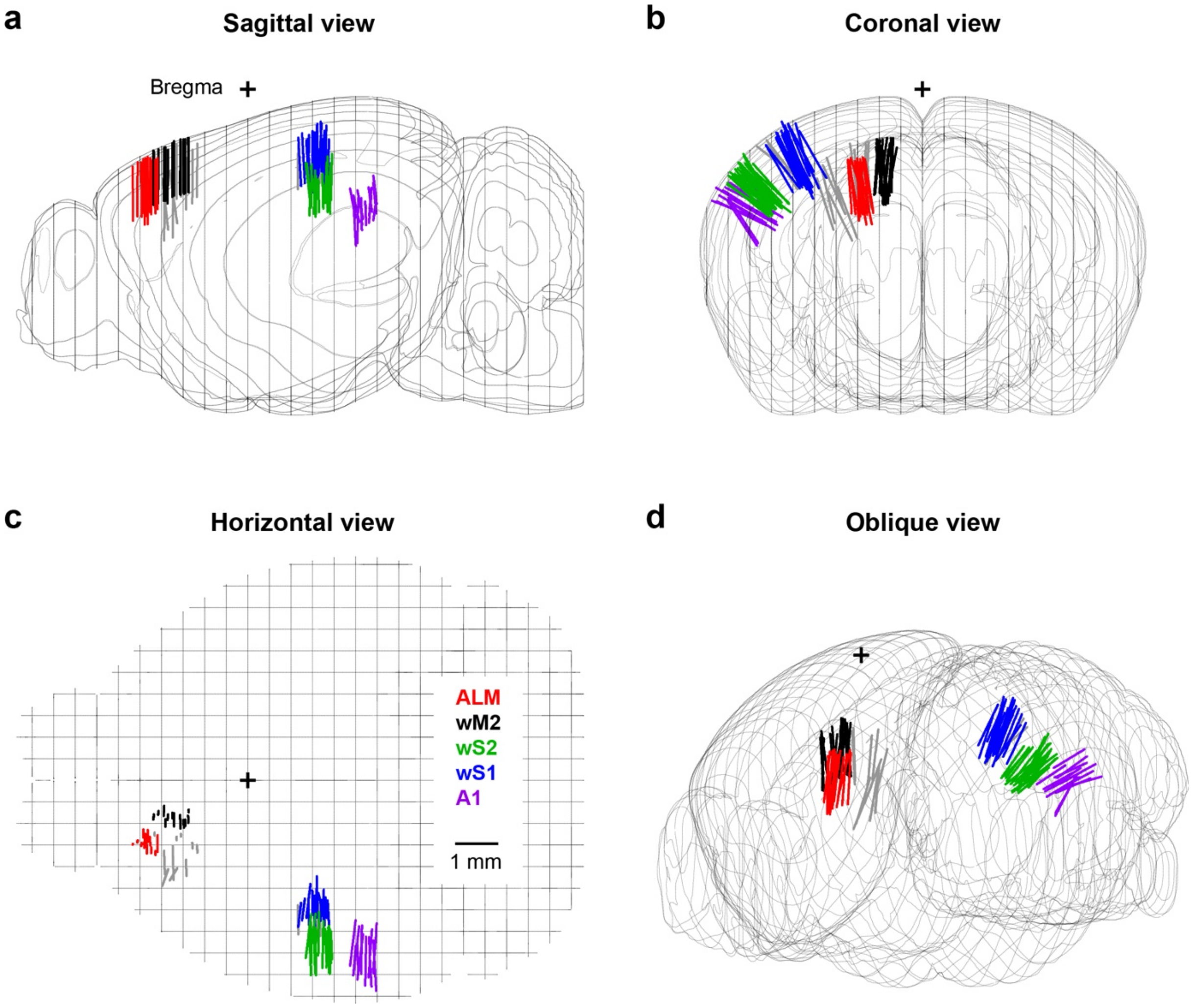

Supplementary Fig. 5 | Probe localization across all experiments. a, The anatomical location of each probe was identified through DiI labelling and registration to the Allen Mouse Brain Atlas Common Coordinate Framework (Allen CCF). Probe locations shown in a sagittal projection were color-coded (red, ALM; black, wM2; green wS2; blue, wS1; purple, A1; and grey, unassigned). **b**, Same as panel a, but for a coronal projection. **c**, Same as panel a, but for a horizontal projection. **d**, Same probe tracks, now shown in a 3D view.

Supplementary Figure 6

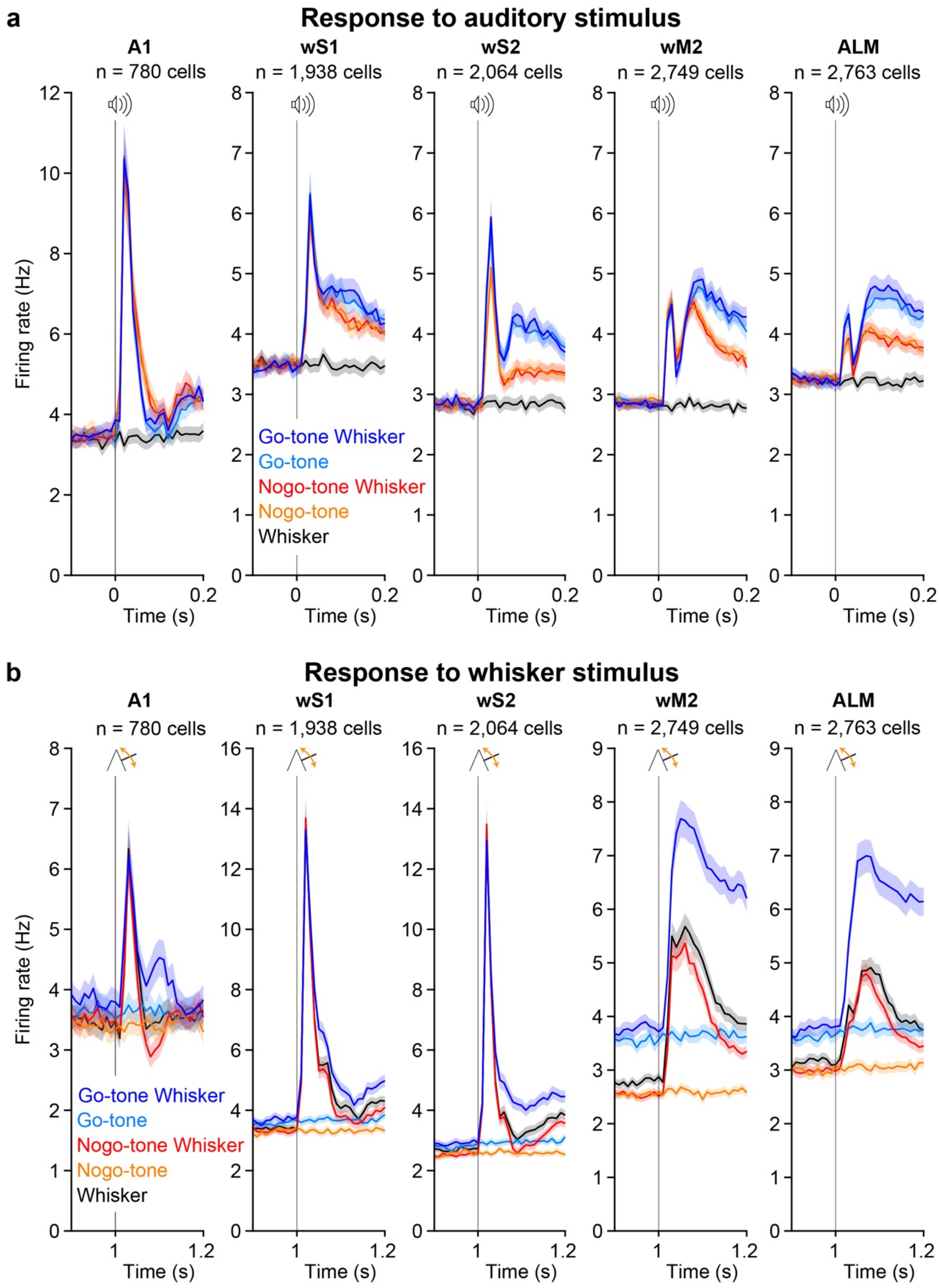

Supplementary Fig. 6 | Action potential firing rate dynamics evoked by the auditory contextual cue and the whisker stimulus. a, Mean ± SEM PSTH with 10 ms time binned histograms relative to the onset time of the auditory contextual cue for different color-coded trial types. **b**, Same as panel a, but relative to the onset of the whisker deflection.

Supplementary Figure 7

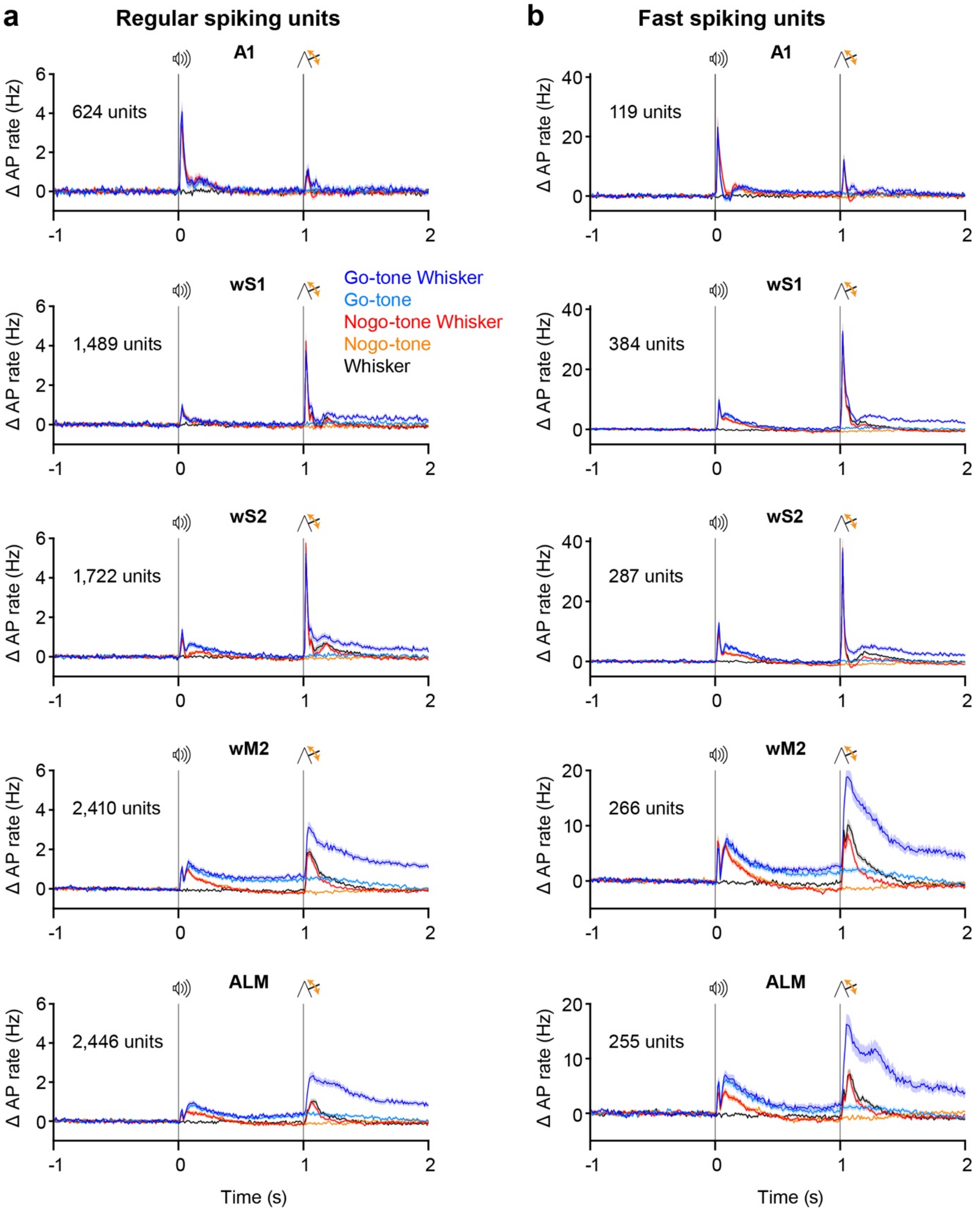

Supplementary Fig. 7 | Firing patterns of Regular Spiking (RS) and Fast Spiking (FS) units. a, Same data as shown in Fig. 3b, but only including RS units (change in AP rate averaged across neurons, mean ± SEM). **b**, Same as a, but for FS units.

Supplementary Figure 8

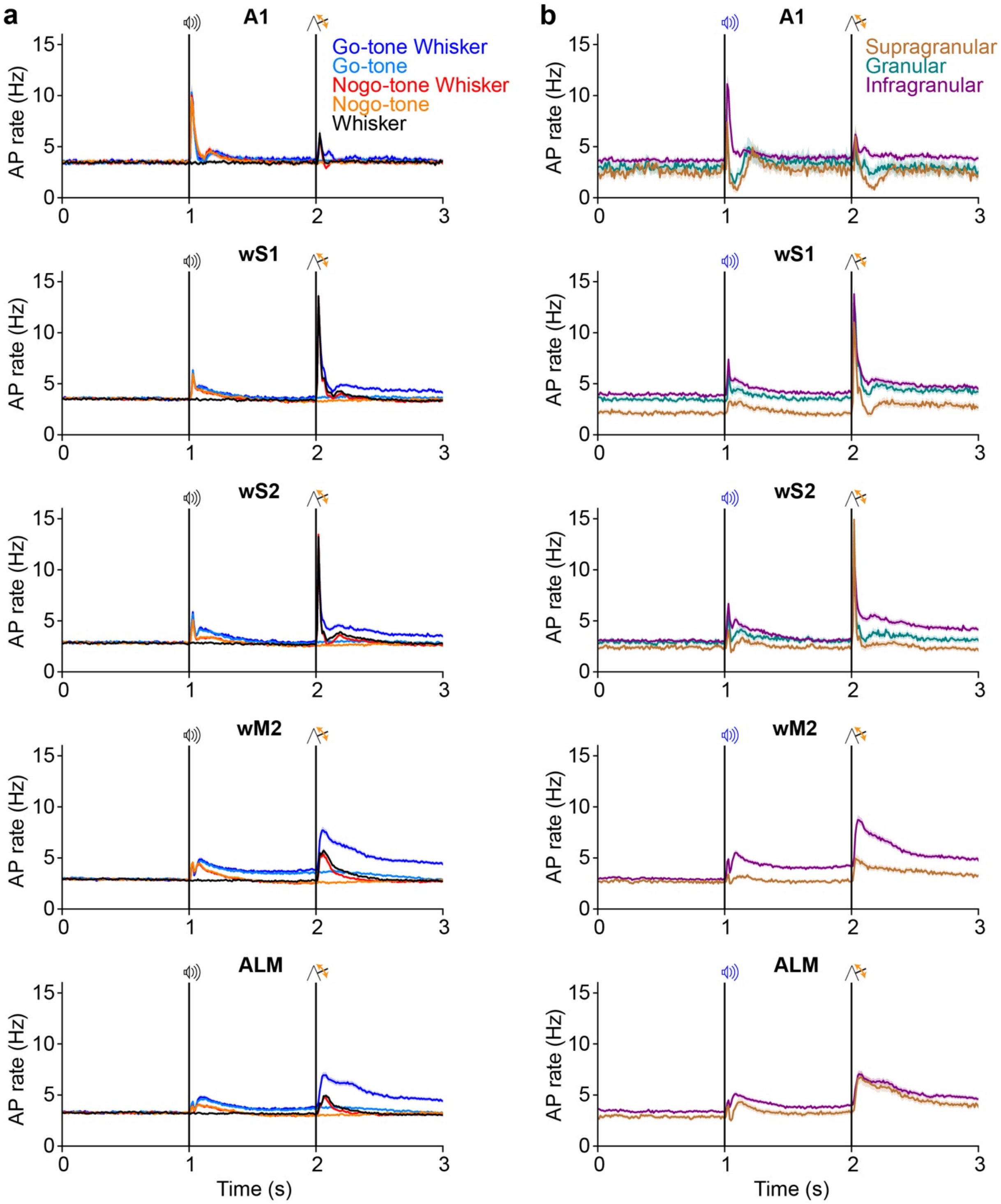

Supplementary Fig. 8 | Neuronal firing rate dynamics across trial types and cortical depth. a, Neuronal firing rate dynamics across trial types for different cortical regions. Same data as shown in Figure 3b, but here without baseline subtraction (AP rate averaged across neurons, mean ± SEM). **b**, Firing rate dynamics for supragranular, granular and infragranular units (mean AP rate averaged across neurons, mean ± SEM).

Supplementary Figure 9

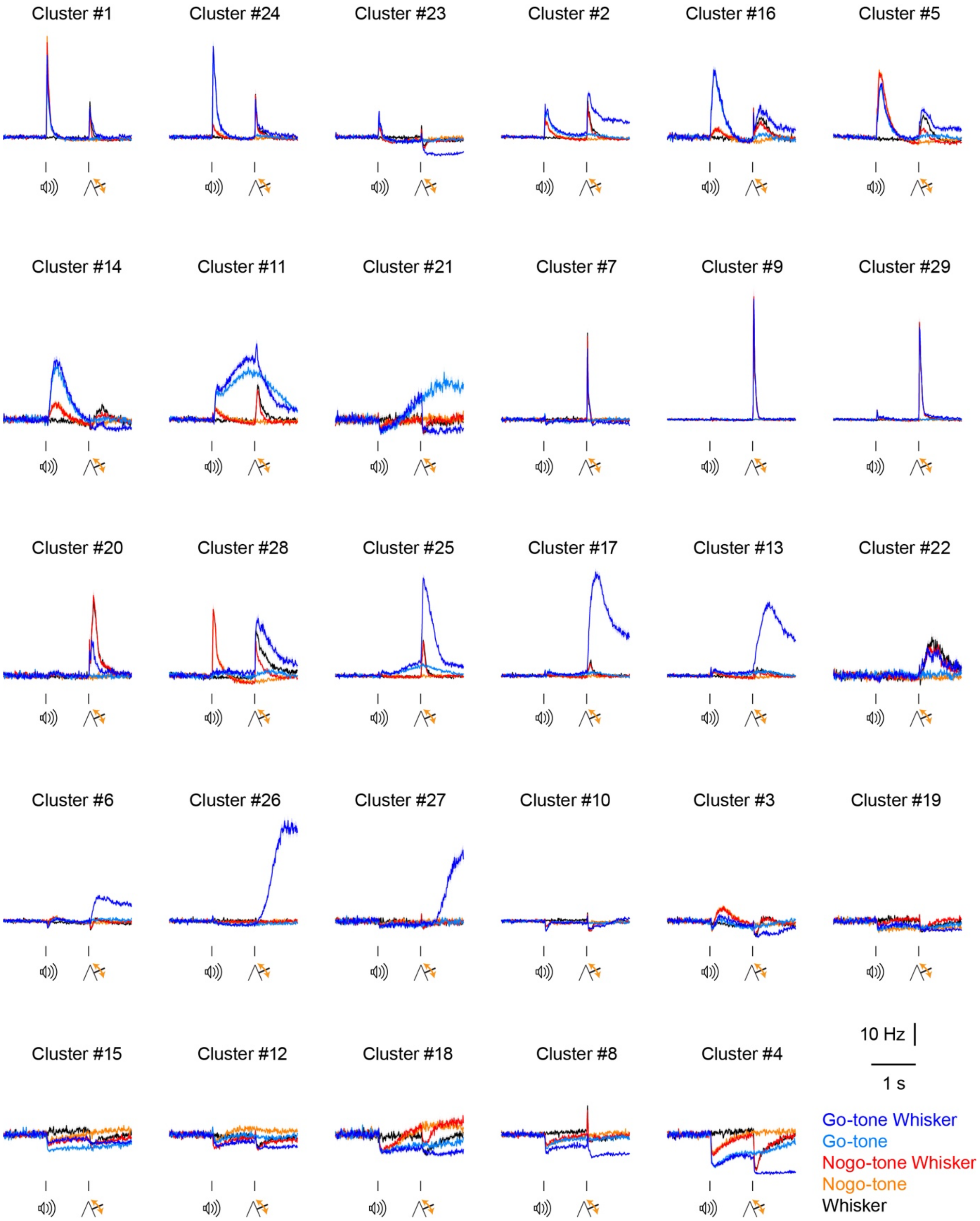

Supplementary Fig. 9 | Firing rates of each cluster. The trial-averaged neuronal activity of each neuron in a cluster was averaged to give the mean firing rate of each cluster identified in the analysis of Fig. 4 (mean ± SEM).

Supplementary Figure 10

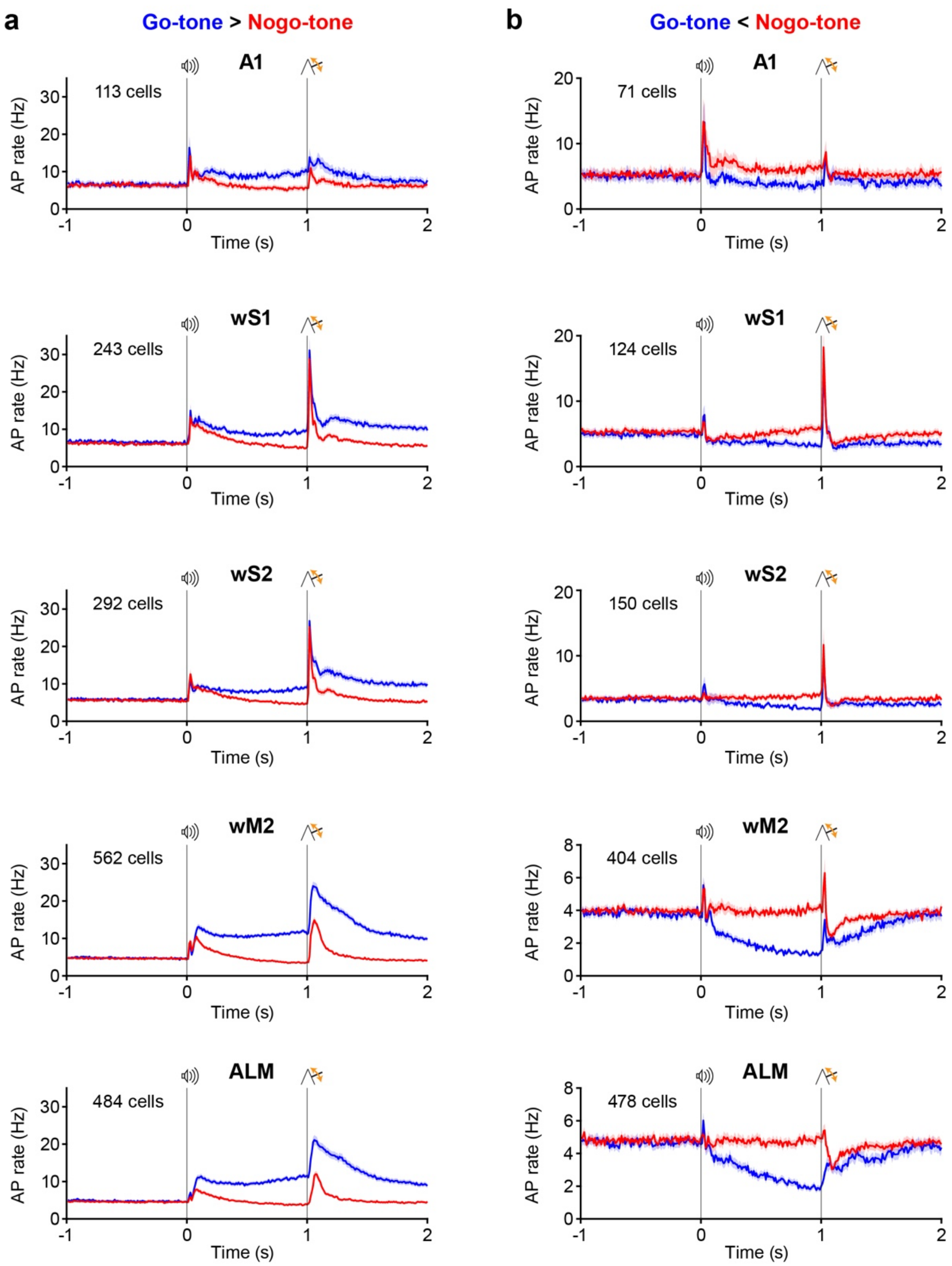

Supplementary Fig. 10 | Context-dependent neuronal activity. a, Neuronal selectivity for Go-tone vs Nogo-tone was computed for the last 200 ms of the delay period by ROC analysis to identify statistically-significantly-modulated neurons, as shown in Fig. 5a. The neurons firing more in Go-tone compared to Nogo-tone trials were averaged for each cortical region recorded (mean ± SEM, blue for Go-tone trials and red for Nogo-tone trials). **b**, Same as panel a, but for neurons showing significantly more firing in Nogo-tone trials compared to Go-tone trials during the last 200 ms of the delay period.

Supplementary Figure 11

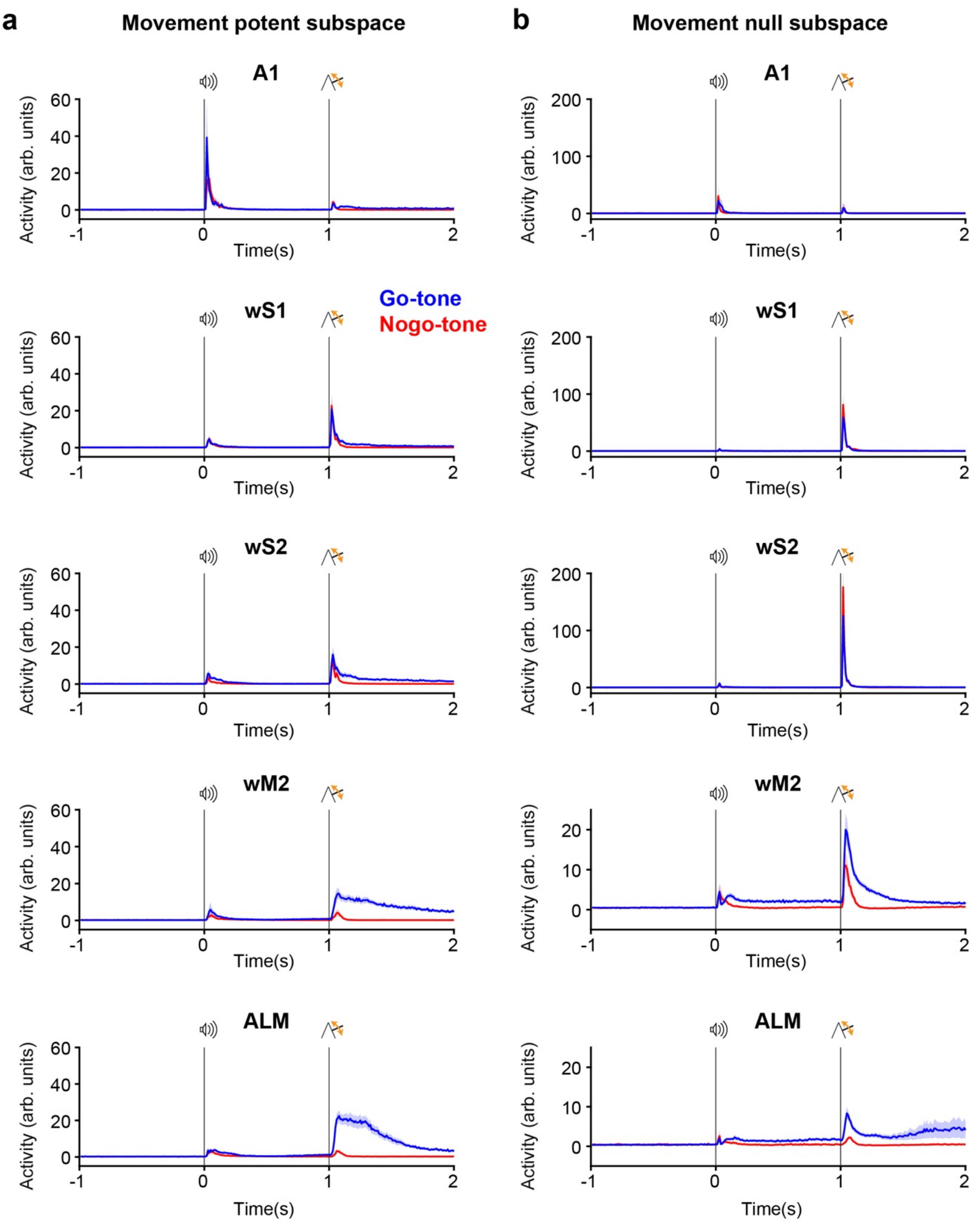

Supplementary Fig. 11 | Neuronal activity projected in 'movement potent' and 'movement null' subspaces. a, Neuronal activity in each recorded area was projected into the 'movement potent' subspace for Go-tone Whisker trials (blue) and Nogo-tone Whisker trials (red) (average across sessions, mean ± SEM). **b**, Same as panel a, but now with neuronal activity projected into the 'movement null' subspace. It is important to note, that context-dependent activity is obvious in the 'movement null' subspace for wM2 and ALM.

Supplementary Figure 12

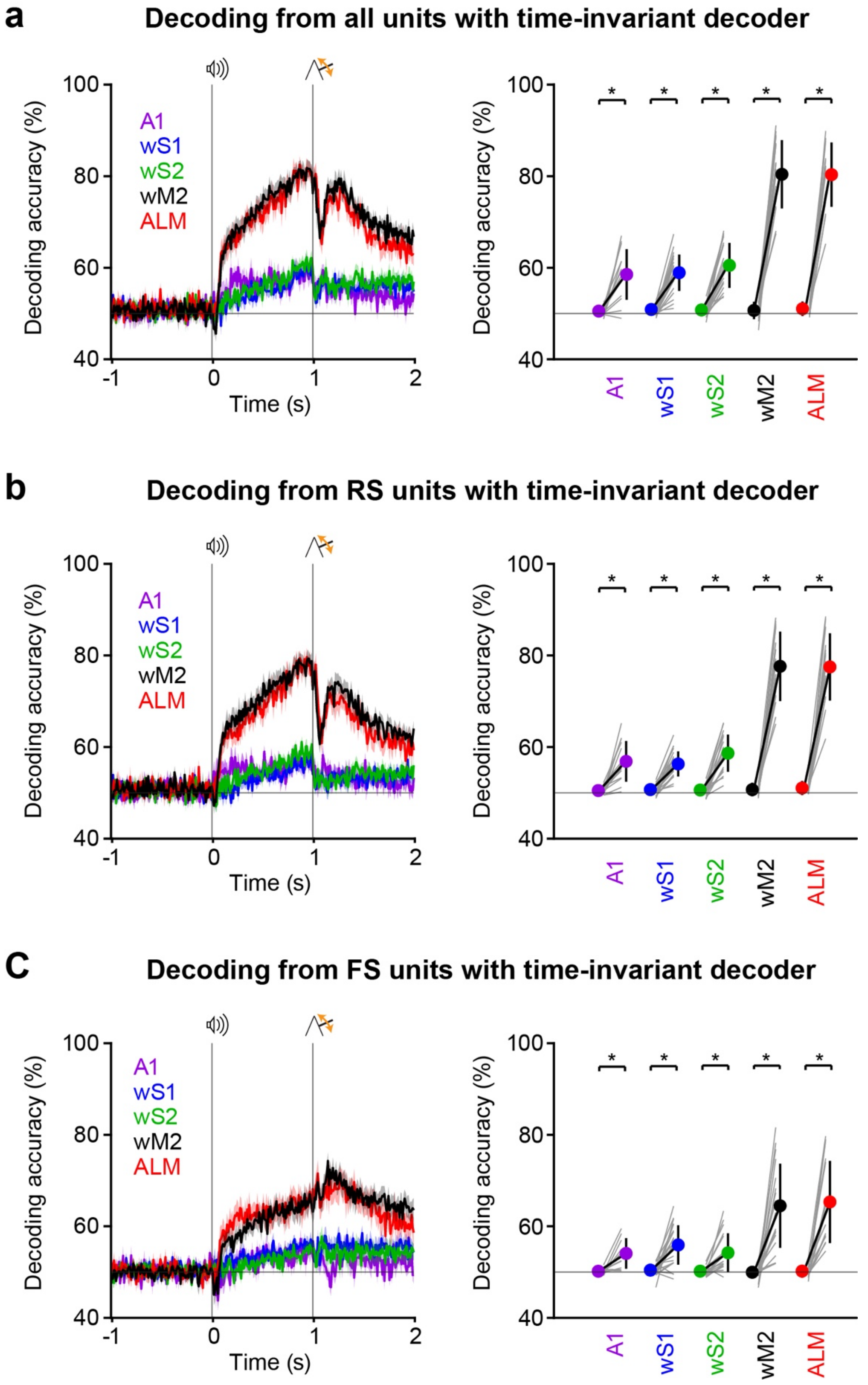

Supplementary Fig. 12 | Context-decoding from Regular Spiking (RS) and Fast Spiking (FS) units. a, The activity of all simultaneously recorded neurons was used to construct a support vector machine (SVM) classifier trained to distinguish correct

Go-tone Whisker trials (Hit trials) from correct Nogo-tone Whisker trials (Correct Reject trials) for each cortical area based on the average activity over the last 200 ms of the delay period. The same decoder was then applied to each 10 ms bin of the neuronal population vector for each area. *Left*, mean accuracy across time for the different areas. Mean ± SEM, average across sessions: A1, n = 14 sessions; wS1, n = 24 sessions; wS2, n = 19 sessions; wM2, n = 18 sessions; ALM, n = 16 sessions. *Right*, quantification of the mean accuracy in the last 200 ms of the delay period. Gray lines show individual sessions; plain circles show mean ± SD. *, $p < 0.05$, accuracy during delay vs baseline, two-sided Wilcoxon signed-rank test: A1 $P = 0.0004$, wS1 $P = 2\text{x}10^{-5}$, wS2 $P = 0.0001$, wM2 $P = 0.0002$, ALM $P = 0.0004$. **b**, Same as panel a, but now constructing the SVM decoder based solely on the activity of RS units. Mean ± SEM, average across sessions: A1, n = 14 sessions; wS1, n = 24 sessions; wS2, n = 19 sessions; wM2, n = 18 sessions; ALM, n = 16 sessions. *Right*, quantification of the mean accuracy in the last 200 ms of the delay period. Gray lines show individual sessions; plain circles show mean ± SD. *, $p < 0.05$, accuracy during delay vs baseline, two-sided Wilcoxon signed-rank test: A1 $P = 0.0001$, wS1 $P = 2\text{x}10^{-5}$, wS2 $P = 0.0001$, wM2 $P = 0.0002$, ALM $P = 0.0004$. **c**, Same as panel a, but for FS units only. Mean ± SEM, average across sessions: A1, n = 10 sessions; wS1, n = 23 sessions; wS2, n = 18 sessions; wM2, n = 18 sessions; ALM, n = 16 sessions. *Right*, quantification of the mean accuracy in the last 200 ms of the delay period. Gray lines show individual sessions; plain circles show mean ± SD. *, $p < 0.05$, accuracy during delay vs baseline, two-sided Wilcoxon signed-rank test: A1 $P = 0.01$, wS1 $P = 0.0001$, wS2 $P = 0.0006$, wM2 $P = 0.0002$, ALM $P = 0.0004$. Note that both RS and FS neurons encode contextual information during the delay period most prominently in wM2 and ALM. After the whisker stimulus, interestingly, RS units show decreased context-decoding, whereas FS units increased context-decoding. Source data are provided as a Source Data file.

Supplementary Figure 13

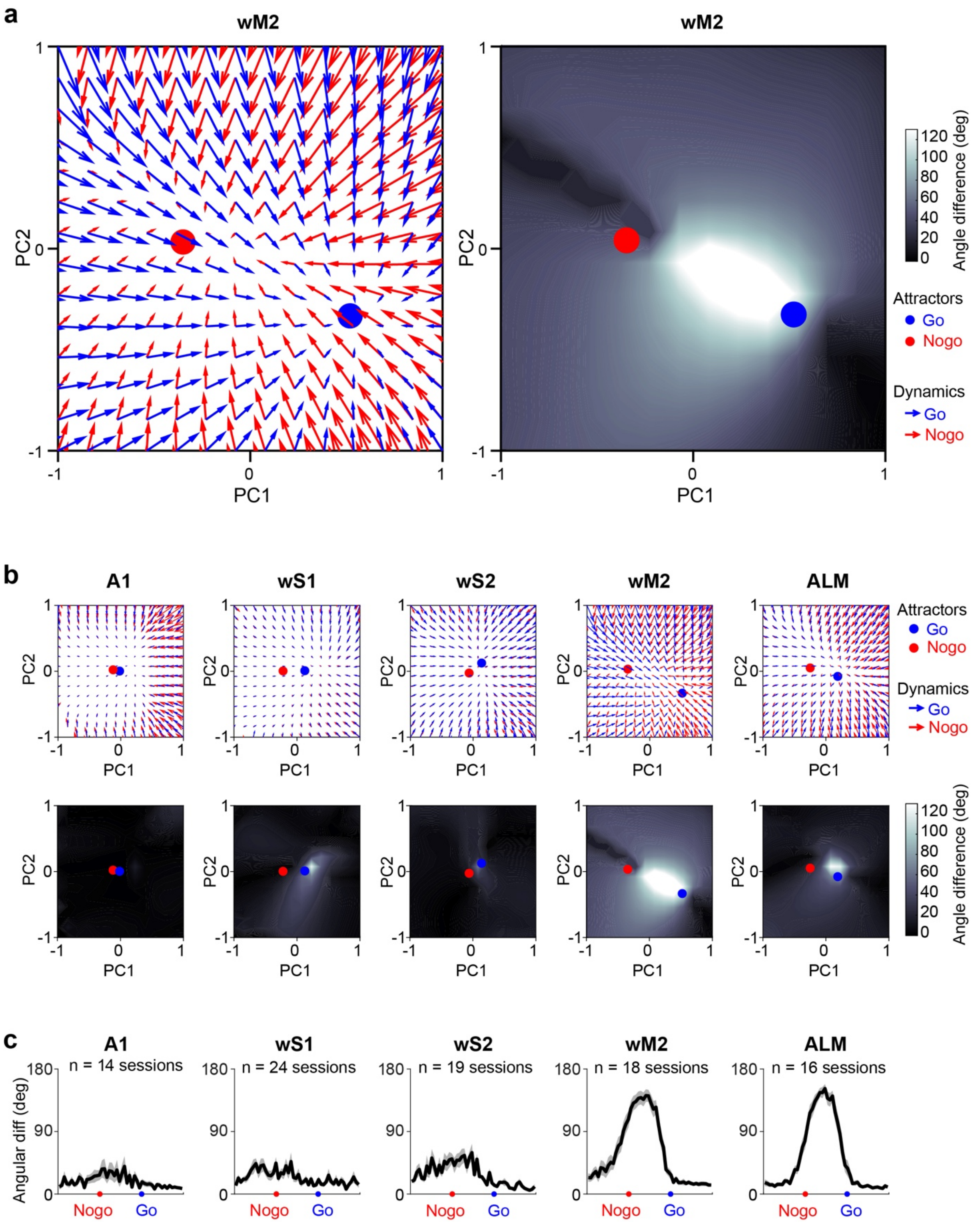

Supplementary Fig. 13 | Attractor fields for stable context representations. a, Neuronal activity in wM2 from a single example session was projected into a PCA space defined across correctly-executed GoTone-Whisker-Lick and NogoTone-Whisker-NoLick trials. *Left panel* - The arrows originate from a given neuronal population state vector at time 't' and point towards the average change in PC space

in the next 10 ms bin of neuronal activity at ‘t + 10 ms’ computed across the late delay period (0.8 – 1 s after the auditory cue). Two distinct attractor-like states are apparent for Go (blue circles) vs Nogo trials (red circles). *Right panel* - Grey-scale representation of the angular difference in the PC gradients comparing GoTone and NogoTone trials. This metric helps quantify the degree of separation of GoTone and NogoTone in a single metric. **b**, Similar analyses as shown in panel a, but now shown also for A1, wS1, wS2 and ALM from the same recording session. **c**, To compare across experiments, we normalized the distance separating the stable Go vs Nogo fixed points, and computed the average angular difference along that dimension across the Hit vs Correct Rejection trials.

Supplementary Figure 14

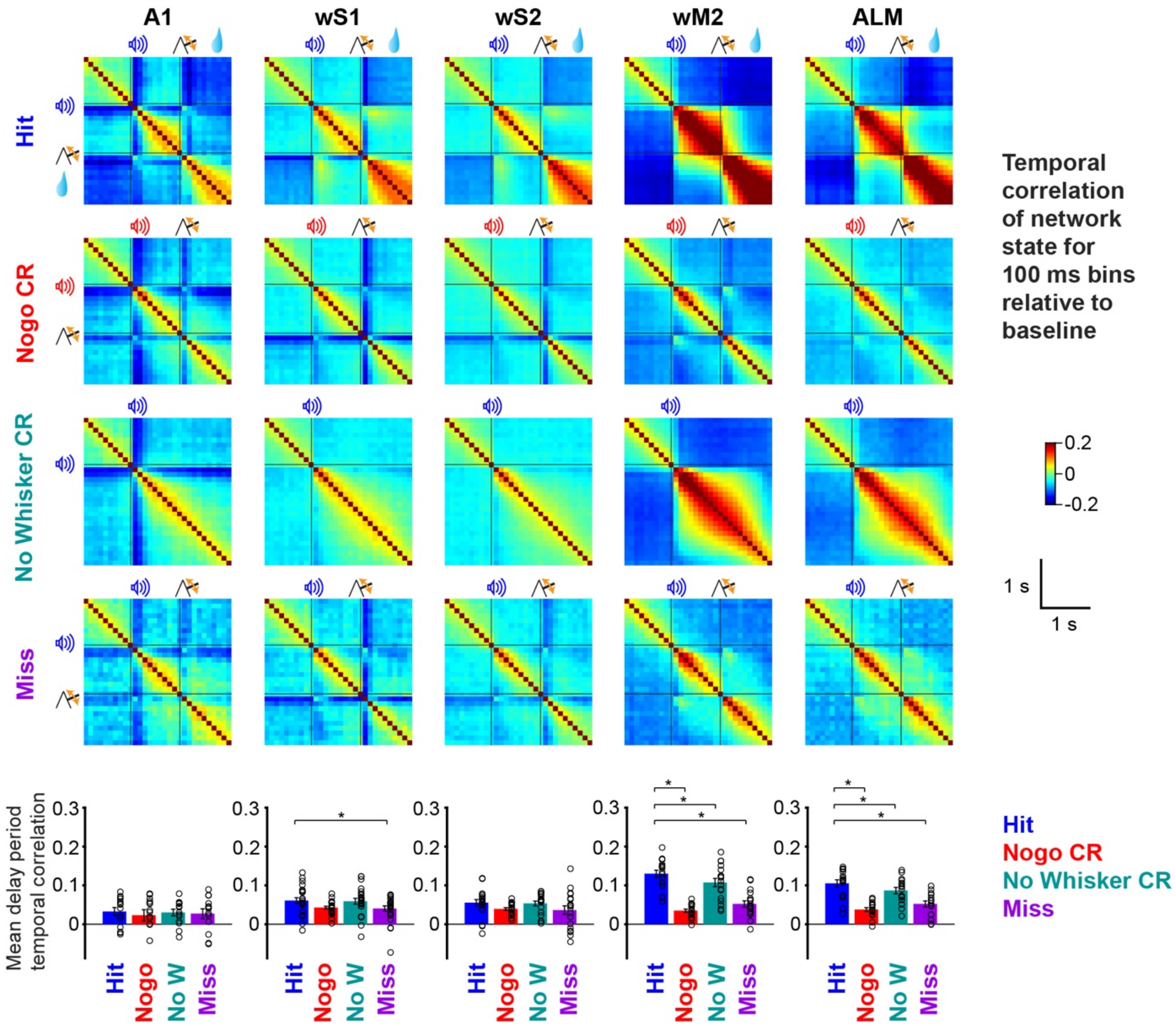

Supplementary Fig. 14 | Temporal correlation of neuronal activity at 100 ms resolution. Same analysis as Fig. 6c, but instead of correlating the population activity in single trials across 10 ms bins, here we use 100 ms bins, obtaining higher mean values of correlation, but otherwise finding similar dynamics. Bars with error bars show mean ± SEM; black circles show individual sessions. *, $p < 0.05$, Hit vs other trial types, two-sided Wilcoxon signed-rank test with Bonferroni correction for each area: A1 n = 14 sessions; wS1 n = 24 sessions, Hit vs Miss $P = 0.03$; wS2 n = 19 sessions; wM2 n = 18 sessions, Hit vs Nogo $P = 0.0006$, Hit vs NoW $P = 0.003$, Hit vs Miss $P = 0.0009$; ALM n = 16 sessions, Hit vs Nogo $P = 0.002$, Hit vs NoW $P = 0.004$, Hit vs Miss $P = 0.002$. Source data are provided as a Source Data file.

Supplementary Figure 15

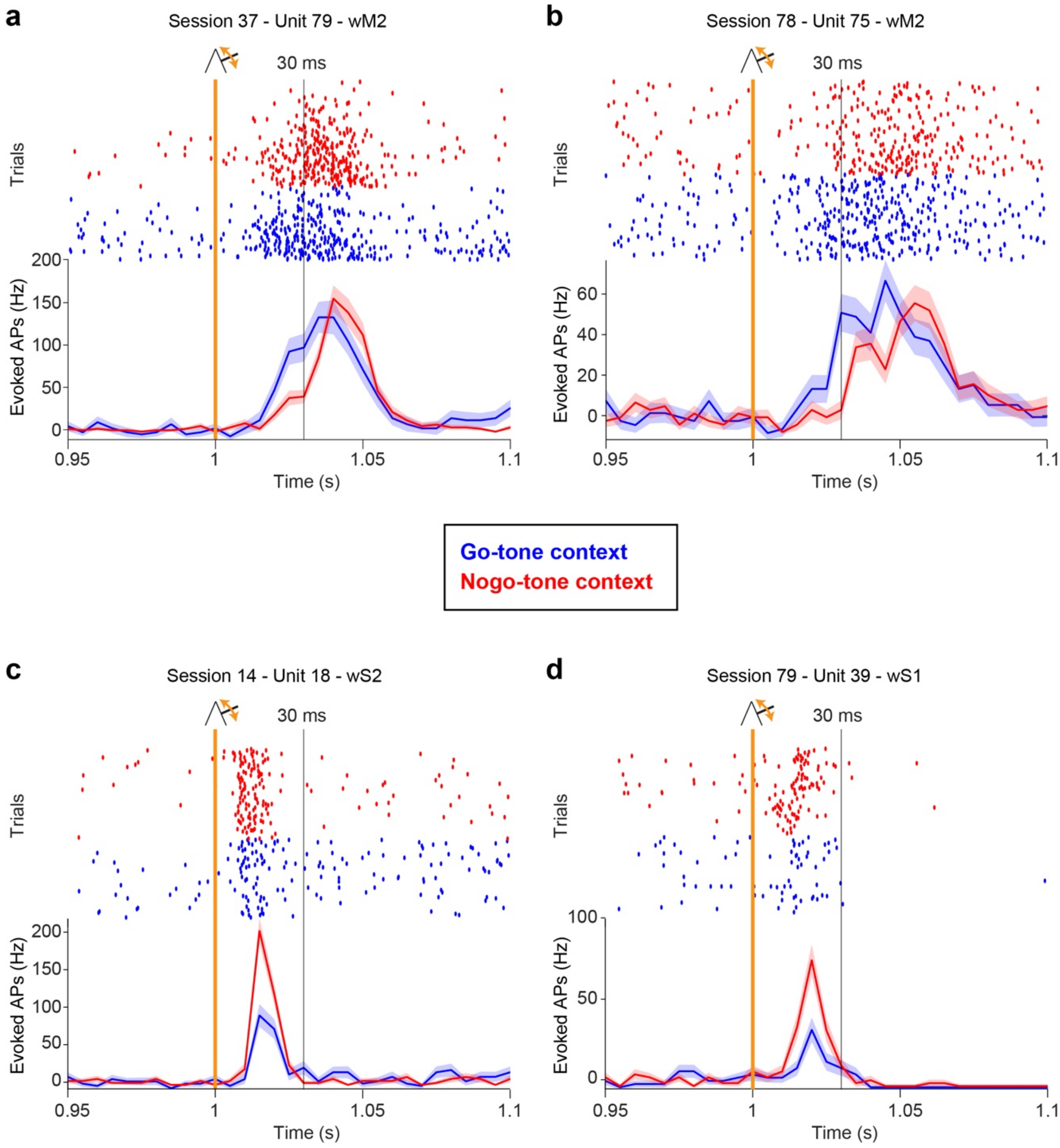

Supplementary Fig. 15 | Example neurons showing context-dependent whisker deflection evoked sensory responses. a, Spike rasters (above) show action potential firing times of an example unit in wM2 in the period immediately surrounding whisker deflection at time 1 s, with Go-tone trials shown in blue and Nogo-tone trials shown in red. The baseline-subtracted, trial-type averaged firing rate is shown below for the example neuron (mean ± SEM), which has a faster and larger sensory-evoked response in the Go-tone context. **b**, Same as panel a, but for another example neuron in wM2, also showing a preference for whisker responsiveness in the Go-tone context. **c**, Same as panel a, but for an example neuron in wS2, showing a higher whisker response in Nogo-tone trials. **d**, Same as panel a, but for a neuron in wS1, also firing preferentially to the whisker stimulus in Nogo-tone trials.

Supplementary Figure 16

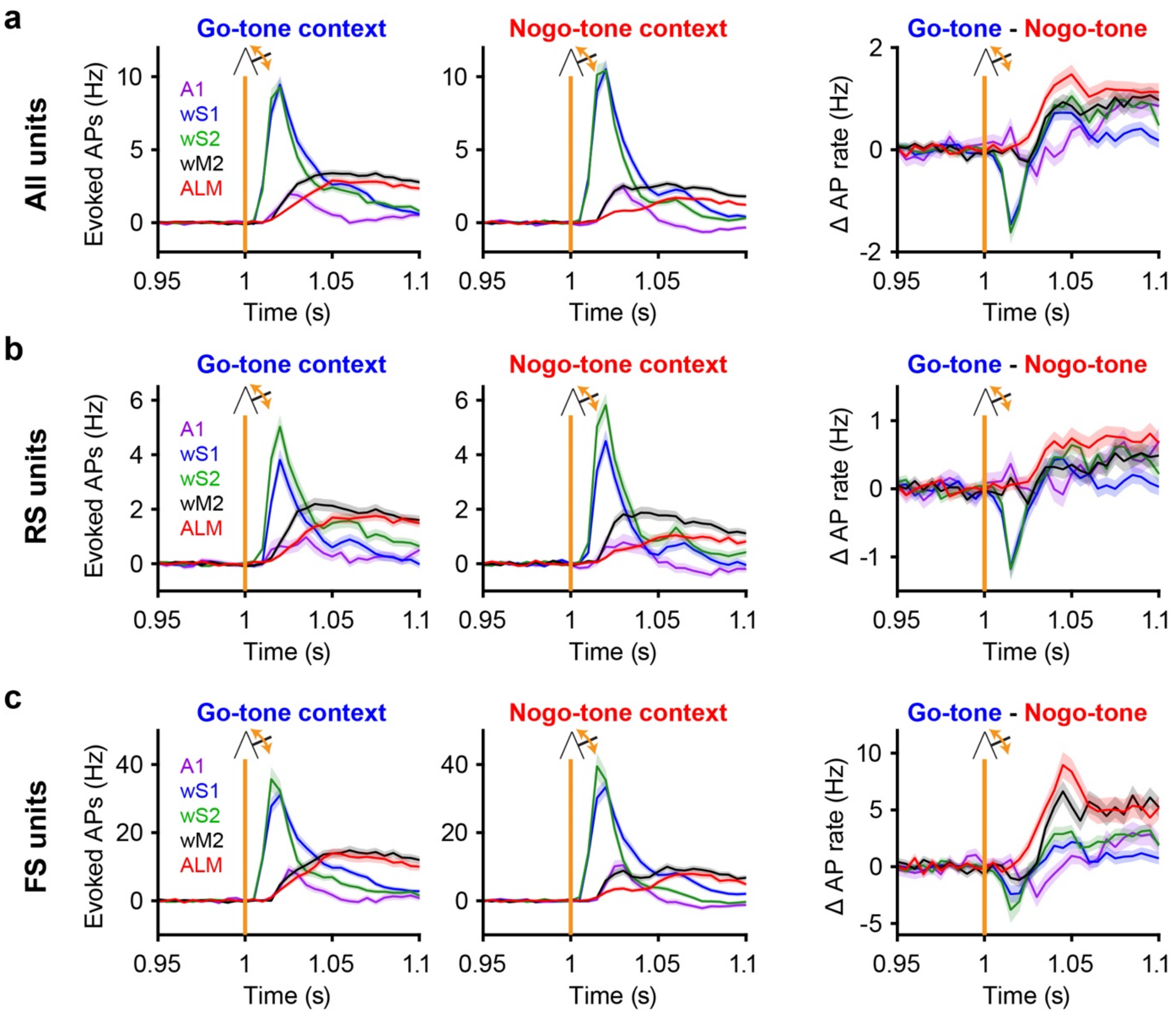

Supplementary Fig. 16 | Context-dependent whisker sensory processing in Regular Spiking (RS) and Fast Spiking (FS) units. a, The baseline-subtracted neuronal firing rates of all units in the recorded cortical regions in the time period surrounding the whisker deflection at time 1 s in the Go-tone context (*left*), Nogo-tone context (*center*) and the difference (*right*, Go-tone minus Nogo-tone) (Δ AP, average across neurons, mean ± SEM,). Same data as Fig. 7a&b. **b**, Same as panel a, but now including only RS units. **c**, Same as a, but now only for FS units. Note that although the context differences in the sensory-evoked response are qualitatively similar in RS and FS units, there are also some apparent differences. The Go-tone-reduced evoked activity in wS1 and wS2 at very early time points is more prominent in RS units, whereas the slightly-delayed Go-tone-increased sensory-evoked response in wM2 and ALM appears more prominent in FS neurons.

Supplementary Figure 17

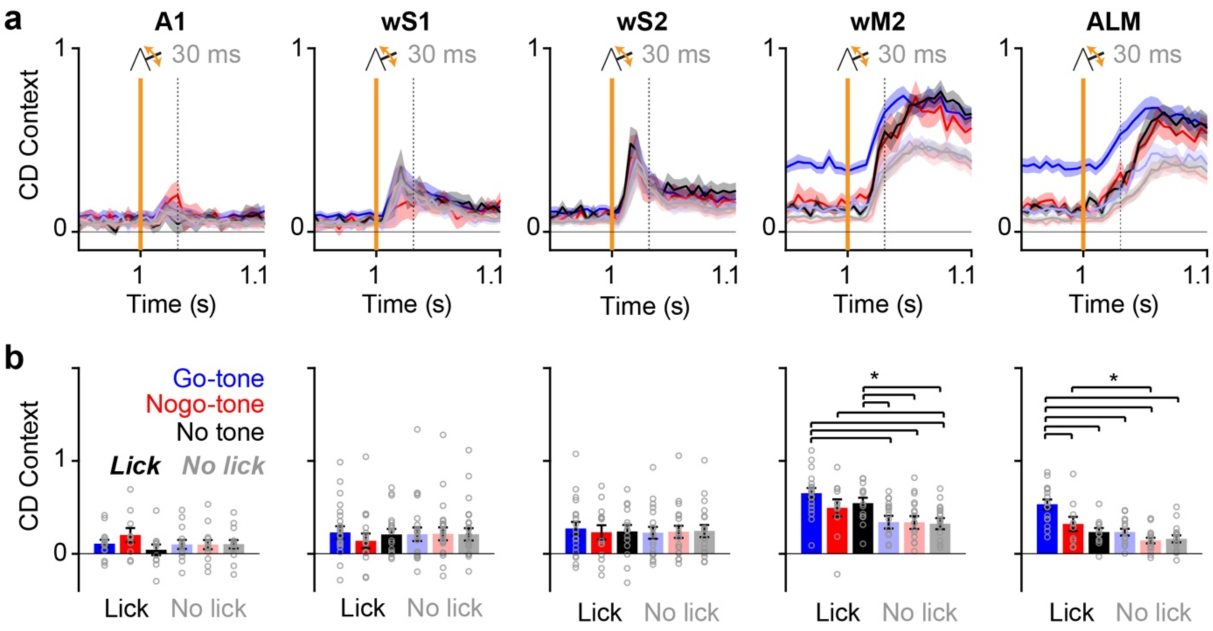

Supplementary Fig. 17 | Quantification of neuronal activity in the context-coding direction for lick and no lick trials in different contexts. a, The time-course of neuronal activity projected in the context coding direction (CD Context) for trials including a whisker deflection preceded by a Go-tone (blue), Nogo-tone (red) or no tone (black) (average across sessions, mean ± SEM). Trials in which licking was later initiated during the reporting period are in bright colors, whereas trials in which the mice did not lick are in pale colors. **b,** At 30 ms after the whisker deflection, Go-tone trials with licking and Nogo-tone trials with licking have significantly more activity in the wM2 context coding direction compared to no lick trials. Bars with error bars show mean ± SEM and grey circles show individual sessions. *, $p < 0.05$, Kruskal-Wallis test, followed by LSD-corrected post hoc paired comparisons: A1 n = 10-14 sessions; wS1 n = 18-24 sessions; wS2 n = 14-19 sessions; wM2 n = 11-18 sessions, Kruskal-Wallis test P = 0.0004, paired comparisons Go-tone Lick vs No lick trials P = 0.0009, P = 0.0005 and P = 0.0002, Nogo-tone Lick vs No tone no lick trials P = 0.04, No-tone Lick vs No lick trials P = 0.049, P = 0.04 and P = 0.02; ALM n = 10-16 sessions, Kruskal-Wallis test $P = 2x10^{-5}$, paired comparisons Go-tone Lick vs Nogo-tone Lick P = 0.03, Go-tone Lick vs No tone Lick P = 0.004, Go-tone Lick vs Go-tone No lick P = 0.0009, Go-tone Lick vs Nogo-tone No lick $P = 1x10^{-6}$, Go-tone Lick vs No tone No lick $P = 6x10^{-6}$, Nogo-tone Lick vs Nogo-tone No lick, P = 0.04. Source data are provided as a Source Data file.

Supplementary Figure 18

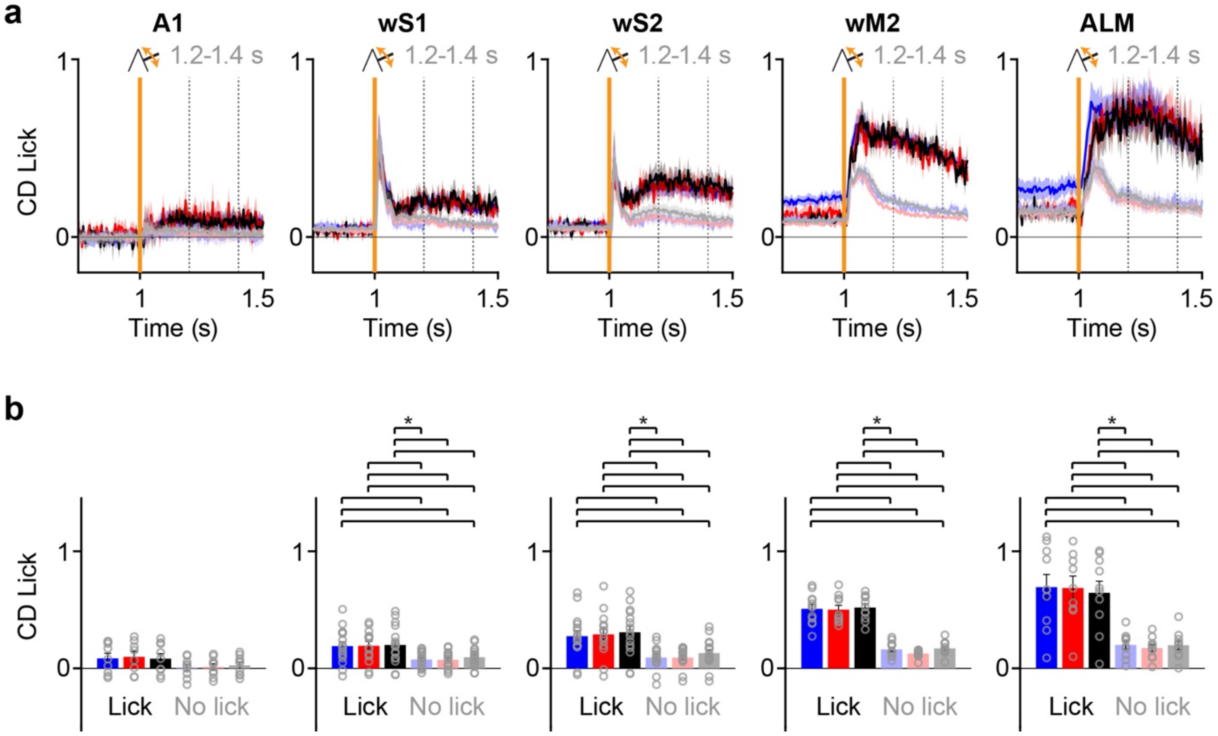

Supplementary Fig. 18 | Analysis of neuronal activity in the lick coding direction during licking. a, Same analysis as shown in Figure 8b, but now examining a longer and later time window after whisker stimulation. Increased neuronal activity in the lick coding direction (CD Lick) appears to be present in the time 0.2 to 0.4 s after the whisker stimulus (timing indicated by vertical dotted lines) (average across sessions, mean ± SEM). **b**, Statistical analyses across recording sessions reveals significant differences in the time 0.2 to 0.4 s after the whisker stimulus comparing lick trials (for both correct and false alarm trials) and no lick trials (for both miss and correct rejection trials) in wS1, wS2, wM2 and ALM. The projection amplitude in the lick coding direction was largest for ALM, followed by wM2, wS2 and wS1 (in rank order), with A1 having the smallest and not significant difference. Bars with error bars show mean ± SEM and grey circles show individual sessions. *, $p < 0.05$, Kruskal-Wallis test, followed by LSD-corrected post hoc paired comparisons: A1 n = 8-9 sessions; wS1 n = 14-16 sessions, Kruskal-Wallis test P = 0.004, paired comparisons Go-tone Lick vs No lick trials P = 0.01, P = 0.01 and P = 0.04, Nogo-tone Lick vs No lick trials P = 0.01, P = 0.01 and P = 0.04, No tone Lick vs No lick trials P = 0.01, P = 0.01 and P = 0.045; wS2 n = 14-16 sessions, Kruskal-Wallis test $P = 7 \times 10^{-6}$, paired comparisons Go-tone Lick vs No lick trials P = 0.0006, P = 0.0005 and P = 0.01, Nogo-tone Lick vs No lick trials P = 0.0006, P = 0.0006 and P = 0.01, No tone Lick vs No lick trials P = 0.0004, P = 0.0003 and P = 0.007; wM2 n = 10-12 sessions, Kruskal-Wallis test $P = 5 \times 10^{-10}$, paired comparisons Go-tone Lick vs No lick trials $P = 4 \times 10^{-5}$, $P = 2 \times 10^{-6}$ and P = 0.0004, Nogo-tone Lick vs

No lick trials P = 0.0001, P = 6×10^{-6} and P = 0.0008, No-tone Lick vs No lick trials P = 3×10^{-5}, P = 1×10^{-6} and P = 0.0002; ALM n = 9-10 sessions, Kruskal-Wallis test P = 4×10^{-5}, paired comparisons Go-tone Lick vs No lick trials P = 0.002, P = 0.0007 and P = 0.002, Nogo-tone Lick vs No lick trials P = 0.004, P = 0.001 and P = 0.004, No-tone Lick vs No lick trials P = 0.005, P = 0.002 and P = 0.005. Source data are provided as a Source Data file.

Supplementary Figure 19

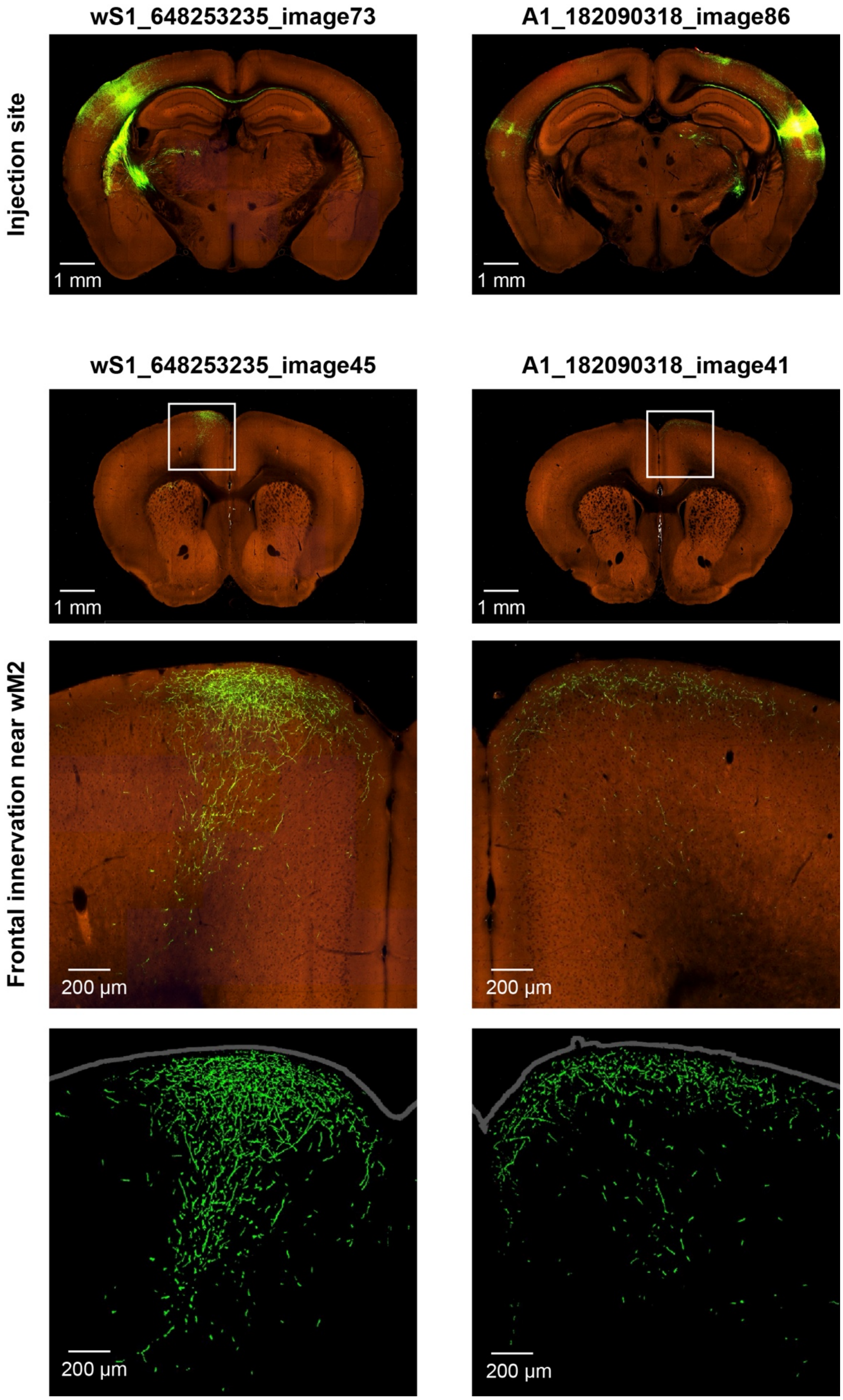

Supplementary Fig. 19 | Axonal projections from wS1 and A1 to wM2. We selected two experiments from the Allen Mouse Brain Connectivity database

(https://connectivity.brain-map.org) to highlight the convergence of axonal projections from wS1[54] and A1[49] in wM2. The left column shows images from experiment 648253235 in which a Cre-dependent adenoassociated virus (AAV) encoding GFP was injected into wS1 (also termed SSp-bfd) of a Rbp4-Cre_KL100 transgenic mouse expressing Cre-recombinase preferentially in layer 5 neurons (https://connectivity.brain-map.org/transgenic/experiment/648253235). The right column shows images from experiment 182090318 in which an AAV encoding Cre-dependent GFP was injected into A1 (also termed AUDp) of a Rbp4-Cre_KL100 mouse (https://connectivity.brain-map.org/transgenic/experiment/182090318). The upper row shows coronal images of sensory cortex, including the injection sites in wS1 (left) and A1 (right). GFP fluorescence is shown in green and autofluorescence is shown in red. The second row shows coronal images of frontal cortex including wM2. A white box highlights the region of wM2, which is shown at higher resolution in the third row of images. The bottom row is the same as above, except showing only the GFP channel. Axons from both wS1 and A1 appear to innervate wM2.
