# Supplementary material for: Contextual gating of whisker-evoked responses by frontal cortex supports flexible decision making
Source: Nat Commun. 2026 May 26;17:5982. doi: 10.1038/s41467-026-73622-y (PMC13346651; doi:10.1038/s41467-026-73622-y)
Supplement: Supplementary file 3 — Transparent Peer Review file [file 41467_2026_73622_MOESM3_ESM.pdf]

nature portfolio

Peer Review File

Contextual gating of whisker-evoked responses by frontal cortex supports flexible decision making

Corresponding Author: Professor Carl Petersen

Parts of this Peer Review File have been redacted as indicated to remove third-party material.

This file contains all reviewer reports in order by version, followed by all author rebuttals in order by version.

Version 0:

Reviewer comments:

Reviewer #1

(Remarks to the Author)
This manuscript by Ghaderi, Crochet, and Petersen presents a comprehensive and technically impressive investigation into how frontal cortex—particularly the whisker-related secondary motor cortex (wM2)—integrates contextual auditory information to gate somatosensory processing and guide flexible decision making in mice. The authors employ a novel behavioral paradigm requiring mice to use a brief auditory cue (Go or Nogo) to interpret an upcoming identical whisker stimulus and decide whether to lick for reward. The paper follows the tradition of the lab to expertly characterize the progression of activity over multiple cortical areas during sensory processing and task execution.

Combining precise spatiotemporal optogenetic inactivation with large-scale Neuropixels recordings, the authors delineate the contributions of distinct cortical areas across auditory, delay, and sensory epochs. Their results show that wM2 encodes context during the delay period through persistent activity, and that wM2 activity within 30 ms of whisker stimulation predicts the behavioral outcome, even in error trials. The findings position wM2 as a critical hub for context-dependent sensory-motor transformation.

The study provides significant conceptual advances by identifying wM2 as the site of context-sensory integration in a whisker-based decision-making task. While prior work has implicated ALM and broader frontal cortex in similar tasks, this manuscript clearly delineates the sequential and causal roles of wM2 and ALM. These findings are novel and compelling and the conclusions are well-supported, and the work will be of wide interest.

I have the following suggestions to further improve the manuscript before publication.
While the study makes a compelling case for wM2's role, it could benefit from a deeper discussion of how wM2 integrates upstream sensory and contextual inputs. Specifically, is there anatomical or functional evidence that A1 communicates context to wM2? What are the possible efferent targets of wM2 that execute the motor plan?

Extended Data Fig. 1: a-c: It would be helpful to Indicate units on y-axes and scale bar. Legend: “Eaxtended” → “Extended”. Legend for panel c: Is the right example illustrating a trial that was excluded due to movement during the delay period? If so, this could be more explicitly mentioned in the legend.

Fig.1:
c: It was unclear to me if this is pooled data from all 35 sessions or only from a subset of expert sessions. Moreover, it would be helpful if the authors describe the performance as a function of sessions and / or trials in a bit more detail.
d,e: I assume the color conventions are the same as in b,c. Could you clarify this in the legend?

Fig. 4:
This is a beautiful analysis. In panel b the authors show examples of clusters. It would be great for the readability to show alongside these examples also the breakdown of cortical identity and layer identity of the units, similar to panel a.

Fig. 5:
a: It would be helpful to indicate “Go-tone minus Nogo-tone firing rate” on the y-axis.

Methods:
Optogenetic stimulation of VGAT expressing neurons: can the authors comment or show data for the potential issue of stimulating VGAT neurons subcortically? The light probably travels to some extent beyond the cortex and may recruit inhibition in the subcortical structures underneath the stimulated cortical areas, which may contribute to the effects the authors observe on the licking behavior.

Reviewer #2

(Remarks to the Author)
In the manuscript titled "Contextual gating of whisker-evoked responses by frontal cortex supports flexible decision making", Ghaderi and colleagues develop a context-dependent whisker detection task to study how cortical regions gate the transformation of a whisker deflection into a decision to perform a correct motor response. Through photoinactivation experiments spanning multiple cortical regions, they demonstrate important roles for the whisker secondary motor cortex and the anterolateral motor cortex in task performance. They further performed multi-site electrophysiology across cortical regions to reveal prominent context representations in wM2 and ALM and claim that context and whisker sensory processing is first integrated in wM2, followed by ALM. Altogether, this manuscript identifies wM2 as a locus for the contextual gating of the transformation of whisker sensation into motor commands for goal directed licking.

This study presents new results on persistent activity in frontal cortex encoding context and is a timely characterization of how multiple cortical regions enable the gating of sensory input to prepare to perform a motor response. That said, the authors make broad claims (which are not always as strongly supported by their data) identifying the wM2 as the central node for the contextual gating of sensory information into action. Given their data, it seems like ALM plays a similar role in task execution and it is unclear whether wM2 truly represents the first locus of context-dependent whisker integration, as the manuscript claims.

Major:

While the care given to exclude confounds related to movements is appreciated, the manuscript would still benefit from a more careful examination of motor responses, particularly in the photoinactivation experiments. I could not find how the metric of "probability of lick" was calculated in the manuscript. Is it the probability of the tongue touching the lick port or is it the probability of tongue protrusion? If an animal licks and misses the spout, this scenario could suggest that the decision to lick (because of a go-tone whisker deflection pairing) was correctly made, but there was an impairment in the animal's ability to angle its tongue. It would be important to show whether there are any differences across these two metrics (% of spout contact and % of tongue protrusion) across cortical regions and adjust interpretations as needed.

For the photoinactivation experiments comparing wM2 and ALM, it may not be possible to clearly differentiate effects isolated to either ALM or wM2. Previous literature has shown that using a similar clear skull approach, high power photoinactivation results in the spike rate of neurons decreasing up to ~50% for up to a 2mm spread from the laser center (e.g. Li et al., eLife). This means that inactivation of rostral targets at [AP: 2.5, ML: 1.5] and wM2 at [AP: 2.0, ML: 1.0] could be significantly overlapping. Caudal targets are also closely spaced. Minimally, the spatial resolution of photoinactivation methods should be discussed.

At several points in the manuscript, results are reported in a manner that should be more rigorous. For example, it is stated that "Altogether, neuronal activity in the quiet, late delay period comparing Go-tone vs No go-tone trials appear to be most obviously different for deep-layer neurons in wM2 (Fig. 3d), which also form a large fraction of the neurons of Cluster #11 (Fig. 4a,b)." Its not clear if this conclusion is supported statistically. Large effects are also observed in the ALM and ALM units make up a large part of the units observed in cluster 11. Furthermore, ALM makes up a large percentage of cluster 25 which also showed prominent delay period firing only in Go-tone trials. It is therefore not clear that there is clear difference between results observed in the ALM and wM2. Similarly, it is stated that wM2 contains the largest proportion of Go-tone excited neurons during the delay period. This may be a true statement as worded, strictly speaking, but it is not clear if the there is a statistically supportable difference between ALM and wM2. The effect size is also small (3%) and it is not clear if such a difference is meaningful.

The authors state that "we found that significant context-dependent sensory processing first transiently appeared in wS2 at 15 ms, and then in wM2 at 20 ms after whisker stimulus (and all later time bins) and next in ALM at 25 ms after whisker stimulus (and all later times) (Fig. 7d)." The reported decoding accuracies all lie below or close to 55%, which is a very small effect size (the SVM is still largely unable to decode contexts). It is thus unclear what can be taken away from this analysis and whether claims can be made regarding the temporal order of context decoding in cortex.

It is not clear why, in the analysis starting on Line 397, data were projected onto context and whisker directions. Why not explicitly find a direction that is predictive of upcoming behavior? Such a direction could exist in the ALM, even though lick/no lick trials do not separate along other directions. Why would the authors expect lick/no-lick conditions to separate along the context mode?

Minor:

In Fig. 1d-f, it is not clear if some movements are obfuscated by the method of presentation. Movements that are variable across time, trials, sessions and animals will average out to smaller values than reliable 'instructed' movements. Scaling plots to the largest average movements during the response period makes it difficult to see if delay period movements differ from baseline. It might be helpful to highlight the parts of Figs d,e where whisker movements differ on quiet trials for visual clarity. Finally, there are no units indicated for movement amplitude. If units are arbitrary, that should be made explicit.

It seems as though the number of sessions/mice used in photoinactivating different regions varies considerably, which means that statistical power is likely highly variable across regions.

The language of 'causal involvement' and 'causal roles' is imprecise and inappropriate. How does 'causal involvement' differ from 'involvement?' Causality involves the notion of sufficiency, which is not captured here. As a side note, a randomly applied air-puff to the eyes could likely impair task performance. One is unlikely in that case to conclude that the eyes are 'causally involved' or even 'involved' in this behavior.

Not clear why ROC analysis was used to quantify the fraction of modulated units, rather than a direct statistical test of means. ROC analysis is typically used to quantify discriminability.

One wonders whether neurons were sampled uniformly with respect to depth, and if so, if spike rates were constant across depth with respect to the analysis of Figure 3.

In Fig. 3b it is currently hard to compare/contrast activity across regions during auditory-induced transients

On Line 273, a description of classification should mention explicitly the time period used for classification.

Language about attractors is hard to interpret. If there was a stable attractor, then a perturbation away from the attractor should decay back to the attractor state following the perturbation. Is there evidence of this in response to photoinhibition? Or do the authors refer to a different interpretation?

Details pertaining to how single units were classified to a given layer should be added to the methods.

In Fig. 7b it is not clear what error bars represent and this is not listed in the figure legend.

Version 1:

Reviewer comments:

Reviewer #1

(Remarks to the Author)
The authors substantially improved their manuscript through the revisions. All points were addressed. Congratulations on this excellent paper.

Reviewer #2

(Remarks to the Author)
I appreciate the authors' thorough revisions in response to my comments. The manuscript has improved substantially, and conclusions are now better supported. I find this to be an interesting and solid study and recommend acceptance. Below I include a number of suggestions for the Authors to further improve the manuscript.

1) In Supplementary Fig. 1a, it would be helpful to indicate which sessions belonged to the same animal.

2) In Supplementary Fig. 3, it would be helpful to label the regions on at least one of the spatial maps. They are in the main figure, but absent here.

3) I do not find the statement on lines 435-438, describing the data in Figure 7d, to provide compelling support for a specific role for wM2 vs. ALM. One should directly compare the distributions of threshold-crossing times in the two regions to determine whether they differ statistically. As written, the text is not strictly wrong, but it is somewhat misleading and not very principled, and I suggest improving this analysis or changing the language describing these data.

4) ROC analysis remains unusual. The analysis is likely to work for the intended purpose under some modest assumptions, but it seems an indirect way of accomplishing the intended goal. An ROC analysis is appropriate for determining how discriminable two distributions are. If one simply wants to determine whether the means of two groups are different, in a statistical sense, then any test of means (a t-test or nonparametric analog) would suffice. The analysis can be kept as-is, but

to me, suggests an incomplete understanding of analytical approaches.

5) Regarding the interpretation of data as suggesting an attractor state, the extra analysis is appreciated, but I am not sure that the overall treatment of attractor states provides any real insights here. Suppose a brain region exhibits persistent activity encoding a particular behavioral variable (context, for example). If this activity is driven in a purely feedforward manner - the result of some tonic input - then my understanding is that the analysis of flow fields would indicate a fixed point so long as there was some neuronal or measurement noise present to deviate the measured value away from the mean. The authors assert the existence of an attractor state in particular circuits (e.g. line 415), which to me means that the region itself could be considered a dynamical system with a fixed point. In this case, I do not see how such an assertion is supportable.

Alternatively, one could argue (in this thought experiment) that there must be an attractor present somewhere else in the brain to maintain the tonic input. Perhaps this is what the authors mean by 'attractor state.' This would then seem to equate any persistent activity that is observed anywhere in the brain (and that is not explainable by sensation or movement) with an attractor state. Perhaps this is not wrong, but the consideration of attractors provides no additional value in this interpretation above the empirical observation of persistent activity. In any case, I challenge the authors to consider the concept of attractors in a manner that is more conceptually precise. Unless I am missing some deeper meaning, this portion of the manuscript reads as a superficial analysis using buzzwords in a context without a lot of substance.

6) I appreciate the inclusion of more methodological details. Some, however, could be relegated to the Methods for readability (Lines 121-122, 188-196, etc.).

7) If not already specified, please state the response window used to quantify lick contacts (start and end times relative to the cue).

Open Access This Peer Review File is licensed under a Creative Commons Attribution 4.0 International License, which permits use, sharing, adaptation, distribution and reproduction in any medium or format, as long as you give appropriate credit to the original author(s) and the source, provide a link to the Creative Commons license, and indicate if changes were made.
In cases where reviewers are anonymous, credit should be given to 'Anonymous Referee' and the source.
The images or other third party material in this Peer Review File are included in the article's Creative Commons license, unless indicated otherwise in a credit line to the material. If material is not included in the article's Creative Commons license and your intended use is not permitted by statutory regulation or exceeds the permitted use, you will need to obtain permission directly from the copyright holder.
To view a copy of this license, visit https://creativecommons.org/licenses/by/4.0/

Comments from reviewers, cited directly from the decision email, are in Times New Roman italics. Our replies are in Arial.

Reviewer #1 (Remarks to the Author):

This manuscript by Ghaderi, Crochet, and Petersen presents a comprehensive and technically impressive investigation into how frontal cortex—particularly the whisker-related secondary motor cortex (wM2)—integrates contextual auditory information to gate somatosensory processing and guide flexible decision making in mice. The authors employ a novel behavioral paradigm requiring mice to use a brief auditory cue (Go or Nogo) to interpret an upcoming identical whisker stimulus and decide whether to lick for reward. The paper follows the tradition of the lab to expertly characterize the progression of activity over multiple cortical areas during sensory processing and task execution.

Combining precise spatiotemporal optogenetic inactivation with large-scale Neuropixels recordings, the authors delineate the contributions of distinct cortical areas across auditory, delay, and sensory epochs. Their results show that wM2 encodes context during the delay period through persistent activity, and that wM2 activity within 30 ms of whisker stimulation predicts the behavioral outcome, even in error trials. The findings position wM2 as a critical hub for context-dependent sensory-motor transformation.

The study provides significant conceptual advances by identifying wM2 as the site of context-sensory integration in a whisker-based decision-making task. While prior work has implicated ALM and broader frontal cortex in similar tasks, this manuscript clearly delineates the sequential and causal roles of wM2 and ALM. These findings are novel and compelling and the conclusions are well-supported, and the work will be of wide interest.

I have the following suggestions to further improve the manuscript before publication.

While the study makes a compelling case for wM2's role, it could benefit from a deeper discussion of how wM2 integrates upstream sensory and contextual inputs. Specifically, is there anatomical or functional evidence that A1 communicates context to wM2? What are the possible efferent targets of wM2 that execute the motor plan?

The reviewer makes a valuable point. In our study, we have not investigated how auditory information reaches wM2. However, there are many possible pathways, which we now briefly mention in a new paragraph of the Discussion section. One possible circuit mechanism might be direct projections from auditory cortex to secondary motor cortex. Indeed, a monosynaptic pathway from auditory cortex to secondary motor cortex was reported by Holy & Schneider (Cell Reports 43: 114396; 2024; https://doi.org/10.1016/j.celrep.2024.114396), and we now refer to this study. We also investigated the Allen Mouse Brain Connectivity Atlas, finding projections from primary auditory cortex (AUDp) to secondary motor cortex, co-localising in wM2 where whisker somatosensory cortex projects. We include illustrative figures from the Allen Mouse Brain Connectivity Atlas in Supplementary Figure 19. There are, of course, many other possible pathways, including prominent projections from retrosplenial cortex to wM2, which have recently been highlighted as potentially important for context-dependent processing of whisker-related information in a closely-related task (Bech, Dard et al., bioRxiv;

https://doi.org/10.1101/2025.10.31.685738). Furthermore, we now also briefly discuss likely additional contributions from thalamus, perhaps involving similar pathways to those described by Inagaki et al., 2022 (Cell 185: 1065; https://doi.org/10.1016/j.cell.2022.02.006).

In the Discussion section on lines 500-512, we now write: "*Auditory information could reach wM2 by many diverse neuronal pathways. Interestingly, previous work*[49] *points to a direct pathway from Auditory cortex to wM2, which is also supported by analysis of the Allen Mouse Brain Connectivity Atlas*[50] *(Supplementary Fig. 19). However, there are many alternative routes including a potentially important pathway from retrosplenial cortex to wM2, which has recently been suggested to play a role in a closely-related context-dependent whisker detection task*[51]*. Input to wM2 via subcortical pathways is also likely to contribute importantly to relaying auditory information to frontal cortex, for example through a recently described pathway involving neurons in midbrain reticular and pedunculopontine nuclei signalling to a thalamic region projecting to frontal cortex*[52]*. Future studies are therefore necessary to specifically investigate the synaptic circuit mechanisms underlying the origin of the auditory contextual signals observed in wM2 in our context-dependent whisker-detection task.*"

We also briefly point out diverse projections of wM2 neurons that need further investigation in the future, writing on lines 530-534: "*Distinct classes of excitatory neurons in wM2 project to many diverse cortical and subcortical targets, including ALM, which might contribute importantly. In future studies, it will be important to further define the essential subtypes of neurons in wM2 and which synaptic interactions drive the context-dependent gating of sensory processing in wM2.*"

Extended Data Fig. 1: a-c: It would be helpful to Indicate units on y-axes and scale bar. Legend: "Eaxtended" → "Extended". Legend for panel c: Is the right example illustrating a trial that was excluded due to movement during the delay period? If so, this could be more explicitly mentioned in the legend.

In the new version of "Supplementary Fig. 1", we have indicated that the y-axes in panel d (previous panel c) is in arbitrary units (a.u.), and we have improved the y-scale to make the movements easier to see. Neither of these trials was excluded. The last 200 ms of the delay period was without jaw and whisker movements (below the threshold) and this is now explicitly mentioned in the figure legend.

Fig.1:
c: It was unclear to me if this is pooled data from all 35 sessions or only from a subset of expert sessions. Moreover, it would be helpful if the authors describe the performance as a function of sessions and / or trials in a bit more detail.

We have improved the figure legend to make it clear that the data in Fig. 1c report all values across the 35 recording sessions.
We have also added a new panel to Supplementary Fig. 1 which shows the lick probability data for each session and each trial type individually as a color-coded matrix (SupFig. 1a).

d,e: I assume the color conventions are the same as in b,c. Could you clarify this in the legend?

We now explicitly state the color coding for panels d and e in the figure legend, which is indeed the same as panels b and c.

Fig. 4:
This is a beautiful analysis. In panel b the authors show examples of clusters. It would be great for the readability to show alongside these examples also the breakdown of cortical identity and layer identity of the units, similar to panel a.

We have improved the figure following the excellent suggestion of the reviewer. In panel b, we now add the Area, RS/FS, Layer, and Delay color bars relevant for the five example clusters.

Fig. 5:
a: It would be helpful to indicate "Go-tone minus Nogo-tone firing rate" on the y-axis.

We have added "Go minus Nogo ∆AP rate (Hz)" as the y-axis label.

Methods:
Optogenetic stimulation of VGAT expressing neurons: can the authors comment or show data for the potential issue of stimulating VGAT neurons subcortically? The light probably travels to some extent beyond the cortex and may recruit inhibition in the subcortical structures underneath the stimulated cortical areas, which may contribute to the effects the authors observe on the licking behavior.

The reviewer is correct to point to potential confounds in the optogenetic stimulation of GABAergic neurons with respect to cortical specificity. Specifically, the reviewer points to potential effects on subcortical structures, which in this case would be the striatum. More than 90% of neurons in the striatum are GABAergic, so this could be a major confound to the experimental manipulation. However, VGAT-ChR2 line 8 BAC transgenic mice (used in our study) do not express ChR2 in striatal projection neurons. In addition, light penetration is expected to be low at 1 mm below the cortical surface, given that blue light is highly scattered and absorbed by brain tissue.

Perhaps most relevant are the data from Babl et al. (Cell Reports 29: 1381; https://doi.org/10.1016/j.celrep.2019.09.049) who report that blue light delivered at the cortical surface does not activate neuronal firing in striatal neurons (see for example Figure 3F of their study, *reproduced below*). Even with 16 mW light at the cortical surface, there are no directly activated neurons in the underlying striatum. We therefore do not think that our optogenetic manipulations directly affect neurons in the striatum. However, the cortical inactivation is sure to have an indirect impact upon striatum, and indeed the recruitment of various striatal circuits could contribute importantly to task performance.

[Figure Redacted]

Figure 3F from Babl et al. (2019) Cell Reports 29: 1381.

https://doi.org/10.1016/j.celrep.2019.09.049

Reviewer #2 (Remarks to the Author):

In the manuscript titled "Contextual gating of whisker-evoked responses by frontal cortex supports flexible decision making", Ghaderi and colleagues develop a context-dependent whisker detection task to study how cortical regions gate the transformation of a whisker deflection into a decision to perform a correct motor response. Through photoinactivation experiments spanning multiple cortical regions, they demonstrate important roles for the whisker secondary motor cortex and the anterolateral motor cortex in task performance. They further performed multi-site electrophysiology across cortical regions to reveal prominent context representations in wM2 and ALM and claim that context and whisker sensory processing is first integrated in wM2, followed by ALM. Altogether, this manuscript identifies wM2 as a locus for the contextual gating of the transformation of whisker sensation into motor commands for goal directed licking.

This study presents new results on persistent activity in frontal cortex encoding context and is a timely characterization of how multiple cortical regions enable the gating of sensory input to prepare to perform a motor response. That said, the authors make broad claims (which are not always as strongly supported by their data) identifying the wM2 as the central node for the contextual gating of sensory information into action. Given their data, it seems like ALM plays a similar role in task execution and it is unclear whether wM2 truly represents the first locus of context-dependent whisker integration, as the manuscript claims.

We thank the reviewer for raising this important point. We agree with the reviewer that ALM likely plays an important role in this task, and in the revised manuscript we further stress the importance of ALM. Indeed, in many respects, ALM and wM2 appear to be very similar. However, in our analyses, we also found two key differences. Firstly, optogenetic inactivation during the presentation of the auditory contextual cue had a strong behavioral impact for wM2 inactivation but not for ALM inactivation (Figure 2). This suggests that relevant neuronal activity is present in wM2 before ALM. As described in more detail below, in the revised manuscript we have further strengthened our analyses of the inactivation data through addressing the thoughtful comments of the reviewer. Secondly, the decision to lick was encoded at an earlier timepoint in wM2 compared to ALM (Figure 8). In response to the reviewer comments (see below for more details), we have carried out further analyses that support the hypothesis that the earliest licking-decision signals at 30 ms after whisker deflection appear to be better represented in wM2 compared to ALM.

Major:

While the care given to exclude confounds related to movements is appreciated, the manuscript would still benefit from a more careful examination of motor responses, particularly in the photoinactivation experiments. I could not find how the metric of "probability of lick" was calculated in the manuscript. Is it the probability of the tongue touching the lick port or is it the probability of tongue protrusion? If an animal licks and misses the spout, this scenario could suggest that the decision to lick (because of a go-tone whisker deflection pairing) was correctly made, but there was an impairment in the animal's ability to angle its tongue. It would be important to show whether there are any differences across these two metrics (% of spout contact and % of tongue protrusion) across cortical regions and adjust interpretations as needed.

The probability of licking was defined as the probability of a tongue-spout contact in the reporting window, which also served as the on-line trigger for water delivery. We now explicitly mention this on lines 100-102 where we write: "*Licking was assessed online (and in all quantification of lick probabilities) as tongue-spout contacts measured through piezofilm attached to the reward spout.*"

The reviewer is correct to point out different failure modes, which is of particular importance in the optogenetic inactivation experiments. Unfortunately, we did not carry out video filming during optogenetic inactivation, and we therefore do not have information about the diverse ways in which the tongue might fail to make contact with the spout. This would be interesting and important to examine in future studies, and on lines 161-164 we now write: "*We did not carry out behavioral filming during the optogenetic inactivation experiments, so we were not able to distinguish different failure modes (e.g. decision vs motor), which would be of interest to investigate in future studies.*"

For the photoinactivation experiments comparing wM2 and ALM, it may not be possible to clearly differentiate effects isolated to either ALM or wM2. Previous literature has shown that using a similar clear skull approach, high power photoinactivation results in the spike rate of neurons decreasing up to ~50% for up to a 2mm spread from the laser center (e.g. Li et al., eLife). This means that inactivation of rostral targets at [AP: 2.5, ML: 1.5] and wM2 at [AP: 2.0, ML: 1.0] could be significantly overlapping. Caudal targets are also closely spaced. Minimally, the spatial resolution of photoinactivation methods should be discussed.

The reviewer brings up an important point. We agree with the reviewer that it is important to quantify the spatiotemporal dynamics of optogenetic inactivation, as was done in the careful study of Li et al., 2019, which we now cite. However, from a functional perspective, we have repeatedly found that blue light applied to cortical regions separated by ~1 mm give rise to different behavioral effects. Comparing blue light applied to wS1 and fpS1 (which are separated by ~1 mm) we found a profound impact on behavioral performance of inactivating wS1 but not fpS1, as shown in Figure 2 in this study, as well as in Oryshchuk et al., 2024 (Cell Reports, https://doi.org/10.1016/j.celrep.2023.113618). Equally, here we report distinct effects of inactivating ALM and wM2 (Figure 2), which are separated by ~1 mm. Differences in behavioral performance induced by inactivating ALM or wM2 were also observed in Esmaeili et al., 2021 (Neuron, https://doi.org/10.1016/j.neuron.2021.05.005), which used similar methodology to our current study.

Also, it is important to note that a difference with other studies is that we used a small diameter (200 µm) optic fiber with small NA (0.22) (compared to the 400 µm beam diameter in Li et al., 20149), thus likely limiting the lateral spread of the light.

In our revised manuscript we now discuss this important point on lines 198-211 where we write: "*Although optogenetic inactivation using blue light in VGAT-ChR2 mice has been reported to exert effects across a large spatial extent depending upon the precise stimulation parameters[38], in our experimental paradigm we observed clear differences comparing inactivation of cortical regions separated by ~1 mm. For example, fpS1 is located ~1 mm anterior to wS1 and inactivation of fpS1 did not affect behavioral performance unlike inactivation of wS1, similar to previous experiments carried out under the same conditions[27]. Equally, during the presentation of the auditory contextual cue, here we found significant (bootstrap FDR-corrected, p =*

0.009) differences in behavioral performance comparing inactivation of wM2 and ALM, which are also separated by ~1 mm. Thus, our optogenetic inactivation experiments appear to localize functional differences of neighbouring cortical regions separated by relatively small distances, similar to previous experiments carried out under the same conditions in which inactivation of ALM and wM2 also had differential effects[25]. In future experiments, it will be important to further quantify the spatiotemporal impact of our inactivation paradigm."

At several points in the manuscript, results are reported in a manner that should be more rigorous. For example, it is stated that "Altogether, neuronal activity in the quiet, late delay period comparing Go-tone vs No go-tone trials appear to be most obviously different for deep-layer neurons in wM2 (Fig. 3d), which also form a large fraction of the neurons of Cluster #11 (Fig. 4a,b)." Its not clear if this conclusion is supported statistically. Large effects are also observed in the ALM and ALM units make up a large part of the units observed in cluster 11. Furthermore, ALM makes up a large percentage of cluster 25 which also showed prominent delay period firing only in Go-tone trials. It is therefore not clear that there is clear difference between results observed in the ALM and wM2. Similarly, it is stated that wM2 contains the largest proportion of Go-tone excited neurons during the delay period. This may be a true statement as worded, strictly speaking, but it is not clear if the there is a statistically supportable difference between ALM and wM2. The effect size is also small (3%) and it is not clear if such a difference is meaningful.

In response to the reviewer's comment, we have tried to improve the reporting of our results. Specifically, the reviewer asks for statistical analyses comparing neuronal activity in ALM and wM2 during the late delay period. We have therefore carried out the following additional analyses, which are now reported in the Results section describing Figure 3:

i) In Figure 3c, we quantify the Go-tone-related neuronal activity in the last 200 ms of the delay period of quiet trials. Using a chi-squared statistical test, we find that the proportion of neurons that are positively modulated in our ROC analysis is statistically higher in wM2 compared to ALM. The fraction of up-modulated neurons in wM2 was 20.3% and in ALM was 16.8% which is significantly different according to a chi-squared statistical test with p-value of 0.0008. The fraction of down-modulated neurons in wM2 was 14.2% and in ALM was 16.5% which is significantly different according to a chi-squared statistical test with p-value of 0.015. These analyses are now reported in lines 257-265 where we write: "*We carried out statistical tests to examine differences comparing wM2 and ALM for the Go-tone-related neuronal activity in the last 200 ms of the delay period of quiet trials, finding that the proportion of neurons that are positively modulated in our ROC analysis is statistically higher in wM2 compared to ALM. The fraction of up-modulated neurons was significantly higher in wM2 (20.3%) than in ALM (16.8%) (p = 0.008, chi-squared test), whereas the fraction of down-modulated neurons was slightly but significantly lower in wM2 (14.2 %) compared to ALM (16.5%) (p = 0.015, chi-squared test). Both of these analyses support the overall stronger increased firing rate in wM2 compared to ALM in the late delay period of quiet trials following a Go-tone.*"

ii) In Figure 3d, we quantify neuronal activity for superficial vs deep neurons in various cortical regions recorded. We have now added statistical analyses comparing superficial vs deep neurons. In lines 275-279, we now write: "*Statistical tests indicated that neuronal activity during the late delay period of Go-tone trials differed significantly*

across cortical layers in wM2, with infragranular neurons having larger changes in firing rates than supragranular neurons (1.2 ± 0.1 Hz vs 0.03 ± 0.10 Hz; two-sample t-test, $p = 1.7 \times 10^{-7}$), whereas no significant differences were detected in A1, wS2, or ALM."

The authors state that "we found that significant context-dependent sensory processing first transiently appeared in wS2 at 15 ms, and then in wM2 at 20 ms after whisker stimulus (and all later time bins) and next in ALM at 25 ms after whisker stimulus (and all later times) (Fig. 7d)." The reported decoding accuracies all lie below or close to 55%, which is a very small effect size (the SVM is still largely unable to decode contexts). It is thus unclear what can be taken away from this analysis and whether claims can be made regarding the temporal order of context decoding in cortex.

The reviewer is correct to point out that the effects shown in Fig. 7d might be considered subtle. In the text, we simply report what is observed in our analyses, without making further claims. Decoding accuracies can of course be significant (or otherwise) for any specific value, and although we agree that 55% decoding accuracy is not very high, the precise value of the decoding accuracy depends on many aspects. In this specific analysis, it is worth noting the 5 ms temporal resolution, which as the reviewer might appreciate, greatly reduces decoding accuracy compared to binning neuronal activity over longer time scales, as is typically done in many studies, but which would preclude resolving the earliest effects.

It is not clear why, in the analysis starting on Line 397, data were projected onto context and whisker directions. Why not explicitly find a direction that is predictive of upcoming behavior? Such a direction could exist in the ALM, even though lick/no lick trials do not separate along other directions. Why would the authors expect lick/no-lick conditions to separate along the context mode?

The reviewer makes an important point and we have now followed the reviewer's suggestion to examine a lick coding direction. We have moved the previous context coding direction analyses presented in the old Figure 8b to Supplementary Fig. 17, because we think that the previous analysis might nonetheless be of interest to some readers. In the new version of Figure 8b, we now explicitly focus on analysis of the lick coding direction, finding, as before, that wM2 activity differentiates lick vs no-lick better than any other area we recorded from, with significant differences already at 30 ms. At later times (see new Supplementary Figure 18), analysed at 200-400 ms after whisker stimulus, when the mouse is licking in hit trials, the lick coding direction indicated equal or stronger representations in ALM compared to wM2. We thank the reviewer for the valuable suggestion to directly examine lick coding, which we think has strengthened our manuscript.

In lines 456-470 we now write: "*Finally, we directly tested neuronal activity in a lick coding direction, which we defined during bouts of spontaneous licking not cued by any sensory stimuli. Projecting neuronal activity in the lick coding direction revealed separation between lick and no-lick trials for both correct and false alarm cases in wM2 at 30 ms after delivery of the whisker stimulus (Figure 8b). The lick coding direction did not separate between lick and no-lick trials in A1, wS1 or wS2 at this early time point. Neuronal activity in ALM was higher in the lick coding direction only for correct*

trials, but false alarm lick trials were not different from no-lick trials at 30 ms post whisker stimulus time. These analyses therefore further support wM2 as a key cortical region that first predicts future lick initiation after the whisker stimulus independently of whether it is a correct or false alarm lick. Consistent with ALM also playing a prominent role in licking[46,47], at later times, neuronal activity in the lick coding direction shows significant differentiation between lick and no-lick trials in ALM, wM2, wS2 and wS1 ranked according to the effect size quantified at 0.2-0.4 s after the whisker stimulus with ALM being largest (Supplementary Fig. 18)."

Minor:

In Fig. 1d-f, it is not clear if some movements are obfuscated by the method of presentation. Movements that are variable across time, trials, sessions and animals will average out to smaller values than reliable 'instructed' movements. Scaling plots to the largest average movements during the response period makes it difficult to see if delay period movements differ from baseline. It might be helpful to highlight the parts of Figs d,e where whisker movements differ on quiet trials for visual clarity. Finally, there are no units indicated for movement amplitude. If units are arbitrary, that should be made explicit.

The reviewer is correct to point out that uninstructed movements will appear reduced after trial averaging. In Supplementary Fig. 1, we show single example trials to help the reader visualize movements initiated independently of sensory cues. The shaded region in Fig. 1e indicates the *post hoc* enforced quiet period (the last 200 ms of the delay window), which we think is clearly different from the data without selection of quiet trials represented in Fig. 1d. In particular the blue traces (representing Go tone trials) have increased whisker speed in the last period before the whisker stimulus.

Unfortunately, the movements were not calibrated and thus are shown in arbitrary units for the y-axis. This is now explicitly stated in the figure legend.

It seems as though the number of sessions/mice used in photoinactivating different regions varies considerably, which means that statistical power is likely highly variable across regions.

The number of sessions for each cortical area inactivation is now also described in the legend of Figure 2: "A1 (n = 19 sessions), wS1 (n = 20 sessions), wS2 (n = 20 sessions), wM2 (n = 14 sessions), ALM (n = 9 sessions), and fpS1 (n = 9 sessions)". We reanalysed the statistical significance of effects through subsampling and bootstrapping finding consistent differences, and in the text we have updated the stated p-values. On lines 165-172, we now write: "*Because the number of inactivation sessions varied across cortical regions, we controlled for unequal sample sizes by subsampling each region to a common minimum number of 9 sessions across all comparisons. Statistical significance was assessed using paired bootstrap resampling (1,000 iterations), in which session-level lick probabilities from the no-light control and optogenetic inactivation conditions were resampled with replacement, and the statistic of interest was recomputed for each iteration. Bootstrap p-values were corrected for multiple comparisons using false discovery rate correction within each stimulation window.*"

The language of 'causal involvement' and 'causal roles' is imprecise and inappropriate. How does 'causal involvement' differ from 'involvement?' Causality involves the notion of sufficiency, which is not captured here. As a side note, a randomly applied air-puff to the eyes could likely impair task performance. One is unlikely in that case to conclude that the eyes are 'causally involved' or even 'involved' in this behavior.

We have removed the word 'causal' from our manuscript.

Not clear why ROC analysis was used to quantify the fraction of modulated units, rather than a direct statistical test of means. ROC analysis is typically used to quantify discriminability.

ROC analysis is widely used in the community and only differs slightly from direct statistical tests, but better takes into account trial-to-trial variability, offers a meaningful metric (SI) and statistics (p-value).

One wonders whether neurons were sampled uniformly with respect to depth, and if so, if spike rates were constant across depth with respect to the analysis of Figure 3.

The *Neuropixels* probes covered the entire depth of the cortex, so in that sense they were not biased to measuring neuronal activity from any specific depth. However, the detection of single units through the spike sorting algorithm relies on high-amplitude extracellular spikes and relatively high firing rates. To help the reader better understand the variation in firing rates across cortical depth in our analyses, in Supplementary Fig. 8, we now show the mean firing rates across supragranular, granular and infragranular without baseline subtraction. In general, firing rates were found to be slightly higher in deeper layers, but the small effect size in wM2 and ALM make it seem unlikely to be a major contributing factor to any of our reported results.

In Fig. 3b it is currently hard to compare/contrast activity across regions during auditory-induced transients

To help the reader, we have made a new Supplementary Fig. 6, in which we show firing rates of neurons across different cortical regions and trial types at higher temporal resolution around the time of the auditory and whisker stimulation times.

On Line 273, a description of classification should mention explicitly the time period used for classification.

On lines 253-257, we now explicitly write: "*We used ROC analysis to quantify the fraction of modulated units contrasting the firing of correct Go-tone Whisker trials in each cortical region during the same four trial epochs as described above: Auditory (0 – 0.03 s), Delay (0.8 – 1 s), Whisker (1 – 1.03 s) and Licking (1.3 – 1.5 s) relative to baseline activity (Fig. 3c).*"

Language about attractors is hard to interpret. If there was a stable attractor, then a perturbation away from the attractor should decay back to the attractor state following the perturbation. Is there evidence of this in response to photoinhibition? Or do the authors refer to a different interpretation?

We agree that perturbations are excellent tests for attractor states. Here, we refer to attractor states reflected as persistent activity in a stable network state. To help the reader understand our thinking and following discussions with Dr. Lorenzo Fontolan whose help we acknowledge, we investigated the gradients of neuronal activity dynamics in a new analysis presented in Supplementary Fig. 13. In our analyses, we exploit spontaneous fluctuations in neuronal state space to map the convergent neuronal dynamics towards fixed points. This analysis defines a vector field of gradients in neuronal activity space that corresponds to the energy landscape surrounding the stable fixed points. Although much further work could be done to examine details of the stable state at the end of the delay period, we think the current evidence supports an attractor state.

Details pertaining to how single units were classified to a given layer should be added to the methods.

We have improved the Methods section to specify that the layer assignments were based on the estimated recording location after applying the *Universal Probe Finder* tool and aligning to the Allen Mouse Brain Atlas (CCF v3) parcellations.

In Fig. 7b it is not clear what error bars represent and this is not listed in the figure legend.

We now state in the figure legend that “The PSTHs show mean (solid line) ± sem (shading).”

Comments from reviewers, cited directly from the decision email, are in Times New Roman italics. Our replies are in Arial.

Reviewer #1 (Remarks to the Author):

The authors substantially improved their manuscript through the revisions. All points were addressed. Congratulations on this excellent paper.

We thank the reviewer for his time and help in improving the manuscript.

Reviewer #2 (Remarks to the Author):

I appreciate the authors' thorough revisions in response to my comments. The manuscript has improved substantially, and conclusions are now better supported. I find this to be an interesting and solid study and recommend acceptance. Below I include a number of suggestions for the Authors to further improve the manuscript.

1) In Supplementary Fig. 1a, it would be helpful to indicate which sessions belonged to the same animal.

We now indicate the mice in Supplementary Fig. 1a.

2) In Supplementary Fig. 3, it would be helpful to label the regions on at least one of the spatial maps. They are in the main figure, but absent here.

We now indicate the region names in Supplementary Fig. 3.

3) I do not find the statement on lines 435-438, describing the data in Figure 7d, to provide compelling support for a specific role for wM2 vs. ALM. One should directly compare the distributions of threshold-crossing times in the two regions to determine whether they differ statistically. As written, the text is not strictly wrong, but it is somewhat misleading and not very principled, and I suggest improving this analysis or changing the language describing these data.

The original sentence referred to by the reviewer stated: "*Applying a support vector machine decoding strategy (as before, but now for 5 ms time bins with baseline subtraction) for classifying correct Go vs Nogo trials, we found that significant context-dependent sensory processing first transiently appeared in wS2 at 15 ms, and then in wM2 at 20 ms after whisker stimulus (and all later time bins) and next in ALM at 25 ms after whisker stimulus (and all later times) (Fig. 7d).*" This text describes the data shown in Fig. 7d, and, as the reviewer agrees, is not "*wrong*". We think the reviewer would prefer the sentence to have less comparative implications, and we therefore changed the sentence to (P. 14 Lines 436-442 of the revised manuscript): "*Applying a support vector machine decoding strategy (as before, but now for 5 ms time bins with baseline subtraction) for classifying correct Go vs Nogo trials, we found that significant*

context-dependent sensory processing transiently appeared in A1 and wS2 at 15 ms after whisker stimulus for a single 5 ms-time bin; and appeared in a more lasting manner in wM2 at 20 ms after whisker stimulus (and all later time bins) and in ALM at 25 ms after whisker stimulus (and all later times) (Fig. 7d)."

4) ROC analysis remains unusual. The analysis is likely to work for the intended purpose under some modest assumptions, but it seems an indirect way of accomplishing the intended goal. An ROC analysis is appropriate for determining how discriminable two distributions are. If one simply wants to determine whether the means of two groups are different, in a statistical sense, then any test of means (a t-test or nonparametric analog) would suffice. The analysis can be kept as-is, but to me, suggests an incomplete understanding of analytical approaches.

We agree with the reviewer that ROC analysis is "*appropriate for determining how discriminable two distributions are*" and that is exactly what we use it for. We agree that other analyses could also be used, but ROC analysis has the advantage of providing a selectivity index on a comparable scale for each neuron.

5) Regarding the interpretation of data as suggesting an attractor state, the extra analysis is appreciated, but I am not sure that the overall treatment of attractor states provides any real insights here. Suppose a brain region exhibits persistent activity encoding a particular behavioral variable (context, for example). If this activity is driven in a purely feedforward manner - the result of some tonic input - then my understanding is that the analysis of flow fields would indicate a fixed point so long as there was some neuronal or measurement noise present to deviate the measured value away from the mean. The authors assert the existence of an attractor state in particular circuits (e.g. line 415), which to me means that the region itself could be considered a dynamical system with a fixed point. In this case, I do not see how such an assertion is supportable.

Alternatively, one could argue (in this thought experiment) that there must be an attractor present somewhere else in the brain to maintain the tonic input. Perhaps this is what the authors mean by 'attractor state.' This would then seem to equate any persistent activity that is observed anywhere in the brain (and that is not explainable by sensation or movement) with an attractor state. Perhaps this is not wrong, but the consideration of attractors provides no additional value in this interpretation above the empirical observation of persistent activity. In any case, I challenge the authors to consider the concept of attractors in a manner that is more conceptually precise. Unless I am missing some deeper meaning, this portion of the manuscript reads as a superficial analysis using buzzwords in a context without a lot of substance.

In Supplemental Fig. 13, we show the attractor fields underlying the persistent dynamics of the late delay period, revealing important gradients in wM2 and ALM. We agree with the reviewer, that these data do not provide mechanistic insight into how the attractor fields are shaped, i.e. whether they are internally generated within wM2 and/or ALM, or whether other brain regions might contribute. Further experimental and analytical work will be required in order to determine the biophysical mechanisms.

6) I appreciate the inclusion of more methodological details. Some, however, could be relegated to the Methods for readability (Lines 121-122, 188-196, etc.).

We think "*more methodological details*" help the reader understand our experiments and analysis better and we would prefer to keep them in the main text.

7) If not already specified, please state the response window used to quantify lick contacts (start and end times relative to the cue).

The response window started immediately after the whisker stimulus and lasted 1 s. This is indicated in the text on P. 4 Lines 107-109 where we write : "If the mouse licked the spout within a 1 s response window following a whisker stimulus that was preceded by a Go-tone, it received a drop of water (~5 μl) as reward."
